# A GoldenBraid cloning system for synthetic biology in social amoebae

**DOI:** 10.1093/nar/gkaa185

**Published:** 2020-03-30

**Authors:** Peter Kundert, Alejandro Sarrion-Perdigones, Yezabel Gonzalez, Mariko Katoh-Kurasawa, Shigenori Hirose, Peter Lehmann, Koen J T Venken, Gad Shaulsky

**Affiliations:** 1 Genetics & Genomics Graduate Program, Baylor College of Medicine, Houston, TX, USA; 2 Medical Scientist Training Program, Baylor College of Medicine, Houston, TX, USA; 3 Verna and Marrs McLean Department of Biochemistry and Molecular Biology, Baylor College of Medicine, Houston, TX, USA; 4 Department of Molecular and Human Genetics, Baylor College of Medicine, Houston, TX, USA

## Abstract

GoldenBraid is a rapid, modular, and robust cloning system used to assemble and combine genetic elements. *Dictyostelium* amoebae represent an intriguing synthetic biological chassis with tractable applications in development, chemotaxis, bacteria–host interactions, and allorecognition. We present GoldenBraid as a synthetic biological framework for *Dictyostelium*, including a library of 250 DNA parts and assemblies and a proof-of-concept strain that illustrates cAMP-chemotaxis with four fluorescent reporters coded by one plasmid.

## INTRODUCTION

Experimental research of the social amoeba *Dictyostelium discoideum* is highly significant in basic and translational biology and *D. discoideum* holds immense value as a model organism because of its unique transition from unicellular growth to multicellular development and the tractability of its growth and developmental cycles (1). The availability of a sequenced and annotated genome and the vast genomic resources (2) make *D. discoideum* highly desirable for systems biology approaches. Recently developed technologies include vectors for handling the AT-rich genome and improved methods for generating knock-in mutations by homologous recombination (3,4), vectors to manipulate wild strains (5), and CRISPR–Cas9 genome editing (6). Unfortunately, the field lacks technologies for high throughput standardized assembly of genetic elements, and the reliance on standard cloning approaches limits the utility of *D. discoideum* compared to other microbial models. Increased cloning throughput would expand the types of questions researchers could ask and increase the overall rate of genetic analysis. Standardized cloning would facilitate exchange of genetic elements between researchers and the subsequent development of novel technologies.

**Figure 1.**
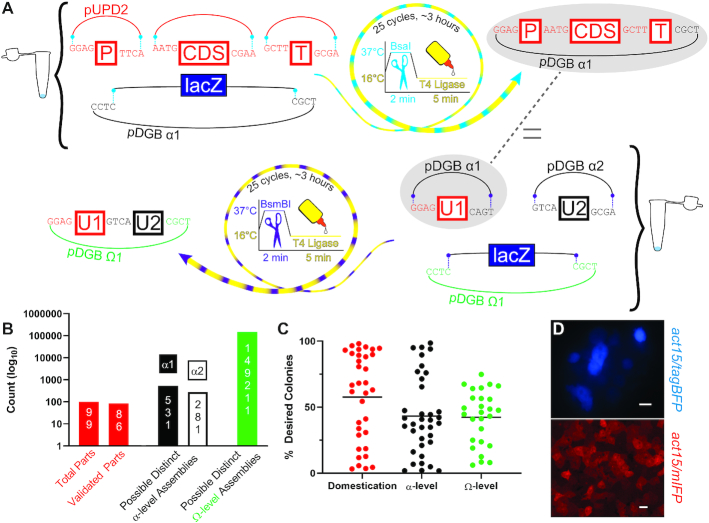
Adaptation of GoldenBraid as a rapid, modular and robust system for novel applications in *D. discoideum*. (**A**) In a single test tube in a matter of hours, a GoldenBraid reaction can assemble a transcriptional unit into an α-level backbone from many (even >7) individual DNA parts (top; P = promoter, CDS = coding DNA sequence, T = terminator). In a second, similar reaction, 2–5 α-level units can be combined into a single Ω-level vector backbone (bottom; U = transcriptional unit). Two Ω-level inserts comprising multiple transcriptional units can then be combined into an α-level backbone and so on, allowing iterative assembly until vector capacity becomes limiting (not shown). pUPD2 (red line) is the backbone that contains individual domesticated genetic elements (red boxes). The α-level backbones are denoted as black lines and the Ω-level backbones are denoted in green. Four-base sequences denote the grammar, *lacZ* denotes the *E. coli* beta-galactosidase gene, which is used in the blue-white screening. The striped arrows and the symbols they surround indicate the thermal cycles and the enzymes: ligase at 16°C in yellow, and BsaI and BsmBI at 37°C in magenta and purple, respectively. (**B**) Because of GoldenBraid's modularity, the present library of 99 GoldenBraid *D. discoideum* genetic elements (red bars) can be used to generate hundreds of transcriptional units (black and white bars, α1- and α2-levels, respectively), and hundreds of thousands of assemblies (green bar). The y-axis is in logarithmic scale and values are indicated inside each bar. (**C**) Cloning attempts (97) harboring properly assembled vectors are plotted such that each circle is an independent attempt, and the y-axis shows the fraction of desired clones (% of white colonies over total). In general, we miniprepped 2–4 clones corresponding to each GoldenBraid reaction. In all cases, at least one miniprepped clone was assembled correctly as determined by restriction enzyme fingerprinting. Colors match the above cloning level as indicated on the x-axis and the horizontal black lines indicate the average. (**D**) We adapted two new fluorescent proteins as indicated on the right of each panel, transformed them into *D. discoideum* cells and photographed the cells to illustrate proper expression and fluorescence. tagBFP scale bar = 10 μm; mIFP scale bar = 20 μm.

GoldenBraid cloning is ideal for *D. discoideum* research. Originally developed for plant synthetic biology (7,8), it is now in its third iteration (9) and has been adapted for use in human cell lines (10), fungi (11) and yeast mitochondria (12). GoldenBraid harnesses the power of type IIS restriction enzymes to increase cloning throughput, modularity, and robustness. These enzymes cut DNA at positions adjacent to their recognition sequences, which can generate user-defined sticky ends. GoldenBraid uses BsmBI and BsaI, two type IIS restriction enzymes that generate 4-base sticky ends. The specific sequences that comprise these sticky ends are incorporated into the GoldenBraid grammar, which is standardized in two ways. First, the grammar defines the identity of individual genetic elements, such as promoter, coding DNA sequence (CDS) and terminator. This grammar allows modular assembly of individual elements into more complex functional elements, such as a transcriptional unit that contains a promoter, a CDS and a terminator, in that order. Second, GoldenBraid consists of standardized vector backbones that allow rapid combination of complex genetic elements (Figure 1A). In a given GoldenBraid backbone, a pair of BsmBI and BsaI restriction sites and their adjacent 4 bp grammars are present in a specific, braided orientation on either side of an insert. This orientation supports one-pot, combined restriction, digestion and ligation reactions that enrich for a desired plasmid product over several cycles of cutting and ligation (7).

Here we describe an adaptation of GoldenBraid for use in *D. discoideum* as a system of clones and protocols for rapid and efficient assembly of commonly used genetic elements. We provide a starter kit of 99 modular genetic elements and 156 useful transcriptional units that are publicly available through the *Dictyostelium* Stock Center. We also provide two new fluorescent proteins on both sides of the current spectrum, blue and infra-red, all the commonly used drug selectable markers, several commonly used promoters and terminators, epitope tags and molecular barcodes. We show that assembly is highly efficient and simple, and we demonstrate the utility of the system by assembling 4 fluorescent-protein fusions from 18 independent fragments in one vector. Expressing these protein fusions in one *Dictyostelium* strain allows monitoring of cAMP signals and responses in live cells during streaming aggregation.

## MATERIALS AND METHODS

### PCR amplification of GoldenBraid parts and patches

We designed oligonucleotide primers (purchased from IDT, Coralville, IA, USA) to amplify and to add grammatical extensions to each part as described (7,8). If a given part contained one or more BsmBI or BsaI restriction sites, we used the GB Domesticator Tool to design additional primers to amplify the part as patches, which are multiple linear DNA pieces that can be ligated together using BsmBI or BsaI to generate a complete element (gbcloning.upv.es). A summary of all DNA templates is provided in Supplementary Table S2 and primer sequences are provided in Supplementary Table S3. We used Phusion DNA polymerase (NEB, Ipswich, MA, USA) in all PCR reactions, and employed a standard touchdown PCR protocol (13). We purified the PCR amplicons by agarose gel electrophoresis followed by extraction with a Zymo gel DNA extraction kit (Zymo Research, Irvine, CA, USA). For short elements (noted in Supplementary Table S3), we ordered complementary single stranded DNA oligonucleotides (IDT) and annealed them according to the manufacturer's recommended protocol. For parts that were not already codon-optimized for *D. discoideum*, we optimized the first 8–10 amino acids, which are the most important for efficient expression of transgenes (14) using the forward PCR primer.

### Domestication of GoldenBraid parts

We incubated 40–75 ng of a given amplified part or patch with 75 ng of the parental backbone pUPD2 (8), *Bsm*BI restriction enzyme (NEB), and T4 DNA ligase in 1× T4 DNA ligase buffer (Promega, Madison, WI, USA). We performed all GoldenBraid reactions in a thermocycler set for 25 cycles, each consisting of two steps: 2 min at 37°C followed by 5 min at 16°C.

### Generation of GoldenBraid backbones containing *D. discoideum* selectable markers

We designed primers to PCR amplify each GoldenBraid assembly unit (α1, α2, αB, αC, αD, αE, Ω1 and Ω2) containing *lacZα*, each bacterial- and amoebal-specific antibiotic resistance cassette and the ColE1 ori from pre-existing GoldenBraid vectors. We added extensions with these primers to allow assembly of these amplicons into circular plasmids in a GoldenBraid-like reaction with the type IIS restriction enzyme BbsI-HF. We validated the assemblies by restriction enzyme mapping and Sanger sequencing.

### Multipartite reactions to assemble and combine transcriptional units

We performed these reactions similarly to the domestication reactions, unless otherwise noted. For assembling initial transcriptional units into α-level vectors, we used 40 ng of the appropriate domestication-level parts, 75 ng of the desired α-level vector, and BsaI restriction enzyme (NEB). For combining initial transcriptional units into Ω-level vectors, we used 40 ng of each α-level transcriptional unit, 75 ng of the desired Ω-level vector, and BsmBI restriction enzyme.

### Bacterial strains and culture conditions

We used *E. coli* strains DH10β and DH5α (NEB) as bacterial hosts. Both strains worked equally well and we used them interchangeably. We used LB agar selection plates (100 μg/ml carbenicillin for the pUPD2 backbone, 30 μg/ml kanamycin for α-level backbones and 12.5 μg/ml chloramphenicol for Ω-level backbones) to isolate transformed bacterial clones. To allow blue-white screening, we top-plated 50 μl of 0.1 M IPTG and 50 μl of 20 mg/ml X-gal. We used LB media with the same antibiotic concentrations for propagation of individual bacterial clones.

### Preparation and transformation of chemically competent bacteria

We prepared chemically competent cells as described (15) with the exception of substituting potassium chloride for rubidium chloride.

### Preparation of bacterial frozen stocks

We made bacterial frozen stocks using LB + 15% glycerol as described (16).

### Isolation of plasmid DNA

We used standard column preps (Qiagen, Germantown, MD, USA) to isolate plasmid DNA from overnight bacterial cultures. We regenerated spin columns for repeated uses as described (17).

### Quantification of cloning reaction efficiencies

Using a Zeiss Stemi SV11 microscope with a mounted AxioCam ICc 3 and AxioVision 4.8 software (Carl Zeiss Microscopy, LLC, White Plains, NY, USA), we imaged one field of bacterial colonies per 6-cm transformation plate for 34 domestication reactions, 36 α-level reactions, and 27 Ω-level reactions. Transformation plates contained appropriate antibiotic were top-plated with 50 μl of 40 mg/mL X-gal in DMF or DMSO and 50 μl of 0.1 M IPTG for blue-white screening. We counted the parental (blue) and desired (white) colonies using the ImageJ 1.52e software Cell Counter plug-in and the Bio-Formats plugin, and plotted the results using the GraphPad Prism 8.3.0 software (GraphPad Software, San Diego, CA, USA).

### Restriction enzyme fingerprinting and Sanger sequencing

We validated all the plasmids by restriction-enzyme fingerprinting by incubating the plasmid with the appropriate enzyme(s) (NEB) for at least 30 min before electrophoresis on agarose gels and imaging. We obtained Sanger sequences by using the M13F and/or M13R universal primers and additional internal sequencing primers (Supplementary Table S2) as necessary.

### Amoebal strains and culture conditions

We used *D. discoideum* strain AX4 (18). We grew and maintained the amoebae as described (19). Briefly, we thawed frozen stocks of cells onto SM agar plates in association with *Klebsiella pneumoniae*. We picked amoebae from a single plaque and transferred them into a Petri dish of HL5 media (20) + PSV (21). Once cells had reached semi-confluence, we transferred them to a shaking flask of HL5+PSV. We maintained them in shaking suspension at 20°C in log-phase, at or below ∼5 × 10^6^ cells/ml for up to 30 days.

### Transformation of *D. discoideum*

For each transformation, we collected between 5 × 10^6^ and 1 × 10^7^ AX4 cells, and washed and resuspended them in 720–800 μl of EP buffer (10 mM NaPO_4_ pH 6.1 and 50 mM sucrose) (Supplementary Protocols). We added 5–15 μg of plasmid DNA in <80 μl of water to bring the total volume to 800 μl. We placed this solution in a 4-mm gap electroporation cuvette (Thermo Fisher Scientific, Waltham, MA, USA) and electroporated using a BTX ECM 630 electroporator (BTX, Holliston, MA) with the following settings: exponential decay, 1000 V, 25 Ω, 50 μF, with 2 pulses administered ∼5 s apart. After electroporation, we transferred cells to HL5 media + PSV in petri dishes. Sixteen to 24 h later, we added the appropriate antibiotic for selection (10 μg/ml G418, 20 μg/ml hygromycin, or 4 μg/ml blasticidin S). We changed the media every 3–4 days to maintain selection until colonies of resistant cells appeared. When the cultures were semi-confluent, we moved them to shaking culture in HL5 + PSV and antibiotic at the same concentration. Unless otherwise indicated, the analyses were done on mixed populations, because the goal was to validate the constructs rather than characterize the cell lines.

### Isolation of *D. discoideum* clones

In select instances, we isolated one or more clones by diluting ∼30 individual transformants in HL5 + PSV + antibiotic into the wells of a 96-well plate such that the populations of cells that appeared in roughly a third of the wells likely originated from single cells. We transferred cells from positive wells to larger dishes and shaking cultures as described above.

### Preparation of amoebal frozen stocks

We washed cells in HL5 without PSV, transferred them to freezing media in cryogenic tubes (50% FBS, 40% HL5 without PSV, 10% DMSO) and placed in –80°C. Supplementary Table S2 includes information about the non-clonal strains of amoeba that we prepared. Supplementary Table S4 is a summary of the clonal populations we prepared.

### Combinatorics

GoldenBraid elements were first assigned to functional categories. Approximate numbers of functional categories were counted for assembly reactions into the α1 backbone according to the number of parts assembled. Similar counts were taken for assembly reactions into the α2 backbone, with the single change that the number of functional classes of CDS’s were reduced by one as a strict assumption to ensure functional non-redundancy. Counts of possible functionally distinct α1 and α2 assemblies were multiplied to generate an approximate count of functionally distinct Ω-level assemblies. More detail is provided in Supplementary Table S5.

### CRISPR editing

We used CRISPOR to design sgRNA’s (http://crispor.tefor.net/) because it provides potential off-target information for *D. discoideum*. We ordered and annealed ssDNA oligonucleotides for the sgRNA’s according to the manufacturer's protocol (IDT). We cloned the oligonucleotides into the pDGB_A2_CRISPR1 backbone using a GoldenBraid-like reaction with the type IIS enzyme BbsI-HF, then transformed *D. discoideum* and selected for edited clones using G418 as described for the original *D. discoideum* CRISPR vector pTM1285 (3). We ensured that *D. discoideum* transformants were only transiently transformed with Cas9-expressing plasmid by re-exposing them to G418 selection and noting sensitivity after clonal isolation on SM plates with *Klebsiella pneumoniae* food bacteria. We imaged clonal isolation plates using a Zeiss Stemi SV11 microscope with a mounted AxioCam ICc 3 and AxioVision 4.8 software. We determined the specific CRISPR-induced mutations by colony PCR using primers flanking the corresponding PAM sequences followed by Sanger sequencing.

### Western blot analysis

We subjected cell lysates from AX4 (negative control), and strains expressing PkaC-HA (positive control), and GoldenBraid-assembled HA-jGCaMP7s (test) to SDS-PAGE through 12% gels (Bio-Rad, Hercules, CA, USA). We electro-transferred the proteins to nitrocellulose membranes in Tris/methanol transfer buffer (25 mM Tris [pH 7.6] and 20% (v/v) methanol) at 100 V for 1 h. We blocked the membranes with Tris-buffered saline with Tween 20 (TBST; pH 7.6, 0.5% Tween 20) and 5% nonfat milk for 1 h at room temperature, incubated with primary antibody (mouse anti-HA antibody, 1:500 dilution, BioLegend (San Diego, CA, USA); anti-HA.11, clone 16B12, Lot B220767) for 1 h at room temperature. We washed the blots with TBST three times for 10 min each and incubated with secondary horseradish peroxidase-conjugated goat anti-mouse IgG antibodies (Thermo Fisher Scientific) for 1 h at 1:10,000 dilution. We repeated the wash and developed the blots with a chemiluminescent substrate kit (SuperSignal WestPico, Thermo Fisher Scientific) according to the manufacturer's recommended protocol.

### Development of *D. discoideum* under agar

We collected cells (*coaA/flamindo2, coaA/pinkflamindo*, or the 4-color strain) from their HL5 growth media, washed and resuspended them in PDF buffer (1.5 g KCl, 1.6 g K_2_HPO_4_, 1.8 g KH_2_PO_4_ in 1 l water; pH 6.4; autoclaved, then added 1 ml 1 M CaCl_2_ and 2.5 ml 1 M MgSO_4_), and counted them using a hemocytometer. We placed 5 × 10^6^ cells in PDF onto a surface of KK2 (2.2 g KH_2_PO_4_ and 0.7 g K_2_HPO_4_ per 1 l) + 2% Noble agar (Difco, Franklin Lakes, NJ, USA) in a 3.5 cm Petri dish (Corning, NY, USA). We allowed the cells to settle on the agar surface for >10 min, then wicked away the PDF solution. We used a razor blade to cut out a ∼1 cm × ∼1 cm square of agar and flipped it onto a large circular glass coverslip (MatTek, Ashland, MA, USA) to sandwich the cells between the agar and glass as a near monolayer to visualize the cells with minimal vertical cell stacking.

### Fluorescence microscopy

We captured all fluorescent microscopic images using a Nikon (Tokyo, Japan) Eclipse Ti microscope. All images from a given technical replicate were exposed equally per channel, and the same lookup tables (LUTs) were applied to each channel across images. For the 4-color strain, we used the NIS Elements 4.51.00 software's spectral un-mixing feature to reduce the spectral overlap (especially between mCerulean and sfGFP) present in the raw images. Metadata for all fluorescent microscopic images and videos are provided in Supplementary Table S6.

### Annotation and statistical analysis of the 4-color strain

**Figure 2.**
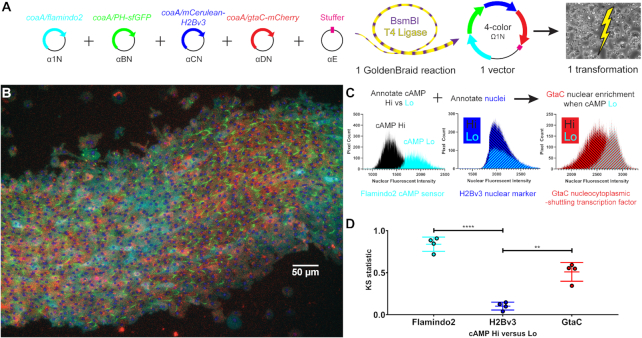
Validation of GtaC nuclear translocation in response to cAMP waves: GoldenBraid cloning and generation of a *D. discoideum* strain that expresses four fluorescent protein fusions. (**A**) A single expanded α-to-Ω assembly reaction was used to generate a vector with four transcriptional units, all driven by copies of the *coaA* promoter. Flamindo2 is a single-channel, intracellular cyclic AMP (cAMP) sensor that fluoresces more intensely as cAMP levels decrease (Citrine, pseudocolored cyan). The pleckstrin-homology (PH) domain of CRAC localizes to the leading edge membrane of polarized cells (sfGFP, pseudocolored green). mCerulean-H2Bv3 marks nuclei (pseudocolored blue). GtaC is a transcription factor that shuttles between the nucleus and cytoplasm every ∼6 minutes in response to cAMP waves (mCherry, pseudocolored red). The single plasmid was introduced into *D. discoideum* by electroporation in a single transformation. (**B**) An under-agar image of a stream of aggregating, 4-colored *D. discoideum* cells demonstrates a cAMP wave. PH-sfGFP localizes to sharp, leading-edge crescents especially in areas of high cAMP (low cyan fluorescence). Nuclei are seen as blue dots. Bar = 50 μm. Images of the same field in the 4 separate channels are provided in Supplementary Figure S4. (**C**) Binary annotation of nuclei in areas of high versus low cAMP allows comparison of GtaC nuclear fluorescent intensities. Histograms from one representative image (764 hi-cAMP nuclei, 581 lo-cAMP nuclei) are shown. (**D**) Regions of interest (ROIs) in nuclei were marked using the mCerulean-H2Bv3 fusion protein in the CFP channel. We then used a binary layer based on the Flamindo2 probe in the YFP channel to annotate these ROIs as high versus low intracellular cAMP levels. We combined these binary annotations to generate ROIs of nuclei in areas of high and low cAMP levels. Finally, we quantified the GtaC-mCherry fluorescence in each ROI. We performed this analysis for two images for each of two biological replicates containing 206–1062 nuclei in each cAMP condition. Colored lines indicate mean ± SD; Brown-Forsythe ANOVA (*F* = 74.55) and Dunnett's T3 multiple comparisons tests of Kolmogorov–Smirnov test statistics (individual colored dots, values indicated on the Y-axis) comparing Flamindo2 to H2Bv3 (*t* = 15.16) and GtaC to H2Bv3 (*t* = 6.721); *N* = 4 images taken over two biological replicates; ****P ≤ 0.0001; **P = 0.0047).

Nucleocytoplasmic shuttling of GtaC was not as pronounced as in the previously published, complemented strain *gtaC^–^[act15/GFP-gtaC]* (22). We therefore used the NIS Elements software binary functionality to mark regions of interest (ROI’s) in nuclei using the mCerulean-H2Bv3 fusion protein in the CFP channel. We then used another binary layer based on the Flamindo2 probe in the YFP channel to annotate ROI’s of high versus low intracellular cAMP levels. We combined these binary annotations to yield ROI’s of nuclei in areas of high and low cAMP levels. We performed this analysis for two images for each of two biological replicates containing 206–1062 nuclei in each cAMP condition. We exported fluorescent intensity histogram data from these high- and low-cAMP nuclei to Microsoft Excel for Office365 Version 1902 (Microsoft, Redmond, WA, USA) and GraphPad Prism version 8.3.0. In Prism, we generated the histograms shown in Figure 2C. In Excel, we used the Real Statistics Resource Pack add-in to convert the histogram data to raw values, then imported the values into Prism for statistical analysis. We performed Kolmogorov-Smirnoff tests to compare the distributions of the fluorescent intensity values between high- and low-cAMP nuclei for mCerulean-H2Bv3, (negative control), Flamindo2 (positive control), and GtaC-mCherry (test). Because of the high numbers of fluorescent intensities present in each distribution (>62,000 pixels each), all of these tests returned very low *p*-values. However, the Kolmogorov–Smirnov test statistics themselves provide a quantitative measure of the differences between the distributions. With this in mind, we took the resultant four Kolmogorov-Smirnov test statistics per channel and performed Brown-Forsythe ANOVA and Dunnett's T3 multiple comparison testing between mCerulean-H2Bv3 and Flamindo2, and mCerulean-H2Bv3 and GtaC-mCherry in Prism. This testing demonstrated a significant difference between the negative control mCerulean-H2Bv3 and the positive control Flamindo2. It also demonstrated a significant difference between the negative control mCerulean-H2Bv3 and GtaC-mCherry. These findings suggest that the distributions of fluorescent intensities of GtaC-mCherry in cAMP-high versus cAMP-low nuclei differ more significantly than the same comparison performed on the mCerulean-H2Bv3 negative control.

### Plasmid, strain, and data availability

All GoldenBraid plasmids (Supplementary Table S2), associated maps and sequence files, and clonal *D. discoideum* strains (Supplementary Table S4) are available from the Dicty Stock Center (http://dictybase.org/StockCenter).

## RESULTS AND DISCUSSION

To adapt GoldenBraid for applications in *D. discoideum*, we generated plasmid backbones that include *D. discoideum* selectable markers for resistance to neomycin, hygromycin or blasticidin S. Because the selectable marker is present in the plasmid backbone from the start of the cloning process, these backbones accelerate cloning by uniformly reducing the number of assemblies necessary to generate transformable vectors, although the system's modularity allows integration of other selectable markers as needed. We also established a starter collection by generating a plasmid library of 99 elements that are ready for GoldenBraid assembly. We chose most of these elements for their broad usefulness and, where applicable, optimized the first few codons to promote protein expression (14). A selection of these parts is shown in Supplementary Table S1. To functionally validate each of these parts, we first placed them in their appropriate genetic contexts through constructing a collection of 86 assemblies. We then transformed each of these plasmids into *D. discoideum* and monitored the function of each part. For example, we tested in mixed populations of transformed cells the ability of the promoters to drive gene expression, the fluorescence of the fluorescent proteins (Supplementary Figure S1), and the drug resistance conferred by the drug-resistance genes.

The process of preparing elements for GoldenBraid cloning is called domestication. It begins with PCR amplification of an element with added nonhomologous primer overhangs on both ends to confer the grammatical identity. This amplicon is cloned into a domestication backbone (pUPD2) via a GoldenBraid reaction. Altogether we domesticated 10 promoters, 8 fluorescent proteins, 4 epitope tags, 2 linkers, 3 transcriptional terminators, 2 luciferases, 2 Cas9s, 1 guide RNA template, 13 protein CDSs, 3 drug-resistance CDSs and 20 barcodes (Supplementary Table S2). We generated a pair of loxP-flanked promoter (*coaA-loxP*) and terminator (*mhcA-loxP*) to facilitate the assembly of genetic elements that can be excised by Cre-mediated recombination (23). We also generated 156 GoldenBraid assemblies, most of which are of broad utility (Supplementary Table S2, Supplementary Protocols). Theoretically, the 99 genetic elements can be combined into several hundred transcriptional units, which can be further combined into single vectors to yield hundreds of thousands of assemblies of multiple transcriptional units (Figure 1B).

We quantified the efficiencies of 97 GoldenBraid reactions by determining the fraction of desired bacterial colonies in each transformation. While the efficiencies varied, all the reactions produced the desired transformants as defined by obtaining at least one clone that exhibited the correct pattern upon restriction enzyme fingerprinting of 2–4 plasmid minipreps from white bacterial colonies (Figure 1C), so the success rate was 100% in terms of obtaining the correct assembly. We tested the utility of GoldenBraid in *D. discoideum* in several ways. First, we expanded the fluorescent protein spectrum by cloning and validation of the blue fluorescent protein TagBFP (24) and the far-red fluorescent protein mIFP (25) (Figure 1D; Supplementary Figure S1A). Notably, we observed higher cell-to-cell variability in the level of TagBFP fluorescence as compared to other fluorescent proteins, despite isolating and imaging three individual clones. This finding may be explained by a cryptic property of our particular assembly, or could be a general feature of TagBFP expression in *D. discoideum*. TagBFP fluoresces on its own, but mIFP requires a biliverdin cofactor to fluoresce. Addition of biliverdin to the development buffer was sufficient for weak fluorescence throughout development. Addition of biliverdin to the agar on which the cells were developed increased the fluorescence significantly without compromising development (Supplementary Figure S2).

Next, we tested GoldenBraid's modularity and scalability. When *D. discoideum* cells starve, they aggregate into multicellular structures using chemotaxis toward pulses of extracellular cyclic AMP (cAMP) (26). Exposure to extracellular cAMP leads to cellular changes that include polarization, forward movement, increase in intracellular cAMP (26), and nucleoplasmic shuttling of the GtaC transcription factor (22). These events and the relationships between them have been documented separately. To visualize them together, we used a single assembly to express four fluorescent reporters in one *D. discoideum* strain (Figure 2A). To visualize intracellular cAMP we used Flamindo2, which fluoresces more intensely as cAMP levels decrease (27). To visualize cell polarity we used a fusion of the pleckstrin-homology (PH) domain to sfGFP, which localizes to the leading edge membrane of polarized cells upon cAMP stimulation (28). We fused mCherry to GtaC, the transcription factor which shuttles between the nucleus and cytoplasm every ∼6 min in response to cAMP waves (22). Finally, we fused the histone H2Bv3 with mCerulean to identify the position of the nuclei (29) and to facilitate the detection of single cells in aggregates. These protein fusions allowed us to visualize nuclei, areas of low versus high intracellular cAMP, cell polarization and nucleocytoplasmic shuttling of GtaC simultaneously in live cells during development. Figure 2B and Supplementary Video 1 show a population of cells, streaming from right to left. The cyan areas, with high Flamindo2 fluorescence, indicate low cAMP levels, and the intervening areas are consistent with high cAMP levels during the aggregation-stage pattern of cAMP waves (26) if the waves were moving from left to right. Cells with intense green edges are seen in areas of high cAMP and in immediately adjacent areas. These are the leading edges of polarized cells, indicated by PH-sfGFP, which occur as the cells encounter a cAMP wave (28). The red fluorescence of GtaC-mCherry is harder to visualize due to overexpression of the marker. We therefore identified the position of the nuclei using the mCerulean-H2Bv3 marker and quantified the intensity of the GtaC-mCherry and the Flamindo2 cAMP sensor in the same region in each cell. We then categorized each cell as exhibiting either high or low cAMP levels and tested the correlation with nuclear mCherry fluorescence (Figure 2C). The results of two independent experiments, each done in duplicates (Figure 2D), show that GtaC nuclear localization is significantly enriched in cells with low levels of intracellular cAMP, consistent with the finding that GtaC shuttles into the nucleus toward the end of the cAMP pulse (22). This analysis would be difficult to perform with standard cloning technology, but it was rather straightforward with GoldenBraid.

Additionally, we validated a GoldenBraid assembly of the photo-switchable fluorescent protein Dendra2 (30,31) (Supplementary Figure S1). We validated GoldenBraid iterations of several additional biosensors, including AbpC (32), which marks the rear of polarized cells (Supplementary Figure S1), the yellow cAMP sensor Flamindo2 (27), the red-channel cAMP sensor Pink Flamindo (33) (Supplementary Videos 2 and 3), and the HA epitope-tag (Supplementary Figure S1). Finally, we domesticated all of the necessary parts to effect CRISPR–Cas9 activity in *D. discoideum* (6), assembled them to generate the vector pDGB_A2_CRISPR1, and validated editing by inactivating the genes *acaA* and *rapgapB* (Supplementary Figure S3).

We conclude that GoldenBraid is a standardized, rapid, and robust cloning technology for *D. discoideum* that allows community-wide, shared development of reagents for novel synthetic biological applications. We propose that new vectors made with GoldenBraid should be deposited in the Dicty Stock Center (2) to facilitate rapid exchange of reagents and enhanced development of synthetic biology tools.

## Supplementary Material

gkaa185_Supplemental_Files
