# Supplementary material for: A GoldenBraid cloning system for synthetic biology in social amoebae
Source: Nucleic Acids Res. 2020 Mar 30;48(8):4139–46. doi: 10.1093/nar/gkaa185 (PMC7192589; doi:10.1093/nar/gkaa185)
Supplement: gkaa185_Supplemental_Files [file gkaa185_supplemental_files.zip › Kundert Supplementary Information revised.docx]

Supplementary information


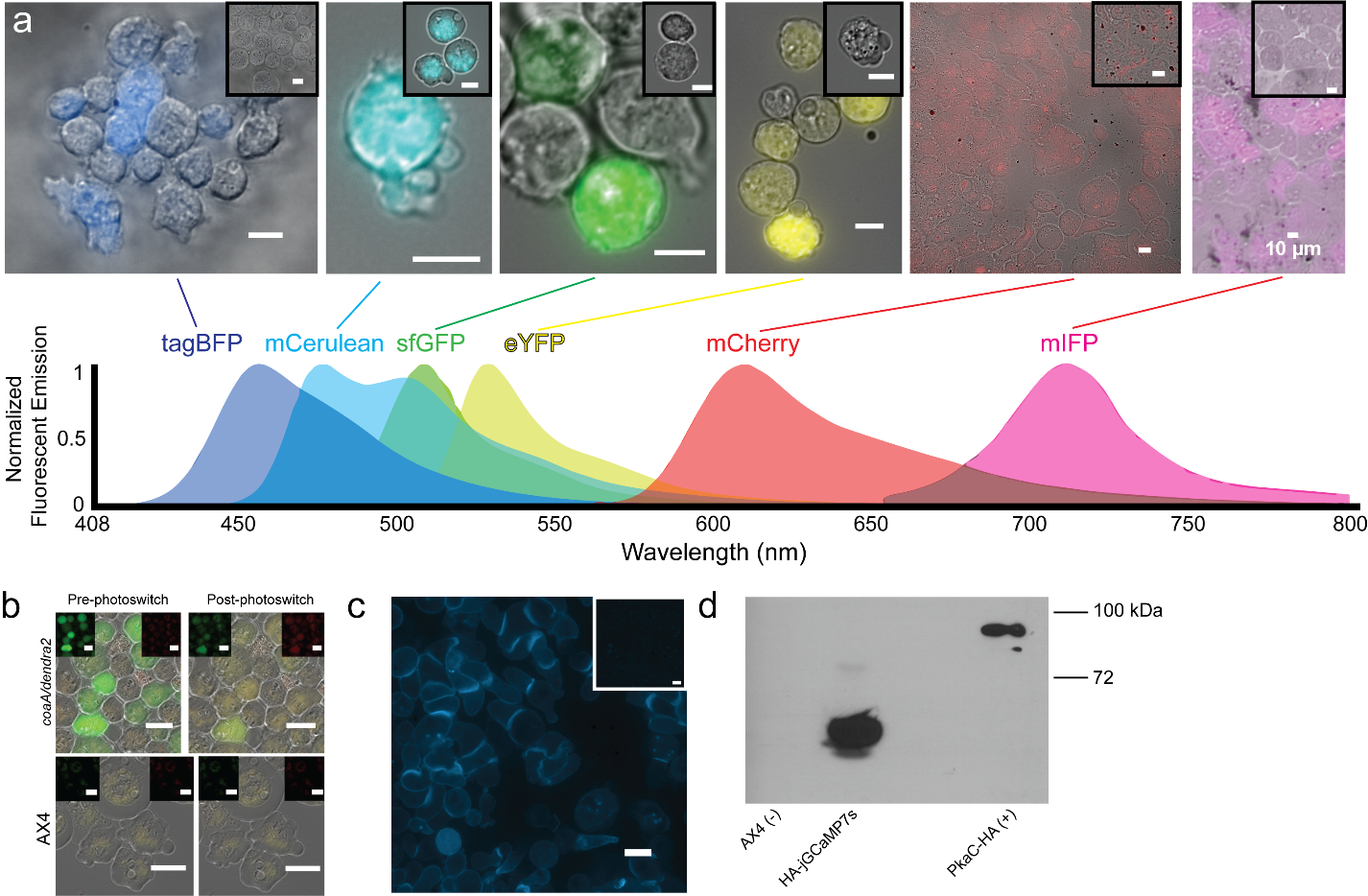


Supplementary Fig. 1 | Validation of selected GoldenBraid parts in *D. discoideum*. **a**, Representative images of non-clonal (except tagBFP and mCherry) populations of transformants expressing one of a palette of fluorescent proteins, overlayed with the differential interference contrast (DIC) channel to show cellular morphology. Insets are representative overlays of untransformed, wild-type AX4 cells as negative controls. mCherry is fused to GtaC. Levels of autofluorescence in the CFP channel are high in vesicles inside the pictured AX4 cells, but expression of mCerulean is still apparent because of the pictured cell’s uniform fluorescence throughout its cytoplasm. Bar: 10 µm. The graph shows the emission spectra of the respective proteins (adapted from fpbase.org). **b**, Representative images demonstrate expression and photoconversion from green to red of the Dendra2 fluorescent protein. Dendra2 photoconversion is a tractable method to mark cells for tracking applications. Left insets are the GFP channel; right insets are the RFP channel. Top row: transformed cells; bottom row: untransformed AX4 control. Bar: 20 µm. **c**, Representative image demonstrating expression of a mCerulean-AbpC (a.k.a. Abp120) fluorescent protein fusion. AbpC marks cell cortices. Inset: untransformed AX4 cells. Bar: 20 µm. **d**, Validation of the HA affinity tag by Western blot analysis of cell lysates from *Dictyostelium* strains that express the indicated tagged proteins. Expected protein size: HA-jGCaMP7s = 48.5 kDa, PkaC-HA = 75 kDa.


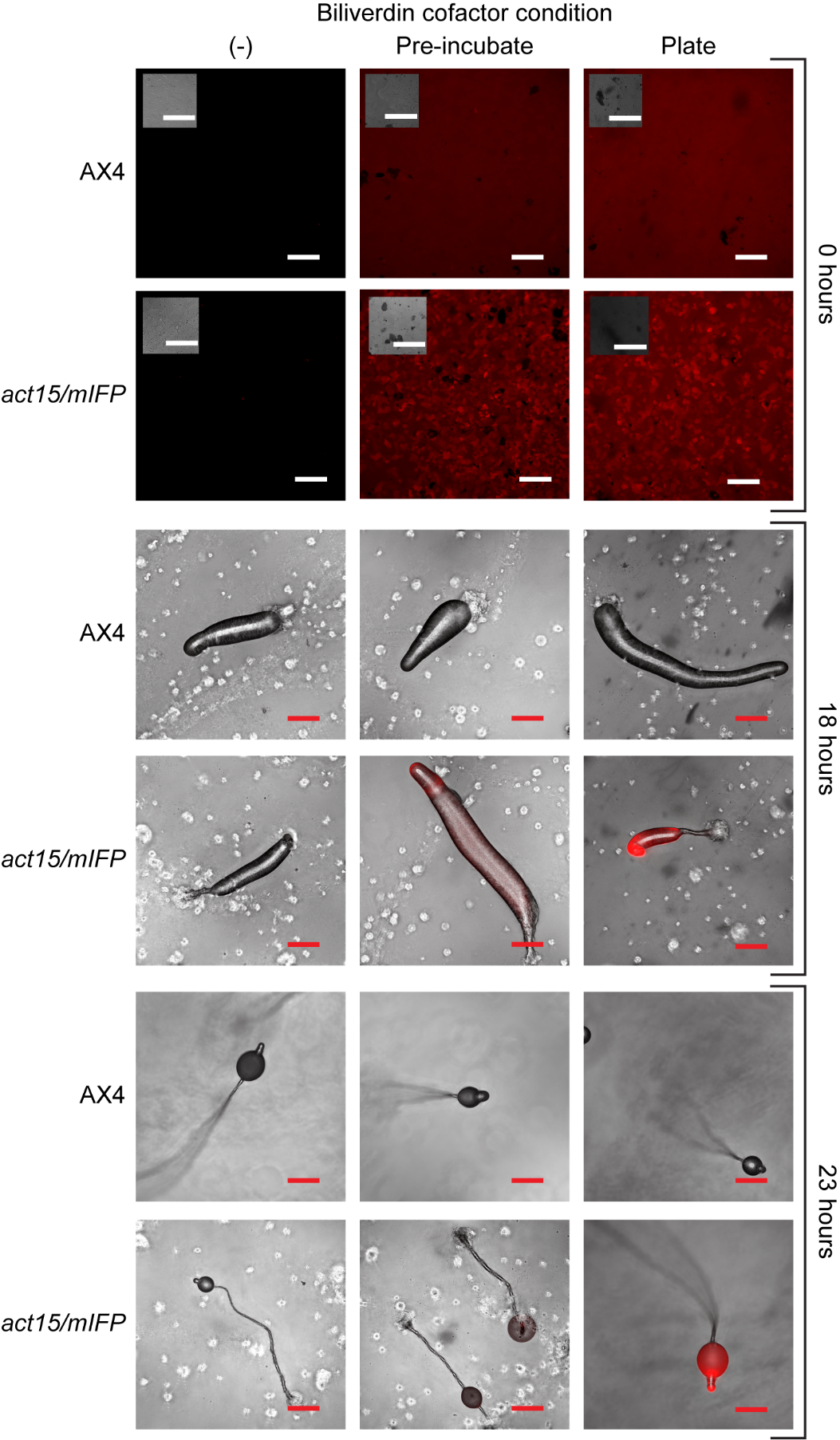


Supplementary Fig. 2 | Expression of the fluorescent protein mIFP in *Dictyostelium* results in a far-red fluorescent signal in the presence of exogenous biliverdin. A clone of *act15/mIFP* cells and wild-type AX4 cells (negative control) were imaged throughout multicellular development on KK2 + 2% Noble agar plates. At 0 hours, the presence of 50 µg/mL biliverdin cofactor caused low-level background fluorescence, but mIFP-expressing cells were visible above this background. Insets (DIC images) show that cells are present in each image. At 18 and 23 hours of development, fluorescent developmental structures are visible on far-red and DIC overlays. Higher levels of fluorescence are present when 50 µg/mL biliverdin was added to the agar, as opposed to the condition in which cells were pre-incubated in liquid PDF buffer + 50 µg/mL biliverdin at 0 hours development. These higher levels likely result from the continued presence of biliverdin in the agar throughout development (middle and bottom). Scale bars are all 100 µm.

***
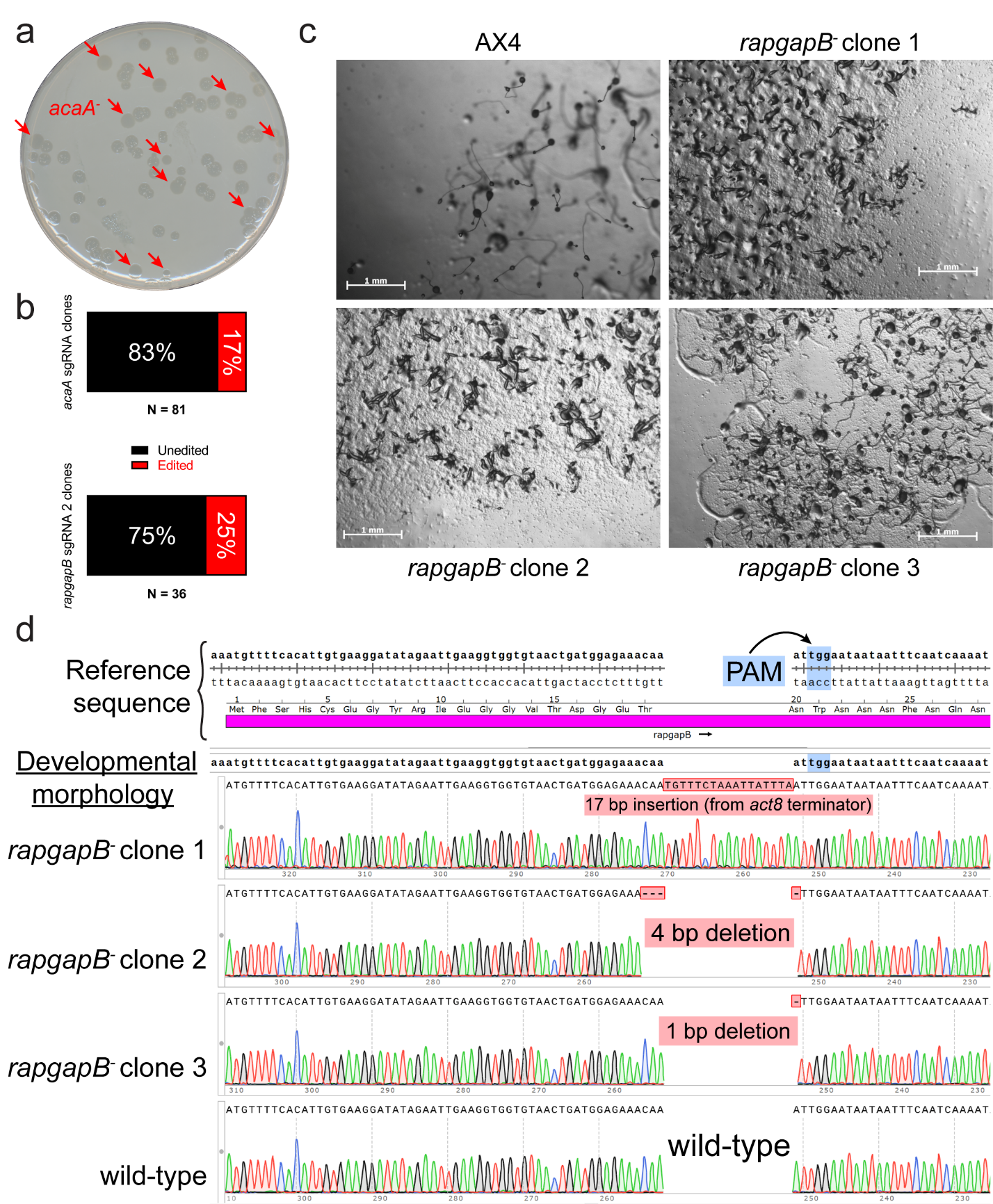
***

Supplementary Fig. 3 | CRISPR editing using the GoldenBraid vector pDGB_A2_CRISPR1 generates *D. discoideum* knockout strains. **a**, An sgRNA targeting the *acaA* gene was cloned into this vector in a GoldenBraid-like reaction using *Bbs*I restriction enzyme. The vector was then transiently transformed into AX4 cells. Individual transformed clones were isolated as plaques on a 10-cm plate with *Klebsiella pneumoniae* food bacteria. Edited clones with loss-of-function alleles of *acaA* (red arrows) cannot aggregate, which confirms successful CRISPR editing. **b**, Editing efficiencies using the pDGB_A2_CRISPR1; red – edited, black – non-edited. **c**, Similar to a previously published knockout strain, CRISPR-edited *rapgapB^–^* clones exhibit aberrant development, including formation of multi-tipped mounds and few, small fruiting bodies. **d**, Sanger sequencing traces of the three pictured *rapgapB^–^* clones demonstrate successful CRISPR editing that generated a short insertion as well as 1- and 4-bp deletions.


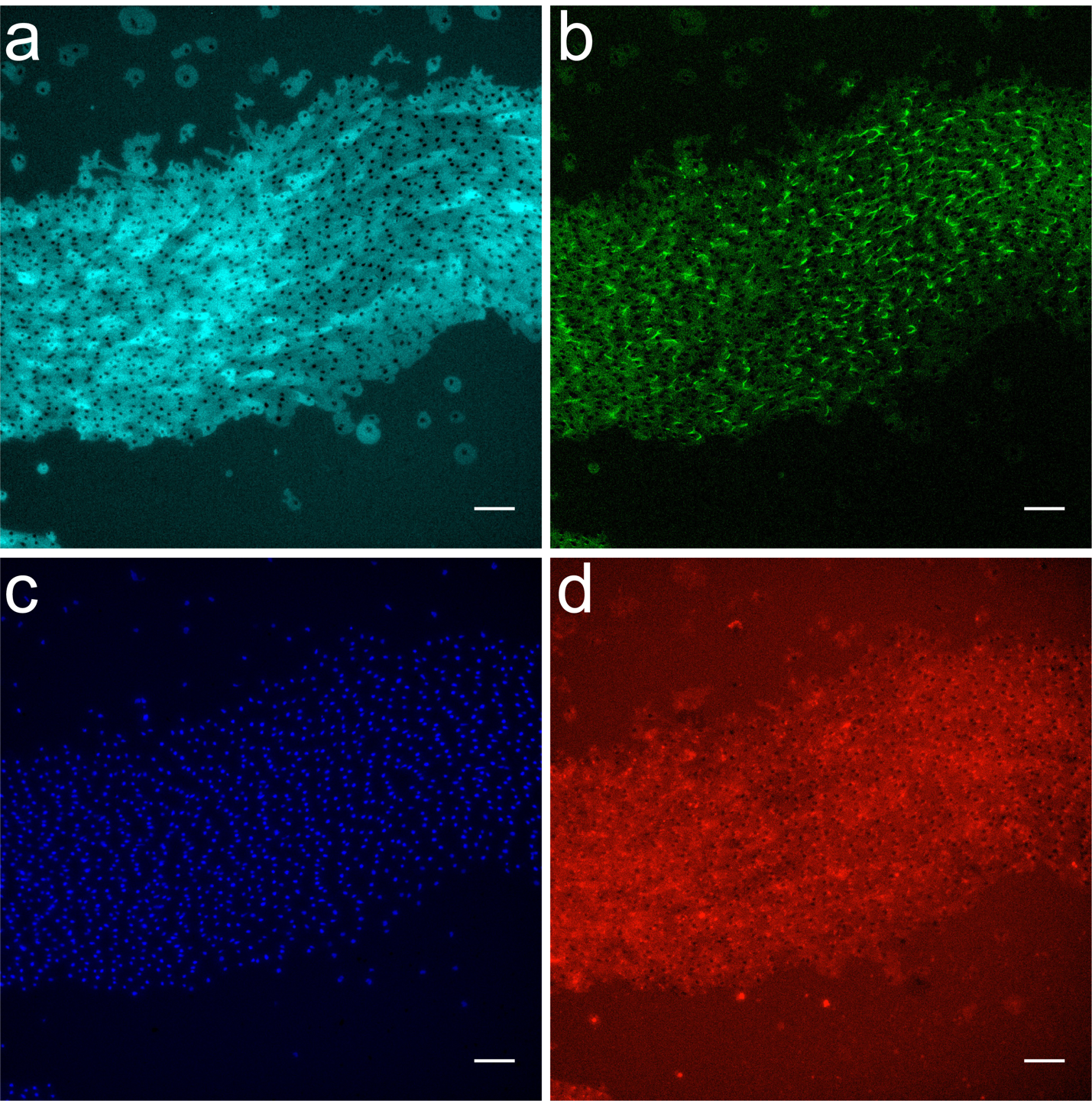


Supplementary Fig. 4 | Separate channel images of the picture shown in Figure 2.

The four panels show monochromatic images of the picture shown in Figure 2. Scale bars are each 50 µm. a, Flamindo2 (Citrine). **b**, PH-sfGFP. **c**, mCerulean-H2Bv3. **d**, GtaC-mCherry.

**Supplementary Video 1 | Simultaneous visualization of intracellular cAMP, leading edges, nuclei and nucleocytoplasmic shuttling of GtaC in live, developing amoebae.** Composite video of Flamindo2 cAMP sensor (pseudocolored cyan), H2Bv3-mCerulean nuclear marker (pseudocolored blue), PH-sfGFP leading edge marker (pseudocolored green), and GtaC-mCherry transcription factor (pseudocolored red). Cells are migrating from right to left, while cAMP waves are propagating from left to right. Areas of low Flamindo2 fluorescence have higher intracellular cAMP levels, while areas of high Flamindo2 fluorescence have lower intracellular cAMP levels. Cells were developed under agar for 8 hours prior to imaging. Images were taken every 1 minute for 8 minutes. Spectral un-mixing feature was performed in NIS Elements software to compensate for the spectral overlap between mCerulean and sfGFP. Metadata are provided in Supplementary Table 6.

**Supplementary Video 2 | Validation of a GoldenBraid vector expressing the single-channel, intracellular cAMP sensor Flamindo2 in live, developing amoebae.** *coaA/flamindo2* amoebae were developed for 7 hours under agar. Images were taken using the YFP channel (excitation 514 nm, emission 535 nm), as Flamindo2 employs a modified Citrine for its fluorescent readout. Flamindo2 fluorescent intensity increases as intracellular cAMP levels decrease^17^. Images were captured every 30 seconds for 15 minutes with little to no evidence of accumulating phototoxicity. The “Red Fire” LUT preset was applied to the YFP channel in NIS Elements software to accentuate changes in fluorescence intensity over time. Metadata are provided in Supplementary Table 6.

**Supplementary Video 3 | Validation of a GoldenBraid vector expressing the single-channel, intracellular cAMP sensor Pink Flamindo in live, developing amoebae.** *coaA/pinkflamindo* amoebae were developed for 7 hours under agar. Images were taken using the mCherry channel (excitation 555 nm, emission 620 nm), as Pink Flamindo employs a modified mApple for its fluorescent readout. Pink Flamindo fluorescent intensity increases as intracellular cAMP levels increase ^18^. Images were captured every 1 minute for 15 minutes with little to no evidence of accumulating phototoxicity, although some decay of the fluorescent signal occurred. The “Red Fire” LUT preset was applied to the mCherry channel in NIS Elements software to accentuate changes in fluorescence intensity over time. Metadata are provided in Supplementary Table 6.

Supplementary Table 1 | GoldenBraid elements available for use in *D. discoideum*

| **Part class** | **Promo-ter** | **N-terminal Tag** | | **5' Linker** | **CDS** | **3' Linker** | **C-terminal Tag** | **Terminator** | **Bar-code** |
| --- | --- | --- | --- | --- | --- | --- | --- | --- | --- |
| **5' gram-mar** | GGAG | AATG | | AGTG-GC | AATG or TAGC | TCGG-GC | CTCG | GCTT | CAGT |
| **3' gram-mar** | AATG | AGTG | | GGTA-GC | TCGG or GCTT | GGCT-CG | GCTT | CAGT or CGCT | CGCT |
| **Parts** | *actin6* | *mNeon*  *-Green* | | GGGSx4 | *mNeonGreen* | GGGSx4 | *mNeon*  *-Green* | *actin8* | x 20 |
|  | *actin15* | *sfGFP* | | FL | *sfGFP* | FL | *sfGFP* | *mhcA* |  |
|  | *coaA* | *mCherry* | |  | *mCherry* |  | *mCherry* | *mhcA-loxP* |  |
|  | *loxP-coaA* | *tagBFP* | |  | *tagBFP* |  | *tagBFP* |  |  |
|  | *ecmA* | *mIFP* | |  | *mIFP* |  | *mIFP* |  |  |
|  |  | *eYFP* | |  | *eYFP* |  | *eYFP* |  |  |
|  |  | *mCerule-an* | |  | *mCerule-an* |  | *mCerule-an* |  |  |
|  |  | *dendra2* | |  | *dendra2* |  | *dendra2* |  |  |
|  |  | *GST* | |  | *renilla* |  | *GST* |  |  |
|  |  | *HA* | |  | *Firefly lucif-erase* |  | *HA* |  |  |
|  |  | *7xHis* | |  | *Cas9* |  | *Myc* |  |  |
|  |  |  | |  | *Hypa-*  *Cas9* |  | *7xHis* |  |  |
|  | Validated in *Dictyostelium* | | |  | Ile tRNA::  sgRNA template |  |  |  |  |
|  | Sanger sequence-confirmed, but not validated in *Dictyostelium* | | |  | *ralGDS* |  |  |  |  |
|  |  |  | |  | *flamindo-2* |  |  |  |  |
|  |  |  | |  | *pink flamindo* |  |  |  |  |
|  |  |  | |  | *abpC* |  |  |  |  |
|  |  |  | |  | *H2Bv3* |  |  |  |  |
|  |  | |  |  | *PH* |  |  |  |  |
|  |  |  | |  | *gtaC* |  |  |  |  |
|  |  |  | |  | *BSR* |  |  |  |  |
|  |  |  | |  | *NeoR* |  |  |  |  |
|  |  |  | |  | *HygR* |  |  |  |  |

Supplementary Table 2 | Description of GoldenBraid plasmids.

| **Plasmid Name** | **Description** |
| --- | --- |
| pDGB_α1 | Bacterial abxR^a^: kanR; Dicty abxR: None; Using *Bsa*I, this backbone can accept one or more inserts liberated from domestication- or Ω-level backbones with the most-5' grammar as 5'-GGAG and the most-3' grammar as CGCT-3'. Using *Bsm*BI, this backbone can provide its insert with the grammar 5'-GGAG and GTCA-3’ to be cloned into an Ω-level backbone. |
| pDGB_α1B | Bacterial abxR: kanR; Dicty abxR: bsR; Using *Bsa*I, this backbone can accept one or more inserts liberated from domestication- or Ω-level backbones with the most-5' grammar as 5'-GGAG and the most-3' grammar as CGCT-3'. Using *Bsm*BI, this backbone can provide its insert with the grammar 5'-GGAG and GTCA-3’ to be cloned into an Ω-level backbone. |
| pDGB_α1H | Bacterial abxR: kanR; Dicty abxR: hygR; Using *Bsa*I, this backbone can accept one or more inserts liberated from domestication- or Ω-level backbones with the most-5' grammar as 5'-GGAG and the most-3' grammar as CGCT-3'. Using *Bsm*BI, this backbone can provide its insert with the grammar 5'-GGAG and GTCA-3’ to be cloned into an Ω-level backbone. |
| pDGB_α1N | Bacterial abxR: kanR; Dicty abxR: neoR; Using *Bsa*I, this backbone can accept one or more inserts liberated from domestication- or Ω-level backbones with the most-5' grammar as 5'-GGAG and the most-3' grammar as CGCT-3'. Using *Bsm*BI, this backbone can provide its insert with the grammar 5'-GGAG and GTCA-3’ to be cloned into an Ω-level backbone. |
| pDGB_α2 | Bacterial abxR: kanR; Dicty abxR: None; Using *Bsa*I, this backbone can accept one or more inserts liberated from domestication- or Ω-level backbones with the most-5' grammar as 5'-GGAG and the most-3' grammar as CGCT-3'. Using *Bsm*BI, this backbone can provide its insert with the grammar 5'-GTCA and CGCT-3' to be cloned into an Ω-level backbone. |
| pDGB_α2B | Bacterial abxR: kanR; Dicty abxR: bsR; Using *Bsa*I, this backbone can accept one or more inserts liberated from domestication- or Ω-level backbones with the most-5' grammar as 5'-GGAG and the most-3' grammar as CGCT-3'. Using *Bsm*BI, this backbone can provide its insert with the grammar 5'-GTCA and CGCT-3' to be cloned into an Ω-level backbone. |
| pDGB_α2H | Bacterial abxR: kanR; Dicty abxR: hygR; Using *Bsa*I, this backbone can accept one or more inserts liberated from domestication- or Ω-level backbones with the most-5' grammar as 5'-GGAG and the most-3' grammar as CGCT-3'. Using *Bsm*BI, this backbone can provide its insert with the grammar 5'-GTCA and CGCT-3' to be cloned into an Ω-level backbone. |
| pDGB_α2N | Bacterial abxR: kanR; Dicty abxR: neoR; Using *Bsa*I, this backbone can accept one or more inserts liberated from domestication- or Ω-level backbones with the most-5' grammar as 5'-GGAG and the most-3' grammar as CGCT-3'. Using *Bsm*BI, this backbone can provide its insert with the grammar 5'-GTCA and CGCT-3' to be cloned into an Ω-level backbone. |
| pDGB_αB | Bacterial abxR: kanR; Dicty abxR: None; Using *Bsa*I, this backbone can accept one or more inserts liberated from domestication- or Ω-level backbones with the most-5' grammar as 5'-GGAG and the most-3' grammar as CGCT-3'. Using *Bsm*BI, this backbone can provide its insert with the grammar 5'-GTCA and AATG-3' to be cloned into an Ω-level backbone. |
| pDGB_αBB | Bacterial abxR: kanR; Dicty abxR: bsR; Using *Bsa*I, this backbone can accept one or more inserts liberated from domestication- or Ω-level backbones with the most-5' grammar as 5'-GGAG and the most-3' grammar as CGCT-3'. Using *Bsm*BI, this backbone can provide its insert with the grammar 5'-GTCA and AATG-3' to be cloned into an Ω-level backbone. |
| pDGB_αBH | Bacterial abxR: kanR; Dicty abxR: hygR; Using *Bsa*I, this backbone can accept one or more inserts liberated from domestication- or Ω-level backbones with the most-5' grammar as 5'-GGAG and the most-3' grammar as CGCT-3'. Using *Bsm*BI, this backbone can provide its insert with the grammar 5'-GTCA and AATG-3' to be cloned into an Ω-level backbone. |
| pDGB_αBN | Bacterial abxR: kanR; Dicty abxR: neoR; Using *Bsa*I, this backbone can accept one or more inserts liberated from domestication- or Ω-level backbones with the most-5' grammar as 5'-GGAG and the most-3' grammar as CGCT-3'. Using *Bsm*BI, this backbone can provide its insert with the grammar 5'-GTCA and AATG-3' to be cloned into an Ω-level backbone. |
| pDGB_αC | Bacterial abxR: kanR; Dicty abxR: None; Using *Bsa*I, this backbone can accept one or more inserts liberated from domestication- or Ω-level backbones with the most-5' grammar as 5'-GGAG and the most-3' grammar as CGCT-3'. Using *Bsm*BI, this backbone can provide its insert with the grammar 5'-AATG and GCTT-3' to be cloned into an Ω-level backbone. |
| pDGB_αCH | Bacterial abxR: kanR; Dicty abxR: hygR; Using *Bsa*I, this backbone can accept one or more inserts liberated from domestication- or Ω-level backbones with the most-5' grammar as 5'-GGAG and the most-3' grammar as CGCT-3'. Using *Bsm*BI, this backbone can provide its insert with the grammar 5'-AATG and GCTT-3' to be cloned into an Ω-level backbone. |
| pDGB_αCN | Bacterial abxR: kanR; Dicty abxR: neoR; Using *Bsa*I, this backbone can accept one or more inserts liberated from domestication- or Ω-level backbones with the most-5' grammar as 5'-GGAG and the most-3' grammar as CGCT-3'. Using *Bsm*BI, this backbone can provide its insert with the grammar 5'-AATG and GCTT-3' to be cloned into an Ω-level backbone. |
| pDGB_αD | Bacterial abxR: kanR; Dicty abxR: None; Using *Bsa*I, this backbone can accept one or more inserts liberated from domestication- or Ω-level backbones with the most-5' grammar as 5'-GGAG and the most-3' grammar as CGCT-3'. Using *Bsm*BI, this backbone can provide its insert with the grammar 5'-GCTT and TGGC-3' to be cloned into an Ω-level backbone. |
| pDGB_αDB | Bacterial abxR: kanR; Dicty abxR: bsR; Using *Bsa*I, this backbone can accept one or more inserts liberated from domestication- or Ω-level backbones with the most-5' grammar as 5'-GGAG and the most-3' grammar as CGCT-3'. Using *Bsm*BI, this backbone can provide its insert with the grammar 5'-GCTT and TGGC-3' to be cloned into an Ω-level backbone. |
| pDGB_αDH | Bacterial abxR: kanR; Dicty abxR: hygR; Using *Bsa*I, this backbone can accept one or more inserts liberated from domestication- or Ω-level backbones with the most-5' grammar as 5'-GGAG and the most-3' grammar as CGCT-3'. Using *Bsm*BI, this backbone can provide its insert with the grammar 5'-GCTT and TGGC-3' to be cloned into an Ω-level backbone. |
| pDGB_αDN | Bacterial abxR: kanR; Dicty abxR: neoR; Using *Bsa*I, this backbone can accept one or more inserts liberated from domestication- or Ω-level backbones with the most-5' grammar as 5'-GGAG and the most-3' grammar as CGCT-3'. Using *Bsm*BI, this backbone can provide its insert with the grammar 5'-GCTT and TGGC-3' to be cloned into an Ω-level backbone. |
| pDGB_αE | Bacterial abxR: kanR; Dicty abxR: None; Using *Bsa*I, this backbone can accept one or more inserts liberated from domestication- or Ω-level backbones with the most-5' grammar as 5'-GGAG and the most-3' grammar as CGCT-3'. Using *Bsm*BI, this backbone can provide its insert with the grammar 5'-TGGC and CGCT-3' to be cloned into an Ω-level backbone. |
| pDGB_αEB | Bacterial abxR: kanR; Dicty abxR: bsR; Using *Bsa*I, this backbone can accept one or more inserts liberated from domestication- or Ω-level backbones with the most-5' grammar as 5'-GGAG and the most-3' grammar as CGCT-3'. Using *Bsm*BI, this backbone can provide its insert with the grammar 5'-TGGC and CGCT-3' to be cloned into an Ω-level backbone. |
| pDGB_αEN | Bacterial abxR: kanR; Dicty abxR: neoR; Using *Bsa*I, this backbone can accept one or more inserts liberated from domestication- or Ω-level backbones with the most-5' grammar as 5'-GGAG and the most-3' grammar as CGCT-3'. Using *Bsm*BI, this backbone can provide its insert with the grammar 5'-TGGC and CGCT-3' to be cloned into an Ω-level backbone. |
| pDGB_Ω1 | Bacterial abxR: chlorR; Dicty abxR: None; Using *Bsm*BI, this backbone can accept inserts liberated from α-level backbones with the most-5' grammar as 5'-GGAG and the most-3' grammar as CGCT-3'. Using *Bsa*I, this backbone can provide its insert with the grammar 5'-GGAG and GTCA-3’ to be cloned into an α-level backbone. |
| pDGB_Ω1B | Bacterial abxR: chlorR; Dicty abxR:bsR; Using *Bsm*BI, this backbone can accept inserts liberated from α-level backbones with the most-5' grammar as 5'-GGAG and the most-3' grammar as CGCT-3'. Using *Bsa*I, this backbone can provide its insert with the grammar 5'-GGAG and GTCA-3’ to be cloned into an α-level backbone. |
| pDGB_Ω1H | Bacterial abxR: chlorR; Dicty abxR: hygR; Using *Bsm*BI, this backbone can accept inserts liberated from α-level backbones with the most-5' grammar as 5'-GGAG and the most-3' grammar as CGCT-3'. Using *Bsa*I, this backbone can provide its insert with the grammar 5'-GGAG and GTCA-3’ to be cloned into an α-level backbone. |
| pDGB_Ω1N | Bacterial abxR: chlorR; Dicty abxR: neoR; Using *Bsm*BI, this backbone can accept inserts liberated from α-level backbones with the most-5' grammar as 5'-GGAG and the most-3' grammar as CGCT-3'. Using *Bsa*I, this backbone can provide its insert with the grammar 5'-GGAG and GTCA-3’ to be cloned into an α-level backbone. |
| pDGB_Ω2 | Bacterial abxR: chlorR; Dicty abxR: None; Using *Bsm*BI, this backbone can accept inserts liberated from α-level backbones with the most-5' grammar as 5'-GGAG and the most-3' grammar as CGCT-3'. Using *Bsa*I, this backbone can provide its insert with the grammar 5'-GTCA and CGCT-3' to be cloned into an α-level backbone. |
| pDGB_Ω2B | Bacterial abxR: chlorR; Dicty abxR: bsR; Using *Bsm*BI, this backbone can accept inserts liberated from α-level backbones with the most-5' grammar as 5'-GGAG and the most-3' grammar as CGCT-3'. Using *Bsa*I, this backbone can provide its insert with the grammar 5'-GTCA and CGCT-3' to be cloned into an α-level backbone. |
| pDGB_Ω2H | Bacterial abxR: chlorR; Dicty abxR: hygR; Using *Bsm*BI, this backbone can accept inserts liberated from α-level backbones with the most-5' grammar as 5'-GGAG and the most-3' grammar as CGCT-3'. Using *Bsa*I, this backbone can provide its insert with the grammar 5'-GTCA and CGCT-3' to be cloned into an α-level backbone. |
| pDGB_Ω2N | Bacterial abxR: chlorR; Dicty abxR: neoR; Using *Bsm*BI, this backbone can accept inserts liberated from α-level backbones with the most-5' grammar as 5'-GGAG and the most-3' grammar as CGCT-3'. Using *Bsa*I, this backbone can provide its insert with the grammar 5'-GTCA and CGCT-3' to be cloned into an α-level backbone. |
| pUPD2 | Bacterial abxR: ampR; Dicty abxR: None; Domestication-level backbone into which individual GB parts can be cloned using *Bsm*BI with the grammar 5'-CTCG and CTCG-3'. Using *Bsa*I, individual parts can be liberated with custom 5'- and 3'- grammatical sequences introduced by non-homologous primer overhangs during PCR amplification. |
| pUPD2_7xHis_AATG_AGTG | Bacterial abxR: ampR; Dicty abxR: None; N-terminal affinity tag to be fused with a linker to a CDS of interest; this GB part is not validated in Dicty; template source: dsDNA oligo |
| pUPD2_7xHis_CTCG_GCTT | Bacterial abxR: ampR; Dicty abxR: None; C-terminal affinity tag to be fused with a linker to a CDS of interest; this GB part is not validated in Dicty; template source: dsDNA oligo |
| pUPD2_abpC_AATG_TCGG | Bacterial abxR: ampR; Dicty abxR: None; cell cortex marker, localizes to the rear of polarized cells; designed as CDS to be fused to a C-terminal linker and affinity tag or fluorescent marker; this GB part is validated in Dicty by microscopy; template source: Dicty gDNA or gDNA library |
| pUPD2_actin6p_GGAG_AATG | Bacterial abxR: ampR; Dicty abxR: None; overexpression promoter; this GB part is not validated in Dicty; template source: Dicty gDNA or gDNA library |
| pUPD2_actin8t_GCTT_CGCT | Bacterial abxR: ampR; Dicty abxR: None; standard Dictyostelium terminator; this GB part is validated in Dicty by successful transformation; template source: pLPBLP |
| pUPD2_actin15p_GGAG_AATG | Bacterial abxR: ampR; Dicty abxR: None; overexpression promoter; this GB part is validated in Dicty by microscopy and Western blot; template source: pLPBLP |
| pUPD2_aurKp_GGAG_AATG | Bacterial abxR: ampR; Dicty abxR: None; putative light-inducible promoter; this GB part is validated in Dicty by microscopy; template source: Dicty gDNA or gDNA library |
| pUPD2_Barcode1_CAGT_CGCT | Bacterial abxR: ampR; Dicty abxR: None; 40-bp barcode to be located 3' of the terminator in a given assembly; a representative of this GB part is validated in Dicty by successful transformation; template source: pool of dsDNA oligos |
| pUPD2_Barcode2_CAGT_CGCT | Bacterial abxR: ampR; Dicty abxR: None; 40-bp barcode to be located 3' of the terminator in a given assembly; a representative of this GB part is validated in Dicty by successful transformation; template source: pool of dsDNA oligos |
| pUPD2_Barcode3_CAGT_CGCT | Bacterial abxR: ampR; Dicty abxR: None; 40-bp barcode to be located 3' of the terminator in a given assembly; a representative of this GB part is validated in Dicty by successful transformation; template source: pool of dsDNA oligos |
| pUPD2_Barcode4_CAGT_CGCT | Bacterial abxR: ampR; Dicty abxR: None; 40-bp barcode to be located 3' of the terminator in a given assembly; a representative of this GB part is validated in Dicty by successful transformation; template source: pool of dsDNA oligos |
| pUPD2_Barcode5_CAGT_CGCT | Bacterial abxR: ampR; Dicty abxR: None; 40-bp barcode to be located 3' of the terminator in a given assembly; a representative of this GB part is validated in Dicty by successful transformation; template source: pool of dsDNA oligos |
| pUPD2_Barcode6_CAGT_CGCT | Bacterial abxR: ampR; Dicty abxR: None; 40-bp barcode to be located 3' of the terminator in a given assembly; a representative of this GB part is validated in Dicty by successful transformation; template source: pool of dsDNA oligos |
| pUPD2_Barcode7_CAGT_CGCT | Bacterial abxR: ampR; Dicty abxR: None; 40-bp barcode to be located 3' of the terminator in a given assembly; a representative of this GB part is validated in Dicty by successful transformation; contains a *Bsa*I site within the barcode that could affect assembly; template source: pool of dsDNA oligos |
| pUPD2_Barcode8_CAGT_CGCT | Bacterial abxR: ampR; Dicty abxR: None; 40-bp barcode to be located 3' of the terminator in a given assembly; a representative of this GB part is validated in Dicty by successful transformation; template source: pool of dsDNA oligos |
| pUPD2_Barcode9_CAGT_CGCT | Bacterial abxR: ampR; Dicty abxR: None; 40-bp barcode to be located 3' of the terminator in a given assembly; a representative of this GB part is validated in Dicty by successful transformation; template source: pool of dsDNA oligos |
| pUPD2_Barcode10_CAGT_CGCT | Bacterial abxR: ampR; Dicty abxR: None; 40-bp barcode to be located 3' of the terminator in a given assembly; a representative of this GB part is validated in Dicty by successful transformation; template source: pool of dsDNA oligos |
| pUPD2_Barcode11_CAGT_CGCT | Bacterial abxR: ampR; Dicty abxR: None; 40-bp barcode to be located 3' of the terminator in a given assembly; a representative of this GB part is validated in Dicty by successful transformation; template source: pool of dsDNA oligos |
| pUPD2_Barcode12_CAGT_CGCT | Bacterial abxR: ampR; Dicty abxR: None; 40-bp barcode to be located 3' of the terminator in a given assembly; a representative of this GB part is validated in Dicty by successful transformation; template source: pool of dsDNA oligos |
| pUPD2_Barcode13_CAGT_CGCT | Bacterial abxR: ampR; Dicty abxR: None; 40-bp barcode to be located 3' of the terminator in a given assembly; a representative of this GB part is validated in Dicty by successful transformation; template source: pool of dsDNA oligos |
| pUPD2_Barcode14_CAGT_CGCT | Bacterial abxR: ampR; Dicty abxR: None; 40-bp barcode to be located 3' of the terminator in a given assembly; a representative of this GB part is validated in Dicty by successful transformation; template source: pool of dsDNA oligos |
| pUPD2_Barcode15_CAGT_CGCT | Bacterial abxR: ampR; Dicty abxR: None; 40-bp barcode to be located 3' of the terminator in a given assembly; a representative of this GB part is validated in Dicty by successful transformation; template source: pool of dsDNA oligos |
| pUPD2_Barcode16_CAGT_CGCT | Bacterial abxR: ampR; Dicty abxR: None; 40-bp barcode to be located 3' of the terminator in a given assembly; a representative of this GB part is validated in Dicty by successful transformation; template source: pool of dsDNA oligos |
| pUPD2_Barcode17_CAGT_CGCT | Bacterial abxR: ampR; Dicty abxR: None; 40-bp barcode to be located 3' of the terminator in a given assembly; a representative of this GB part is validated in Dicty by successful transformation; template source: pool of dsDNA oligos |
| pUPD2_Barcode18_CAGT_CGCT | Bacterial abxR: ampR; Dicty abxR: None; 40-bp barcode to be located 3' of the terminator in a given assembly; a representative of this GB part is validated in Dicty by successful transformation; template source: pool of dsDNA oligos |
| pUPD2_Barcode19_CAGT_CGCT | Bacterial abxR: ampR; Dicty abxR: None; 40-bp barcode to be located 3' of the terminator in a given assembly; a representative of this GB part is validated in Dicty by successful transformation; template source: pool of dsDNA oligos |
| pUPD2_Barcode20_CAGT_CGCT | Bacterial abxR: ampR; Dicty abxR: None; 40-bp barcode to be located 3' of the terminator in a given assembly; a representative of this GB part is validated in Dicty by successful transformation; template source: pool of dsDNA oligos |
| pUPD2_bsR_AATG_GCTT | Bacterial abxR: ampR; Dicty abxR: None; bsR as a standalone ORF; this GB part is validad in Dicty by successful transformation and drug selection; template source: pLPBLP |
| pUPD2_coaAp_GGAG_AATG | Bacterial abxR: ampR; Dicty abxR: None; overexpression promoter; this GB part is validated in Dicty by microscopy; template source: Dicty gDNA or gDNA library |
| pUPD2_ctsZ_AATG_TCGG | Bacterial abxR: ampR; Dicty abxR: None; putative secreted antimicrobial designed as CDS to be fused to a C-terminal linker and affinity tag or fluorescent marker; this GB part is not validated in Dicty; template source: Dicty gDNA or gDNA library |
| pUPD2_DDB_G0278295_AATG_TCGG | Bacterial abxR: ampR; Dicty abxR: None; putative secreted antimicrobial designed as CDS to be fused to a C-terminal linker and affinity tag or fluorescent marker; this GB part is expressed by Western blot but not functionally characterized in Dicty; template source: Dicty gDNA or gDNA library |
| pUPD2_DDB_G0288429p_GGAG_AATG | Bacterial abxR: ampR; Dicty abxR: None; light-inducible promoter; this GB part is validated in Dicty by microscopy; template source: Dicty gDNA or gDNA library |
| pUPD2_DDB_G0289731_AATG_TCGG | Bacterial abxR: ampR; Dicty abxR: None; putative secreted antimicrobial designed as CDS to be fused to a C-terminal linker and affinity tag or fluorescent marker; this GB part is expressed by Western blot but not functionally characterized in Dicty; template source: Dicty gDNA or gDNA library |
| pUPD2_DdExChr_GGAG_CGCT | Bacterial abxR: ampR; Dicty abxR: None; confers extrachromosomal maintenance in Dicty to any plasmid to which it is added; this GB part appears in vectors successfully transformed into Dicty but their extrachromosomal status has not been assessed; template source: pDM1210 |
| pUPD2_dendra2_AATG_AGTG | Bacterial abxR: ampR; Dicty abxR: None; N-terminal photoswitchable fluorescent marker to be fused with a linker to a CDS of interest; a representative of this GB part is validated (including the ability to photoswitch) in Dicty by microscopy; template source: pDdDendra2 |
| pUPD2_dendra2_AATG_GCTT | Bacterial abxR: ampR; Dicty abxR: None; photoswitchable fluorescent marker as a standalone ORF; this GB part is validated (including the ability to photoswitch) in Dicty by microscopy; template source: pDdDendra2 |
| pUPD2_dendra2_CTCG_GCTT | Bacterial abxR: ampR; Dicty abxR: None; C-terminal photoswitchable fluorescent marker to be fused with a linker to a CDS of interest; a representative of this GB part is validated (including the ability to photoswitch) in Dicty by microscopy; template source: pDdDendra2 |
| pUPD2_dscA_AATG_TCGG | Bacterial abxR: ampR; Dicty abxR: None; discoidin as a CDS to be fused to a C-terminal linker and affinity tag or fluorescent marker; this GB part is expressed in Dicty as determined by microscopy of a fluorescent protein-fusion; template source: Dicty gDNA or gDNA library |
| pUPD2_dscA_TAGC_TCGG | Bacterial abxR: ampR; Dicty abxR: None; discoidin as a CDS to be fused to N- and C-terminal linkers and affinity tags or fluorescent markers; a representative of this GB part is expressed in Dicty as determined by microscopy of a fluorescent protein-fusion; template source: Dicty gDNA or gDNA library |
| pUPD2_dscAp_GGAG_AATG | Bacterial abxR: ampR; Dicty abxR: None; overexpression promoter; this GB part is validated in Dicty by microscopy; template source: TOPO vector containing dscAp |
| pUPD2_ecmAp_GGAG_AATG | Bacterial abxR: ampR; Dicty abxR: None; prestalk-specific promoter; this GB part is not validated in Dicty; template source: Dicty gDNA or gDNA library |
| pUPD2_eYFP_AATG_AGTG | Bacterial abxR: ampR; Dicty abxR: None; N-terminal fluorescent marker to be fused with a linker to a CDS of interest; a representative of this GB part is validated in Dicty by microscopy; template source: pUPD2 eYFP (which was previously not Dicty codon-optimized) |
| pUPD2_eYFP_AATG_GCTT | Bacterial abxR: ampR; Dicty abxR: None; fluorescent marker as a standalone ORF; this GB part is validated in Dicty by microscopy; template source: pUPD2 eYFP (which was previously not Dicty codon-optimized) |
| pUPD2_eYFP_CTCG_GCTT | Bacterial abxR: ampR; Dicty abxR: None; C-terminal fluorescent marker to be fused with a linker to a CDS of interest; a representative of this GB part is validated in Dicty by microscopy; template source: pUPD2 eYFP (which was previously not Dicty codon-optimized) |
| pUPD2_FfLuc_AATG_GCTT | Bacterial abxR: ampR; Dicty abxR: None; firefly luciferase as a standalone ORF; this GB part is not validated in Dicty; template source: pUPD2 Firefly Luciferase (which was previously not Dicty codon-optimized) |
| pUPD2_flamindo2_AATG_GCTT | Bacterial abxR: ampR; Dicty abxR: None; single-channel mCitrine-fused cAMP sensor as a standalone ORF; this GB part is validated in Dicty by microscopy; template source: pcDNA3.1 Flamindo2 |
| pUPD2_Flexible_Linker_  AGTGGC_GGTAGC | Bacterial abxR: ampR; Dicty abxR: None; flexible linker designed to fuse an N-terminal tag or marker to a CDS; this GB part is validated in Dicty by its presence in successfully expressed and localized fusion proteins; template source: dsDNA oligo |
| pUPD2_Flexible_Linker_  TCGGGC_GGCTCG | Bacterial abxR: ampR; Dicty abxR: None; flexible linker designed to fuse an C-terminal tag or marker to a CDS; this GB part is validated in Dicty by its presence in successfully expressed and localized fusion proteins; template source: dsDNA oligo |
| pUPD2_GGGSx4_Linker_  AGTGGC_GGTAGC | Bacterial abxR: ampR; Dicty abxR: None; flexible linker designed to fuse an N-terminal tag or marker to a CDS; this GB part is validated in Dicty by its presence in successfully expressed and localized fusion proteins; template source: dsDNA oligo |
| pUPD2_GGGSx4_Linker_  TCGGGC_GGCTCG | Bacterial abxR: ampR; Dicty abxR: None; flexible linker designed to fuse an C-terminal tag or marker to a CDS; this GB part is validated in Dicty by its presence in successfully expressed and localized fusion proteins; template source: dsDNA oligo |
| pUPD2_gghA_AATG_TCGG | Bacterial abxR: ampR; Dicty abxR: None; putative secreted antimicrobial designed as a CDS to be fused to a C-terminal linker and affinity tag or fluorescent marker; this GB part is not validated in Dicty; template source: Dicty gDNA or gDNA library |
| pUPD2_GST_AATG_AGTG | Bacterial abxR: ampR; Dicty abxR: None; N-terminal affinity tag to be fused with a linker to a CDS of interest; contains thrombin cleavage site; this GB part is validated in Dicty by Western blot; template source: pDM193 |
| pUPD2_GST_CTCG_GCTT | Bacterial abxR: ampR; Dicty abxR: None; C-terminal affinity tag to be fused with a linker to a CDS of interest; contains thrombin cleavage site; this GB part is validated in Dicty by Western blot; template source: pDM193 |
| pUPD2_gtaC_AATG_TCGG | Bacterial abxR: ampR; Dicty abxR: None; transcription factor designed as CDS to be fused to a C-terminal linker and affinity tag or fluorescent marker; this GB part is validated in Dicty by microscopy; template source: Dicty gDNA or gDNA library |
| pUPD2_H2Bv3_TAGC_TCGG | Bacterial abxR: ampR; Dicty abxR: None; nuclear marker as a CDS to be fused to N- and C-terminal linkers and affinity tags or fluorescent markers; this GB part is validated in Dicty by microscopy; template source: pDM304 mCherry-H2B |
| pUPD2_HA_AATG_AGTG | Bacterial abxR: ampR; Dicty abxR: None; N-terminal affinity tag to be fused with a linker to a CDS of interest; this GB part is validated in Dicty by Western blot; template source: dsDNA oligo |
| pUPD2_HA_CTCG_GCTT | Bacterial abxR: ampR; Dicty abxR: None; C-terminal affinity tag to be fused with a linker to a CDS of interest; a representative of this GB part is validated in Dicty by Western blot; template source: dsDNA oligo |
| pUPD2_hcpB_TAGC_GCTT | Bacterial abxR: ampR; Dicty abxR: None; sub-nuclear marker as a CDS to be fused to an N-terminal linker and affinity tag or fluorescent marker; this GB part is not validated in Dicty; template source: Dicty gDNA or gDNA library |
| pUPD2_hygR_AATG_GCTT | Bacterial abxR: ampR; Dicty abxR: None; hygR as a standalone ORF; this GB part is validated in Dicty by successful transformation and drug selection; pDM358 |
| pUPD2_hypaCas9_AATG_TCGG | Bacterial abxR: ampR; Dicty abxR: None; high-fidelity variant of SpCas9 as a CDS to be fused to a C-terminal linker and affinity tag or fluorescent marker; this GB part is not validated in Dicty; template sources: dsDNA oligo, pJSC173 |
| pUPD2_iliE-1p_GGAG_AATG | Bacterial abxR: ampR; Dicty abxR: None; iliE-1 promoter; this GB part is not validated in Dicty; template source: Dicty gDNA or gDNA library |
| pUPD2_loxP-coaAp_  GGAG_AATG | Bacterial abxR: ampR; Dicty abxR: None; overexpression promoter flanked 5' by one loxP site; the coaA promoter is validated in Dicty by successful transformation and microscopy, but excision of a Flox'd element by Cre recombinase has not been assessed; template source: Dicty gDNA or gDNA library |
| pUPD2_mCerulean_AATG_AGTG | Bacterial abxR: ampR; Dicty abxR: None; N-terminal fluorescent marker to be fused with a linker to a CDS of interest; this GB part is validated in Dicty by microscopy; template source: pDXA mCerulean |
| pUPD2_mCerulean_AATG_GCTT | Bacterial abxR: ampR; Dicty abxR: None; fluorescent marker as a standalone ORF; this GB part is validated in Dicty by microscopy; template source: pDXA mCerulean |
| pUPD2_mCerulean_CTCG_GCTT | Bacterial abxR: ampR; Dicty abxR: None; C-terminal fluorescent marker to be fused with a linker to a CDS of interest; this GB part is validated in Dicty by microscopy; template source: pDXA mCerulean |
| pUPD2_mCherry_AATG_AGTG | Bacterial abxR: ampR; Dicty abxR: None; N-terminal fluorescent marker to be fused with a linker to a CDS of interest; a representative of this GB part is validated in Dicty by microscopy; template source: pDM1210 |
| pUPD2_mCherry_AATG_GCTT | Bacterial abxR: ampR; Dicty abxR: None; fluorescent marker as a standalone ORF; this GB part is validated in Dicty by microscopy; template source: pDM1210 |
| pUPD2_mCherry_CTCG_GCTT | Bacterial abxR: ampR; Dicty abxR: None; C-terminal fluorescent marker to be fused with a linker to a CDS of interest; a representative of this GB part is validated in Dicty by microscopy; template source: pDM1210 |
| pUPD2_mhcAt_GCTT_CAGT | Bacterial abxR: ampR; Dicty abxR: None; terminator designed to flank an additional 3' part such as a barcode; this GB part is validated in Dicty by successful transformation; template source: Dicty gDNA or gDNA library |
| pUPD2_mhcAt_GCTT_CGCT | Bacterial abxR: ampR; Dicty abxR: None; terminator; this GB part is validated in Dicty; template source: Dicty gDNA or gDNA library |
| pUPD2_mhcAt_loxP_  GCTT_CGCT | Bacterial abxR: ampR; Dicty abxR: None; terminator flanked 3' by one loxP site; the mhcA terminator is validated in Dicty by successful transformation and microscopy, but excision of a Flox'd element by Cre recombinase has not been assessed; template source: Dicty gDNA or gDNA library |
| pUPD2_mIFP_AATG_AGTG | Bacterial abxR: ampR; Dicty abxR: None; N-terminal fluorescent marker to be fused with a linker to a CDS of interest; mIFP requires brief exposure to a biliverdin cofactor at 50 µM to fluoresce; a representative of this GB part is validated in Dicty by microscopy; template source: pUPD2 mIFP (which was previously not Dicty codon-optimized) |
| pUPD2_mIFP_AATG_GCTT | Bacterial abxR: ampR; Dicty abxR: None; fluorescent marker as a standalone ORF; mIFP requires brief exposure to a biliverdin cofactor at 50 µM to fluoresce; this GB part is validated in Dicty by microscopy; template source: pUPD2 mIFP (which was previously not Dicty codon-optimized) |
| pUPD2_mIFP_CTCG_GCTT | Bacterial abxR: ampR; Dicty abxR: None; C-terminal fluorescent marker to be fused with a linker to a CDS of interest; mIFP requires brief exposure to a biliverdin cofactor at 50 µM to fluoresce; a representative of this GB part is validated in Dicty by microscopy; template source: pUPD2 mIFP (which was previously not Dicty codon-optimized) |
| pUPD2_mNeonGreen_AATG_  AGTG | Bacterial abxR: ampR; Dicty abxR: None; N-terminal fluorescent marker to be fused with a linker to a CDS of interest; a representative of this GB part is validated in Dicty by microscopy; template source: pUPD2 mNeonGreen (which was previously not Dicty codon-optimized) |
| pUPD2_mNeonGreen_AATG_  GCTT | Bacterial abxR: ampR; Dicty abxR: None; fluorescent marker as a standalone ORF; this GB part is validated in Dicty by microscopy; template source: pUPD2 mNeonGreen (which was previously not Dicty codon-optimized) |
| pUPD2_mNeonGreen_AATG_  GGTAGC | Bacterial abxR: ampR; Dicty abxR: None; N-terminal fluorescent marker to be fused without a linker to a CDS of interest; a representative of this GB part is validated in Dicty by microscopy; template source: pUPD2 mNeonGreen (which was previously not Dicty codon-optimized) |
| pUPD2_mNeonGreen_CTCG_  GCTT | Bacterial abxR: ampR; Dicty abxR: None; C-terminal fluorescent marker to be fused with a linker to a CDS of interest; a representative of this GB part is validated in Dicty by microscopy; template source: pUPD2 mNeonGreen (which was previously not Dicty codon-optimized) |
| pUPD2_myc_CTCG_GCTT | Bacterial abxR: ampR; Dicty abxR: None; C-terminal affinity tag to be fused with a linker to a CDS of interest; this GB part is not validated in Dicty; template source: dsDNA oligo |
| pUPD2_neoR_AATG_GCTT | Bacterial abxR: ampR; Dicty abxR: None; neoR as a standalone ORF; this GB part is validated in Dicty by successful transformation and drug selection; template source: pDXA sfGFP |
| pUPD2_PHdagA_AATG_TCGG | Bacterial abxR: ampR; Dicty abxR: None; pleckstrin-homology domain of dagA; leading edge marker; this GB part is validated in Dicty by microscopy; template source: pDM358 PH-GFP |
| pUPD2_pinkflamindo_AATG_  GCTT | Bacterial abxR: ampR; Dicty abxR: None; single-channel mApple-fused cAMP sensor as a standalone ORF; this GB part is validated in Dicty by microscopy; template source: pcDNA3.1 Pink Flamindo |
| pUPD2_ralGDS_TAGC_GCTT | Bacterial abxR: ampR; Dicty abxR: None; sensor that binds to GTP-bound (activated) RapA designed as a CDS to be fused to an N-terminal linker and affinity or fluorescent marker; this GB part is not validated in Dicty; template source: pDM115 |
| pUPD2_renilla_AATG_GCTT | Bacterial abxR: ampR; Dicty abxR: None; renilla luciferase as a standalone ORF; this GB part is validated in Dicty by luciferase reporter assay; template source: pUPD2 renilla (which was previously not Dicty codon-optimized) |
| pUPD2_sfGFP_AATG_AGTG | Bacterial abxR: ampR; Dicty abxR: None; N-terminal fluorescent marker to be fused with a linker to a CDS of interest; this GB part is validated in Dicty by microscopy; template source: pDXA sfGFP |
| pUPD2_sfGFP_AATG_GCTT | Bacterial abxR: ampR; Dicty abxR: None; fluorescent marker as a standalone ORF; this GB part is validated in Dicty by microscopy; template source: pDXA sfGFP |
| pUPD2_sfGFP_CTCG_GCTT | Bacterial abxR: ampR; Dicty abxR: None; C-terminal fluorescent marker to be fused with a linker to a CDS of interest; this GB part is validated in Dicty by microscopy; template source: pDXA sfGFP |
| pUPD2_SpCas9_SV40NLS_  eGFP_AATG_GCTT | Bacterial abxR: ampR; Dicty abxR: None; SpCas9 with a nuclear localization signal and fused to eGFP; this GB part is validated in Dicty by successful generation of short indels when pDGB_A2[CRISPR1] with a specific sgRNA is transiently transformed; template source: pTM1285 |
| pUPD2_SV40NLS_AATG_AGTG | Bacterial abxR: ampR; Dicty abxR: None; N-terminal nuclear localization signal to be fused with a linker to a CDS of interest; a representative of this GB part is validated in Dicty by its presence in pDGB_A2[CRISPR1], from which SpCas9 must be expressed and localized to the nucleus to successfully edit; template source: dsDNA oligo |
| pUPD2_SV40NLS_AGTGGC_  GGTAGC | Bacterial abxR: ampR; Dicty abxR: None; Nuclear localization signal designed as an N-terminal linker; a representative of this GB part is validated in Dicty by its presence in pDGB_A2[CRISPR1], from which SpCas9 must be expressed and localized to the nucleus to successfully edit; template source: dsDNA oligo |
| pUPD2_SV40NLS_CTCG_GCTT | Bacterial abxR: ampR; Dicty abxR: None; C-terminal nuclear localization signal to be fused with a linker to a CDS of interest; a representative of this GB part is validated in Dicty by its presence in pDGB_A2[CRISPR1], from which SpCas9 must be expressed and localized to the nucleus to successfully edit; template source: dsDNA oligo |
| pUPD2_SV40NLS_TCGGGC_  GGCTCG | Bacterial abxR: ampR; Dicty abxR: None; Nuclear localization signal designed as a C-terminal linker; a representative of this GB part is validated in Dicty by its presence in pDGB_A2[CRISPR1], from which SpCas9 must be expressed and localized to the nucleus to successfully edit; template source: dsDNA oligo |
| pUPD2_tagBFP_AATG_AGTG | Bacterial abxR: ampR; Dicty abxR: None; N-terminal fluorescent marker to be fused with a linker to a CDS of interest; a representative of this GB part is validated in Dicty by microscopy; template source: pUPD2 tagBFP (which was not previously Dicty codon-optimized) |
| pUPD2_tagBFP_AATG_GCTT | Bacterial abxR: ampR; Dicty abxR: None; fluorescent marker as a standalone ORF; this GB part is validated in Dicty by microscopy; template source: pUPD2 tagBFP (which was not previously Dicty codon-optimized) |
| pUPD2_tagBFP_AATG_GGTAGC | Bacterial abxR: ampR; Dicty abxR: None; N-terminal fluorescent marker to be fused without a linker to a CDS of interest; a representative of this GB part is validated in Dicty by microscopy; template source: pUPD2 tagBFP (which was not previously Dicty codon-optimized) |
| pUPD2_tagBFP_CTCG_GCTT | Bacterial abxR: ampR; Dicty abxR: None; C-terminal fluorescent marker to be fused with a linker to a CDS of interest; a representative of this GB part is validated in Dicty by microscopy; template source: pUPD2 tagBFP (which was not previously Dicty codon-optimized) |
| pUPD2_tgrBCp_GGAG_AATG | Bacterial abxR: ampR; Dicty abxR: None; bidirectional tgrB1 and tgrC1 promoter; this GB part is not validated in Dicty; template source: Dicty gDNA or gDNA library |
| pUPD2_thyAp_GGAG_AATG | Bacterial abxR: ampR; Dicty abxR: None; putative light-inducible promoter; this GB part is validated in Dicty by microscopy; template source: Dicty gDNA or gDNA library |
| pUPD2_tRNA_sgRNAtemplate_  GGAG_GCTT | Bacterial abxR: ampR; Dicty abxR: None; Isoleucine tRNA fused to an sgRNA template into which specific guide oligos can be cloned using *Bbs*I or *Bpi*I restriction enzyme; this GB part is validated in Dicty by its presence in pDGB_A2[CRISPR1], which must transcribe a tRNα-sgRNA fusion to successfully edit; template source: pTM1285 |
| pDGB_α1[*act15/ctsZ-GST*] | Bacterial abxR: kanR; Dicty abxR: None; ctsZ-GST overexpression intermediate vector; this assembly is not validated in Dicty |
| pDGB_α1[*act15/ctsZ-mNeonGreen*] | Bacterial abxR: kanR; Dicty abxR: None; ctsZ-mNeonGreen overexpression intermediate vector; this assembly is not validated in Dicty |
| pDGB_α1[*act15/DDB_G0278295-GST*] | Bacterial abxR: kanR; Dicty abxR: None; DDB_G0278295-GST overexpression intermediate vector; an Ω-level assembly with this unit is expressed in Dicty by Western blot, but is not functionally characterized |
| pDGB_α1[*act15/DDB_G0278295-mNeonGreen*] | Bacterial abxR: kanR; Dicty abxR: None; DDB_G0278295-mNeonGreen overexpression intermediate vector; this assembly is not validated in Dicty |
| pDGB_α1[*act15/DDB_G0289731-GST*] | Bacterial abxR: kanR; Dicty abxR: None; DDB_G0289731-GST overexpression intermediate vector; an Ω-level assembly with this unit is expressed in Dicty by Western blot, but is not functionally characterized |
| pDGB_α1[*act15/FfLuc/act8*] | Bacterial abxR: kanR; Dicty abxR: None; firefly luciferase overexpression intermediate vector; this assembly is not validated in Dicty |
| pDGB_α1[*act15/FfLuc/mhcA*] | Bacterial abxR: kanR; Dicty abxR: None; firefly luciferase overexpression intermediate vector; this assembly is not validated in Dicty |
| pDGB_α1[*act15/gghA-GST*] | Bacterial abxR: kanR; Dicty abxR: None; gghα-GST overexpression intermediate vector; this assembly is not validated in Dicty |
| pDGB_α1[*act15/gghA-mNeonGreen*] | Bacterial abxR: kanR; Dicty abxR: None; gghα-mNeonGreen overexpression intermediate vector; this assembly is not validated in Dicty |
| pDGB_α1[*act15/GST-H2Bv3-mNeonGreen*] | Bacterial abxR: kanR; Dicty abxR: None; GST-H2Bv3-mNeonGreen overexpression intermediate vector; this assembly is not validated in Dicty |
| pDGB_α1[*act15/GST-iliE-1*] | Bacterial abxR: kanR; Dicty abxR: None; GST-iliE-1 overexpression intermediate vector; an Ω-level assembly with this assembly is successfully transformed into Dicty |
| pDGB_α1[*act15/HA-H2Bv3-mIFP*] | Bacterial abxR: kanR; Dicty abxR: None; Hα-H2Bv3-mIFP overexpression intermediate vector; mIFP requires brief exposure to a biliverdin cofactor at 50 µM to fluoresce;this assembly is not validated in Dicty |
| pDGB_α1[*act15/mIFP*] | Bacterial abxR: kanR; Dicty abxR: None; mIFP overexpression intermediate vector; mIFP requires brief exposure to a biliverdin cofactor at 50 µM to fluoresce;an Ω-level assembly with this unit is validated in Dicty by microscopy |
| pDGB_α1[*act15/mIFP-H2Bv3-HA*] | Bacterial abxR: kanR; Dicty abxR: None; mIFP-H2Bv3-HA overexpression intermediate vector; mIFP requires brief exposure to a biliverdin cofactor at 50 µM to fluoresce;this assembly is not validated in Dicty |
| pDGB_α1[*act15/mNeonGreen/*  *act8*] | Bacterial abxR: kanR; Dicty abxR: None; mNeonGreen overexpression intermediate vector; an Ω-level assembly with this unit is validated in Dicty by microscopy |
| pDGB_α1[*act15/mNeonGreen-H2Bv3-GST*] | Bacterial abxR: kanR; Dicty abxR: None; mNeonGreen-H2Bv3-GST overexpression intermediate vector; this assembly is not validated in Dicty |
| pDGB_α1[*act15/mNeonGreen-ralGDS*] | Bacterial abxR: kanR; Dicty abxR: None; mNeonGreen-ralGDS overexpression intermediate vector; an Ω-level assembly with this unit is successfully transformed into Dicty |
| pDGB_α1[*act15/mNeonGreen/*  *mhcA*] | Bacterial abxR: kanR; Dicty abxR: None; mNeonGreen overexpression intermediate vector; although all parts present in this assembly are validated in Dicty in other assemblies, this assembly itself is not validated in Dicty |
| pDGB_α1[*act15/myc-H2Bv3-tagBFP*] | Bacterial abxR: kanR; Dicty abxR: None; myc-H2Bv3-tagBFP overexpression intermediate vector; this assembly is not validated in Dicty |
| pDGB_α1[*act15/myc-ralGDS*] | Bacterial abxR: kanR; Dicty abxR: None; myc-ralGDS overexpression intermediate vector; an Ω-level assembly with this unit is transformed into Dicty |
| pDGB_α1[*act15/renilla/act8*] | Bacterial abxR: kanR; Dicty abxR: None; renilla luciferase overexpression intermediate vector; although all parts present in this assembly are validated in Dicty in other assemblies, this assembly itself is not validated in Dicty |
| pDGB_α1[*act15/renilla/mhcA*] | Bacterial abxR: kanR; Dicty abxR: None; renilla luciferase overexpression intermediate vector; an assembly with this unit is validated in Dicty by luciferase reporter assay |
| pDGB_α1[*act15/SpCas9-SV40NLS-eGFP*] | Bacterial abxR: kanR; Dicty abxR: None; SpCas9-NLS-eGFP overexpression intermediate vector; this unit is validated in pDGB_A2[CRISPR1] |
| pDGB_α1[*act15/tagBFP*] | Bacterial abxR: kanR; Dicty abxR: None; tagBFP overexpression intermediate vector; an Ω-level assembly with this unit is validated in Dicty by microscopy |
| pDGB_α1[*act15/tagBFP-H2Bv3-myc*] | Bacterial abxR: kanR; Dicty abxR: None; tagBFP-H2Bv3-myc overexpression intermediate vector; this unit is not validated in Dicty |
| pDGB_α1[*act15/tagBFP-ralGDS*] | Bacterial abxR: kanR; Dicty abxR: None; tagBFP-ralGDS overexpression intermediate vector; this unit is not validated in Dicty |
| pDGB_α1[*aurK/sfGFP*] | Bacterial abxR: kanR; Dicty abxR: None; sfGFP driven by the putatively light-sensitive aurK promoter; intermediate vector; an Ω-level assembly with this unit is validated in Dicty by microscopy |
| pDGB_α1[*coaA/bsR*] | Bacterial abxR: kanR; Dicty abxR: bsR; bsR selectable cassette; multiple assemblies that contain this unit are validated in Dicty by successful transformation and drug selection |
| pDGB_α1[*coaA/dendra2*] | Bacterial abxR: kanR; Dicty abxR: None; dendra2 overexpression intermediate vector; an Ω-level assembly with this unit is validated in Dicty by microscopy (including the ability to photoswitch) |
| pDGB_α1[*coaA/eYFP*] | Bacterial abxR: kanR; Dicty abxR: None; eYFP overexpression intermediate vector; an Ω-level assembly with this unit is validated in Dicty by microscopy |
| pDGB_α1[*coaA/FfLuc/act8*] | Bacterial abxR: kanR; Dicty abxR: None; firefly luciferase overexpression intermediate vector; this assembly is not validated in Dicty |
| pDGB_α1[*coaA/FfLuc/mhcA*] | Bacterial abxR: kanR; Dicty abxR: None; firefly luciferase overexpression intermediate vector; this assembly is not validated in Dicty |
| pDGB_α1[*coaA/hygR*] | Bacterial abxR: kanR; Dicty abxR: hygR; hygR selectable cassette; an Ω-level assembly with this unit is validated in Dicty by successful transformation and drug selection |
| pDGB_α1[*coaA/hypaCas9-SV40NLS-sfGFP*] | Bacterial abxR: kanR; Dicty abxR: None; high-fidelity SpCas9 overexpression intermediate vector; this unit appears in pDGB_A1_hypaCRISPR1, which is not validated in Dicty |
| pDGB_α1[*coaA/mCerulean*] | Bacterial abxR: kanR; Dicty abxR: None; mCerulean overexpression intermediate vector; an Ω-level assembly with this unit is validated in Dicty by microscopy |
| pDGB_α1[*coaA/mNeonGreen/*  *act8*] | Bacterial abxR: kanR; Dicty abxR: None; mNeonGreen overexpression intermediate vector; although all parts present in this assembly are validated in Dicty in other assemblies, this assembly itself is not validated in Dicty |
| pDGB_α1[*coaA/mNeonGreen/*  *mhcA*] | Bacterial abxR: kanR; Dicty abxR: None; mNeonGreen overexpression intermediate vector; although all parts present in this assembly are validated in Dicty in other assemblies, this assembly itself is not validated in Dicty |
| pDGB_α1[*coaA/neoR*] | Bacterial abxR: kanR; Dicty abxR: neoR; neoR selectable cassette; multiple assemblies that contain this unit are validated in Dicty by successful transformation and drug selection |
| pDGB_α1[*coaA/PH-tagBFP*] | Bacterial abxR: kanR; Dicty abxR: None; PH-tagBFP overexpression intermediate vector; this assembly is not validated in Dicty |
| pDGB_α1[*coaA/renilla/act8*] | Bacterial abxR: kanR; Dicty abxR: None; renilla luciferase overexpression intermediate vector; although all parts present in this assembly are validated in Dicty in other assemblies, this assembly itself is not validated in Dicty |
| pDGB_α1[*coaA/renilla/mhcA*] | Bacterial abxR: kanR; Dicty abxR: None; renilla luciferase overexpression intermediate vector; although all parts present in this assembly are validated in Dicty in other assemblies, this assembly itself is not validated in Dicty |
| pDGB_α1[*coaA/SpCas9-SV40NLS-eGFP*] | Bacterial abxR: kanR; Dicty abxR: None; SpCas9-NLS-eGFP overexpression intermediate vector; this assembly is not validated in Dicty |
| pDGB_α1[*coaA/tagBFP-H2Bv3-7xHis*] | Bacterial abxR: kanR; Dicty abxR: None; tagBFP-H2Bv3-7xHis overexpression intermediate vector; this assembly is not validated in Dicty |
| pDGB_α1[*DDB_G0288429/sfGFP*] | Bacterial abxR: kanR; Dicty abxR: None; sfGFP driven by the putatively light-sensitive DDB_G0288429 promoter; intermediate vector; an Ω-level assembly with this unit is validated in Dicty by microscopy |
| pDGB_α1[DdExChr] | Bacterial abxR: kanR; Dicty abxR: None; confers extrachromosomal maintenance in Dicty to any plasmid to which it is added; DdExChr appears in vectors successfully transformed into Dicty but their extrachromosomal status has not been assessed |
| pDGB_α1[*dscA/dendra2*] | Bacterial abxR: kanR; Dicty abxR: None; photoswitchable-FP dendra2 driven by dscA promoter; intermediate vector; this unit is not validated in Dicty |
| pDGB_α1[*dscA/eYFP*] | Bacterial abxR: kanR; Dicty abxR: None; eYFP driven by dscA promoter; intermediate vector; an Ω-level assembly with this unit is validated in Dicty by microscopy |
| pDGB_α1[*dscA/mCerulean*] | Bacterial abxR: kanR; Dicty abxR: None; mCerulean driven by dscA promoter; intermediate vector; this assembly is not validated in Dicty |
| pDGB_α1[*dscA/mCherry*] | Bacterial abxR: kanR; Dicty abxR: None; mCherry driven by dscA promoter; intermediate vector; this assembly is not validated in Dicty |
| pDGB_α1[hypaCRISPR1] | Bacterial abxR: kanR; Dicty abxR: neoR; high-fidelity SpCas9 + tRNA/sgRNAtemplate expression vector; specific guide oligos can be cloned using *Bbs*I or *Bpi*I restriction enzyme; visual screening for successful ligation of specific guide oligos by lacZ expression in bacteria; this assembly is not validated in Dicty |
| pDGB_α1[stuffer] | Bacterial abxR: kanR; Dicty abxR: None; MCS used to occupy an assembly position while shuttling other assemblies between GB backbones; the stuffer has been successfully transformed into Dicty |
| pDGB_α1[*thyA/sfGFP*] | Bacterial abxR: kanR; Dicty abxR: None; sfGFP driven by the putatively light-sensitive thyA promoter; intermediate vector; an Ω-level assembly with this unit is validated in Dicty by microscopy |
| pDGB_α1B[*coaA/dscA-HA*] | Bacterial abxR: kanR; Dicty abxR: bsR; dscα-HA overexpression vector; this assembly is not validated in Dicty |
| pDGB_α1B[*coaA/dscA-sfGFP*] | Bacterial abxR: kanR; Dicty abxR: bsR; dscα-sfGFP overexpression vector; this assembly is not validated in Dicty |
| pDGB_α1B[*dscA/dscA-HA*] | Bacterial abxR: kanR; Dicty abxR: bsR; dscα-HA native promoter expression vector; this assembly is not validated in Dicty |
| pDGB_α1H[*coaA/dscA-HA*] | Bacterial abxR: kanR; Dicty abxR: hygR; dscα-HA overexpression vector; this assembly is not validated in Dicty |
| pDGB_α1H[*dscA/dscA-HA*] | Bacterial abxR: kanR; Dicty abxR: hygR; dscα-HA native promoter expression vector; this assembly is not validated in Dicty |
| pDGB_α1H[*dscA/dscA-sfGFP*] | Bacterial abxR: kanR; Dicty abxR: hygR; dscα-sfGFP native promoter expression vector; this assembly is not validated in Dicty |
| pDGB_α1N[*coaA/dscA-7xHis*] | Bacterial abxR: kanR; Dicty abxR: neoR; dscα-7xHis overexpression vector; this assembly is not validated in Dicty |
| pDGB_α1N[*coaA/dscA-GST*] | Bacterial abxR: kanR; Dicty abxR: neoR; dscα-GST overexpression vector; this assembly is not validated in Dicty |
| pDGB_α1N[*coaA/dscA-HA*] | Bacterial abxR: kanR; Dicty abxR: neoR; dscα-HA overexpression vector; this assembly is not validated in Dicty |
| pDGB_α1N[*coaA/dscA-myc*] | Bacterial abxR: kanR; Dicty abxR: neoR; dscα-myc overexpression vector; this assembly is not validated in Dicty |
| pDGB_α1N[*coaA/flamindo2*] | Bacterial abxR: kanR; Dicty abxR: neoR; mCitrine-fused single-channel cAMP sensor overexpression vector; this assembly is validated in Dicty by microscopy |
| pDGB_α1N[*coaA/pinkflamindo*] | Bacterial abxR: kanR; Dicty abxR: neoR; mApple-fused single-channel cAMP sensor overexpression vector; this assembly is validated in Dicty by microscopy |
| pDGB_α1N[*dscA/dscA-HA*] | Bacterial abxR: kanR; Dicty abxR: neoR; dscα-HA native promoter expression vector; this assembly is not validated in Dicty |
| pDGB_α1N[*dscA/dscA-sfGFP*] | Bacterial abxR: kanR; Dicty abxR: neoR; dscα-sfGFP native promoter expression vector; this assembly is expressed in Dicty by microscopy |
| pDGB_α1N[*iliE-1/sfGFP*] | Bacterial abxR: kanR; Dicty abxR: neoR; sfGFP driven by iliE-1 promoter; this assembly is not validated in Dicty |
| pDGB_α2[*act15/mCherry*] | Bacterial abxR: kanR; Dicty abxR: None; mCherry overexpression intermediate vector; this assembly is not validated in Dicty |
| pDGB_α2[*act15/mNeonGreen*] | Bacterial abxR: kanR; Dicty abxR: None; mNeonGreen overexpression intermediate vector; an Ω-level assembly with this unit is validated in Dicty |
| pDGB_α2[*act15/mNeonGreen*; *coaA/neoR*; DdExChr; stuffer] | Bacterial abxR: kanR; Dicty abxR: neoR; mNeonGreen overexpression vector; this assembly is not validated in Dicty |
| pDGB_α2[Flox'd *coaA/bsR*; *act15/renilla/act8*; DdExChr; stuffer] | Bacterial abxR: kanR; Dicty abxR: bsR; renilla luciferase overexpression vector; this assembly is not validated in Dicty |
| pDGB_α2[Flox'd *coaA/bsR*; *act15/renilla/mhcA*; DdExChr; stuffer] | Bacterial abxR: kanR; Dicty abxR: bsR; renilla luciferase overexpression vector; this assembly is not validated in Dicty |
| pDGB_α2[*act15/sfGFP-H2B-HA*] | Bacterial abxR: kanR; Dicty abxR: None; sfGFP-H2B-HA overepxression intermediate vector; an Ω-level assembly with a similar unit is validated in Dicty by microscopy |
| pDGB_α2[*act15/sfGFP*] | Bacterial abxR: kanR; Dicty abxR: None; sfGFP overexpression vector; an Ω-level assembly with this unit is validated in Dicty |
| pDGB_α2[*act15/Cas9-NLS-eGFP;* tRNA/sgRNAtemplate; *coaA/neoR*] | Bacterial abxR: kanR; Dicty abxR: neoR; this is the pDGB_A2[CRISPR1] vector, but it lacks visual screening for ligation of specific sgRNAs by lacZ; pDGB_A2[CRISPR1] is validated in Dicty |
| pDGB_α2[*coaA/bsR*] | Bacterial abxR: kanR; Dicty abxR: bsR; bsR selectable cassette; multiple assemblies that contain this unit are validated in Dicty |
| pDGB_α2[*coaA/hygR*] | Bacterial abxR: kanR; Dicty abxR: hygR; hygR selectable cassette; an Ω-level assembly with this unit is validated in Dicty |
| pDGB_α2[*coaA/mCherry*] | Bacterial abxR: kanR; Dicty abxR: None; mCherry overexpression intermediate vector; this assembly is not validated in Dicty |
| pDGB_α2[*coaA/mNeonGreen-ralGDS*] | Bacterial abxR: kanR; Dicty abxR: None; mNeonGreen-ralGDS overexpression intermediate vector; an Ω-level assembly with this unit is transformed into Dicty |
| pDGB_α2[*coaA/neoR*] | Bacterial abxR: kanR; Dicty abxR: neoR; neoR selectable cassette; multiple assemblies that contain this unit are validated in Dicty |
| pDGB_α2[Flox'd coaA/bsR; coaA/renilla/act8; DdExChr; stuffer] | Bacterial abxR: kanR; Dicty abxR: bsR; renilla luciferase overexpression vector; this assembly is not validated in Dicty |
| pDGB_α2[Flox'd coaA/bsR; coaA/renilla/mhcA; DdExChr; stuffer] | Bacterial abxR: kanR; Dicty abxR: bsR; renilla luciferase overexpression vector; this assembly is not validated in Dicty |
| pDGB_α2[*coaA/sfGFP-H2B-HA*] | Bacterial abxR: kanR; Dicty abxR: None; sfGFP-H2B-HA overepxression intermediate vector; an Ω-level assembly with this unit is validated in Dicty |
| pDGB_α2[*coaA/sfGFP-ralGDS*] | Bacterial abxR: kanR; Dicty abxR: None; sfGFP-ralGDS overexpression intermediate vector; this assembly is not validated in Dicty by microscopy |
| pDGB_α2[*coaA/sfGFP*] | Bacterial abxR: kanR; Dicty abxR: None; sfGFP overexpression vector; an Ω-level assembly with this unit is validated in Dicty by microscopy |
| pDGB_α2[CRISPR1] | Bacterial abxR: kanR; Dicty abxR: neoR; SpCas9 + tRNA/sgRNAtemplate expression vector; specific guide oligos can be cloned using *Bbs*I or *Bpi*I restriction enzyme; visual screening for successful ligation of specific guide oligos by lacZ expression in bacteria; this assembly is validated in Dicty by successful generation of short indels when this plasmid with a specific sgRNA is transiently transformed |
| pDGB_α2[CRISPR1_acaAsg1] | Bacterial abxR: kanR; Dicty abxR: neoR; SpCas9 + acaA sgRNA expression vector; this assembly is validated in Dicty by the presence of aggregation- clones grown on KP SM plates |
| pDGB_α2[CRISPR1_rapgapBsg2] | Bacterial abxR: kanR; Dicty abxR: neoR; SpCas9 + rapgapB sgRNA expression vector; this assembly is validated in Dicty by successful generation of short indels in the rapgapB CDS and corresponding aberrant developmental morphology |
| pDGB_α2[DdExChr] | Bacterial abxR: kanR; Dicty abxR: None; confers extrachromosomal maintenance in Dicty to any plasmid to which it is added; DdExChr appears in vectors successfully transformed into Dicty but their extrachromosomal status has not been assessed |
| pDGB_α2[DdExChr; *coaA/neoR; coaA/sfGFP*] | Bacterial abxR: kanR; Dicty abxR: neoR; sfGFP overexpression vector; this assembly is validated in Dicty by microscopy |
| pDGB_α2[Flox'd *coaA/bsR*] | Bacterial abxR: kanR; Dicty abxR: bsR; bsR selectable cassette; this assembly is successfully selected for in Dicty but excision by Cre recombinase has not been assessed |
| pDGB_α2[Flox'd *coaA/hygR*] | Bacterial abxR: kanR; Dicty abxR: hygR; hygR selectable cassette; this assembly is successfully selected for in Dicty but excision by Cre recombinase has not been assessed |
| pDGB_α2[stuffer] | Bacterial abxR: kanR; Dicty abxR: None; MCS used to occupy an assembly position while shuttling other assemblies between GB backbones; the stuffer has been successfully transformed into Dicty |
| pDGB_α2[tRNA/sgRNAtemplate/  act8] | Bacterial abxR: kanR; Dicty abxR: None; Isoleucine tRNA fused to an sgRNA template into which specific guide oligos can be cloned using *Bbs*I or *Bpi*I restriction enzyme; this unit is validated in pDGB_A2[CRISPR1] by confirmation of gene editing |
| pDGB_α2[tRNA/sgRNAtemplate/mhcA] | Bacterial abxR: kanR; Dicty abxR: None; Isoleucine tRNA fused to an sgRNA template into which specific guide oligos can be cloned using *Bbs*I or *Bpi*I restriction enzyme; a similar assembly assembly [tRNA/sgRNAtemplate/act8] is validated in Dicty by confirmation gene editing |
| pDGB_α2B[*act15/FfLuc*] | Bacterial abxR: kanR; Dicty abxR: bsR; firefly luciferase overexpression vector; this assembly is not validated in Dicty |
| pDGB_αBN[*coaA/gtaC-mCherry*] | Bacterial abxR: kanR; Dicty abxR: neoR; gtaC-mCherry overexpression vector; an Ω-level assembly with this unit is validated in Dicty by microscopy |
| pDGB_αC[DdExChr] | Bacterial abxR: kanR; Dicty abxR: None; confers extrachromosomal maintenance in Dicty to any plasmid to which it is added; DdExChr appears in vectors successfully transformed into Dicty but their extrachromosomal status has not been assessed |
| pDGB_αC[Flox'd *coaA/bsR*] | Bacterial abxR: kanR; Dicty abxR: bsR; bsR selectable cassette; a similar assembly is successfully selected for in Dicty but excision by Cre recombinase has not been assessed |
| pDGB_αC[Flox'd *coaA/hygR*] | Bacterial abxR: kanR; Dicty abxR: hygR; hygR selectable cassette; a similar assembly is successfully selected for in Dicty but excision by Cre recombinase has not been assessed |
| pDGB_αC[stuffer] | Bacterial abxR: kanR; Dicty abxR: None; MCS used to occupy an assembly position while shuttling other assemblies between GB backbones; the stuffer has been successfully transformed into Dicty |
| pDGB_αCN[*coaA/PH-sfGFP*] | Bacterial abxR: kanR; Dicty abxR: neoR; PH-sfGFP overexpression vector; an Ω-level assembly with this unit is validated in Dicty by microscopy |
| pDGB_αD[DdExChr] | Bacterial abxR: kanR; Dicty abxR: None; confers extrachromosomal maintenance in Dicty to any plasmid to which it is added; DdExChr appears in vectors successfully transformed into Dicty but their extrachromosomal status has not been assessed |
| pDGB_αD[Flox'd *coaA/bsR*] | Bacterial abxR: kanR; Dicty abxR: bsR; bsR selectable cassette; a similar assembly is successfully selected for in Dicty but excision by Cre recombinase has not been assessed |
| pDGB_αD[Flox'd *coaA/hygR*] | Bacterial abxR: kanR; Dicty abxR: hygR; hygR selectable cassette; a similar assembly is successfully selected for in Dicty but excision by Cre recombinase has not been assessed |
| pDGB_αD[stuffer] | Bacterial abxR: kanR; Dicty abxR: None; MCS used to occupy an assembly position while shuttling other assemblies between GB backbones; the stuffer has been successfully transformed into Dicty |
| pDGB_αDN[*coaA/mCerulean-H2Bv3-HA*] | Bacterial abxR: kanR; Dicty abxR: neoR; mCerulean-H2Bv3-HA overexpression vector; an Ω-level assembly with this unit is validated in Dicty by microscopy |
| pDGB_αE[DdExChr] | Bacterial abxR: kanR; Dicty abxR: None; confers extrachromosomal maintenance in Dicty to any plasmid to which it is added; DdExChr appears in vectors successfully transformed into Dicty but their extrachromosomal status has not been assessed |
| pDGB_αE[stuffer] | Bacterial abxR: kanR; Dicty abxR: None; MCS used to occupy an assembly position while shuttling other assemblies between GB backbones; the stuffer has been successfully transformed into Dicty |
| pDGB_αEN[*coaA/abpC-mIFP*] | Bacterial abxR: kanR; Dicty abxR: neoR; abpC-mIFP overexpression vector; this assembly is not validated in Dicty |
| pDGB_Ω1[*act15/ctsZ-GST; coaA/neoR*] | Bacterial abxR: chlorR; Dicty abxR: neoR; ctsZ-GST overexpression vector; this assembly is not validated in Dicty |
| pDGB_Ω1[*act15/ctsZ-mNeonGreen; coaA/neoR*] | Bacterial abxR: chlorR; Dicty abxR: neoR; ctsZ-mNeonGreen overexpression vector; this assembly is not validated in Dicty |
| pDGB_Ω1[*act15/DDB_G0278295-GST; coaA/neoR*] | Bacterial abxR: chlorR; Dicty abxR: neoR; DDB_G0278295-GST overexpression vector; this assembly is validated in Dicty by Western blot |
| pDGB_Ω1[*act15/DDB_G0278295-mNeonGreen; coaA/neoR*] | Bacterial abxR: chlorR; Dicty abxR: neoR; DDB_G0278295-mNeonGreen overexpression vector; this assembly is not validated in Dicty |
| pDGB_Ω1[*act15/DDB_G0289731-GST; coaA/neoR*] | Bacterial abxR: chlorR; Dicty abxR: neoR; DDB_G0289731-GST overexpression vector; this assembly is validated in Dicty by Western blot |
| pDGB_Ω1[*act15/DDB_G0289731-mNeonGreen; coaA/neoR*] | Bacterial abxR: chlorR; Dicty abxR: neoR; DDB_G0289731-mNeonGreen overexpression vector; this assembly is not validated in Dicty |
| pDGB_Ω1[*act15/gghA-GST; coaA/neoR*] | Bacterial abxR: chlorR; Dicty abxR: neoR; gghα-GST overexpression vector; this assembly is not validated in Dicty |
| pDGB_Ω1[*act15/gghA-mNeonGreen; coaA/neoR*] | Bacterial abxR: chlorR; Dicty abxR: neoR; gghα-mNeonGreen overexpression vector; this assembly is not validated in Dicty |
| pDGB_Ω1[*act15/GST-iliE-1; coaA/neoR*] | Bacterial abxR: chlorR; Dicty abxR: neoR; GST-iliE-1 overexpression vector; this assembly is successfully transformed into Dicty |
| pDGB_Ω1[*act15/mIFP; coaA/neoR*] | Bacterial abxR: chlorR; Dicty abxR: neoR; mIFP overexpression vector; mIFP requires brief exposure to a biliverdin cofactor at 50 µM to fluoresce; this assembly is validated in Dicty by microscopy |
| pDGB_Ω1[*act15/mNeonGreen; coaA/bsR*] | Bacterial abxR: chlorR; Dicty abxR: bsR; mNeonGreen overexpression vector; this assembly is validated in Dicty by microscopy |
| pDGB_Ω1[*act15/mNeonGreen; coaA/hygR*] | Bacterial abxR: chlorR; Dicty abxR: hygR; mNeonGreen overexpression vector; this assembly is validated in Dicty by microscopy |
| pDGB_Ω1[*act15/mNeonGreen; coaA/neoR*] | Bacterial abxR: chlorR; Dicty abxR: neoR; mNeonGreen overexpression vector; this assembly is validated in Dicty by microscopy |
| pDGB_Ω1[*act15/mNeonGreen-ralGDS; coaA/neoR*] | Bacterial abxR: chlorR; Dicty abxR: neoR; mNeonGreen-ralGDS overexpression vector; this assembly is successfully transformed into Dicty |
| pDGB_Ω1[*act15/myc-ralGDS; coaA/neoR*] | Bacterial abxR: chlorR; Dicty abxR: neoR; myc-ralGDS overexpression vector; this assembly is successfully transformed into Dicty |
| pDGB_Ω1[*act15/renilla/act8;* Flox'd *coaA/bsR*] | Bacterial abxR: chlorR; Dicty abxR: bsR; renilla luciferase overexpression vector; although all parts present in this assembly are validated in Dicty in other assemblies, this assembly itself is not validated in Dicty |
| pDGB_Ω1[*act15/renilla/mhcA;* Flox'd *coaA/bsR*] | Bacterial abxR: chlorR; Dicty abxR: bsR; renilla luciferase overexpression vector; an assembly with this unit is validated in Dicty by luciferase reporter assay |
| pDGB_Ω1[*act15/SpCas9-SV40NLS-eGFP;* tRNA-sgRNAtemplate] | Bacterial abxR: chlorR; Dicty abxR: None; intermediate vector for pDGB_A2[CRISPR1] which is validated in Dicty by confirmation of gene editing |
| pDGB_Ω1[*act15/tagBFP; coaA/neoR*] | Bacterial abxR: chlorR; Dicty abxR: neoR; tagBFP overexpression vector; this assembly is validated in Dicty by microscopy |
| pDGB_Ω1[*act15/tagBFP-ralGDS; coaA/neoR*] | Bacterial abxR: chlorR; Dicty abxR: neoR; tagBFP-ralGDS overexpression vector; this assembly is successfully transformed into Dicty |
| pDGB_Ω1[*aurK/sfGFP; coaA/neoR*] | Bacterial abxR: chlorR; Dicty abxR: neoR; sfGFP driven by the putatively light-sensitive aurK promoter; this assembly is validated in Dicty by microscopy |
| pDGB_Ω1[*coaA/bsR; act15/mCherry*] | Bacterial abxR: chlorR; Dicty abxR: bsR; mCherry overexpression vector; this assembly is not validated in Dicty |
| pDGB_Ω1[*coaA/bsR; act15/sfGFP*] | Bacterial abxR: chlorR; Dicty abxR: bsR; sfGFP overexpression vector; although all parts present in this assembly are validated in Dicty in other assemblies, this assembly itself is not validated in Dicty |
| pDGB_Ω1[*coaA/bsR; coaA/mCherry*] | Bacterial abxR: chlorR; Dicty abxR: bsR; mCherry overexpression vector; this assembly is not validated in Dicty |
| pDGB_Ω1[coaA/bsR; stuffer] | Bacterial abxR: chlorR; Dicty abxR: bsR; bsR selectable marker; multiple assemblies that contain this unit are validated in Dicty by successful transformation and drug selection |
| pDGB_Ω1[*coaA/eYFP; coaA/neoR*] | Bacterial abxR: chlorR; Dicty abxR: neoR; eYFP overexpression vector; this assembly is validated in Dicty by microscopy |
| pDGB_Ω1[coaA/hygR; stuffer] | Bacterial abxR: chlorR; Dicty abxR: hygR; hygR selectable marker; an Ω-level assembly with this unit is validated in Dicty by successful transformation and drug selection |
| pDGB_Ω1[*coaA/mCerulean; coaA/neoR*] | Bacterial abxR: chlorR; Dicty abxR: neoR; mCerulean overexpression vector; this assembly is validated in Dicty |
| pDGB_Ω1[coaA/neoR; stuffer] | Bacterial abxR: chlorR; Dicty abxR: neoR; neoR selectable marker; multiple assemblies that contain this unit are validated in Dicty by successful transformation and drug selection |
| pDGB_Ω1[*coaA/renilla/act8;* Flox'd *coaA/bsR*] | Bacterial abxR: chlorR; Dicty abxR: bsR; renilla luciferase overexpression vector; although all parts present in this assembly are validated in Dicty in other assemblies, this assembly itself is not validated in Dicty |
| pDGB_Ω1[*coaA/renilla/mhcA;* Flox'd *coaA/bsR*] | Bacterial abxR: chlorR; Dicty abxR: bsR; renilla luciferase overexpression vector; although all parts present in this assembly are validated in Dicty in other assemblies, this assembly itself is not validated in Dicty |
| pDGB_Ω1[*DDB_G0288429/*  *sfGFP; coaA/neoR*] | Bacterial abxR: chlorR; Dicty abxR: neoR; sfGFP driven by the putatively light-sensitive DDB_G0288429 promoter; this assembly is validated in Dicty by microscopy |
| pDGB_Ω1[DdExChr; stuffer] | Bacterial abxR: chlorR; Dicty abxR: None; confers extrachromosomal maintenance in Dicty to any plasmid to which it is added; DdExChr appears in vectors successfully transformed into Dicty but their extrachromosomal status has not been assessed |
| pDGB_Ω1[*dscA/dendra2; coaA/neoR*] | Bacterial abxR: chlorR; Dicty abxR: neoR; photoswitchable-FP dendra2 driven by dscA promoter; this assembly is not validated in Dicty |
| pDGB_Ω1[*dscA/eYFP; coaA/neoR*] | Bacterial abxR: chlorR; Dicty abxR: neoR; eYFP driven by dscA promoter; this assembly is validated in Dicty by microscopy |
| pDGB_Ω1[*dscA/mCerulean; coaA/neoR*] | Bacterial abxR: chlorR; Dicty abxR: neoR; mCerulean driven by dscA promoter; this assembly is not validated in Dicty |
| pDGB_Ω1[*dscA/mCherry; coaA/neoR*] | Bacterial abxR: chlorR; Dicty abxR: neoR; mCherry driven by dscA promoter; this assembly is not validated in Dicty |
| pDGB_Ω1[stuffer] | Bacterial abxR: chlorR; Dicty abxR: None; MCS used to occupy an assembly position while shuttling other assemblies between GB backbones; the stuffer has been successfully transformed into Dicty |
| pDGB_Ω1[*thyA/sfGFP; coaA/neoR*] | Bacterial abxR: chlorR; Dicty abxR: neoR; sfGFP driven by the putatively light-sensitive thyA promoter; this assembly is validated in Dicty by microscopy |
| pDGB_Ω1N[4-color] | Bacterial abxR: chlorR; Dicty abxR: neoR; contains four biosensors overexpressed from the same backbone (flamindo2, PH-sfGFP, gtaC-mCherry, mCerulean-H2B-HA); this assembly is validated in Dicty by microscopy |
| pDGB_Ω2[*coaA/bsR*; stuffer] | Bacterial abxR: chlorR; Dicty abxR: bsR; bsR selectable marker; multiple assemblies that contain this unit are validated in Dicty by successful transformation and drug selection |
| pDGB_Ω2[*coaA/hygR*; stuffer] | Bacterial abxR: chlorR; Dicty abxR: hygR; hygR selectable marker; an Ω-level assembly with this unit is validated in Dicty by successful transformation and drug selection |
| pDGB_Ω2[*coaA/neoR; coaA/sfGFP*] | Bacterial abxR: chlorR; Dicty abxR: neoR; sfGFP overexpression vector; an assembly with this unit is validated in Dicty by microscopy |
| pDGB_Ω2[*coaA/neoR*; stuffer] | Bacterial abxR: chlorR; Dicty abxR: neoR; neoR selectable marker; multiple assemblies that contain this unit are validated in Dicty by successful transformation and drug selection |
| pDGB_Ω2[DdExChr; stuffer] | Bacterial abxR: chlorR; Dicty abxR: None; confers extrachromosomal maintenance in Dicty to any plasmid to which it is added; DdExChr appears in vectors successfully transformed into Dicty but their extrachromosomal status has not been assessed |
| pDGB_Ω2[stuffer] | Bacterial abxR: chlorR; Dicty abxR: None; MCS used to occupy an assembly position while shuttling other assemblies between GB backbones; the stuffer has been successfully transformed into Dicty |

a: abxR = antibiotic resistance

Supplementary Table 3 | DNA primer sequences

| **Primer ID** | **Sequence (5’ to 3’)** |
| --- | --- |
| PNK0215_bsR_152-172_seq_rev | CAGTTACTCGTCCTATATACG |
| PNK0216_bsR_50-79_seq_rev | CATTGTAATCTTCTCTGTCGCTACTTCTAC |
| PNK0217_bsR_306-335_seq_fwd | GTGTAGGGAGTTGATTTCAGACTATGCACC |
| PNK0218_bsR_276-298_seq_fwd | AGTATTCGAGTGGTAAGTCCTTG |
| PNK0219_bsR_313-335_seq_rev | GGTGCATAGTCTGAAATCAACTC |
| PNK0319_act15p_GGAG | GCGCCGTCTCACTCGGGAGTCTAGATAAAAAAAATTTTTATTTATTTTTATTTATTTTG |
| PNK0320_act15p_AATG | GCGCCGTCTCACTCGCATTTTTATTTTTTTATTTAATTTAATTTATTTGTTTTAAG |
| PNK0321_act8t_GCTT | GCGCCGTCTCACTCGGCTTGCTAGAGTCGTCCATCAATTGTTC |
| PNK0322_act8t_CGCT | GCGCCGTCTCACTCGAGCGTTTCGGGTCAGCTTTATCTTTTTG |
| PNK0327_ecmA_GGAG | GCGCCGTCTCACTCGGGAGTCATGGTAAAACAAATTGGTATTTGTT |
| PNK0328_ecmA_AATG | GCGCCGTCTCACTCGCATTTTTCAACGTTATAATTTTTAAACTAATGAT |
| PNK0331_mNeonGreen_AATG | GCGCCGTCTCACTCGAATGATGGTTAGTAAAGGTGAAGAAGATAATATG |
| PNK0332_mNeonGreen_GCTT | GCGCCGTCTCACTCGAAGCTTACTTGTACAGCTCATCCATGCC |
| PNK0341_bsR_AATG | GCGCCGTCTCACTCGAATGATGGATCAATTTAACATTTCTCAACAAG |
| PNK0342_bsR_GCTT | GCGCCGTCTCACTCGAAGCTTAATTTCGGGTATATTTGAGTGGAATG |
| PNK0343_hygR_AATG | GCGCCGTCTCACTCGAATGATGGATCAATTTAACATTTCTCAACAAG |
| PNK0344_hygR_GCTT | GCGCCGTCTCACTCGAAGCTTAGTTAGCCTCCCCCATCTCC |
| PNK0345_neoR_AATG | GCGCCGTCTCACTCGAATGATGATTGAACAAGATGGATTGCACG |
| PNK0346_neoR_GCTT | GCGCCGTCTCACTCGAAGCTTAGAAGAACTCGTCAAGAAGG |
| PNK0347_mhcAt_GCTT | GCGCCGTCTCACTCGGCTTATCAATTTGATTTCTTCTTAATTCAATGCTAA |
| PNK0348_mhcAt_CGCT | GCGCCGTCTCACTCGAGCGCATTTTATTTAATATACTAAATAATAAAAAAGTTAAAAAAT |
| PNK0349_actin6p_GGAG | GCGCCGTCTCACTCGGGAGTTTTTAAATAAAAAATGGGTTTTTTTTAAGTAAAGTTAT |
| PNK0350_actin6p_AATG | GCGCCGTCTCACTCGCATTTATATTATATTTATTTATTGATTATTTTTTTGAATTAATTA |
| PNK0351_tgrBCp_GGAG | GCGCCGTCTCACTCGGGAGTTTTATCAATGATTTTTTTTATTTTGAATTTGGC |
| PNK0352_tgrBCp_AATG | GCGCCGTCTCACTCGCATTTTTTGTAAAACTTGTAGCATTATAAATTTAAAC |
| PNK0353_coaAp_GGAG | GCGCCGTCTCACTCGGGAGCAAAATTTTGTATTTTGTGATGTTAGTGTG |
| PNK0354_coaAp_AATG | GCGCCGTCTCACTCGCATTTGTGAAATTAGTTTAAAATACAAATAAAGAGTTATA |
| PNK0361_hygR_patch1_rev | GCGCCGTCTCGATACGCTGTCGAACTTTTCGA |
| PNK0362_hygR_patch2_fwd | GCGCCGTCTCGGTATCCGACCTGATGCAGCT |
| PNK0363_hygR_patch2_rev | GCGCCGTCTCGTTGCCTCGCTCCAGTCAATG |
| PNK0364_hygR_patch2_fwd | GCGCCGTCTCGGCAATGTTCGGGGATTCCCA |
| PNK0365_neoR_patch1_rev | GCGCCGTCTCGTTCGATGTTTCGCTTGGTGG |
| PNK0366_neoR_patch2_fwd | GCGCCGTCTCGGTATCCGACCTGATGCAGCT |
| PNK0367_neoR_patch2_rev | GCGCCGTCTCGTTGCCTCGCTCCAGTCAATG |
| PNK0368_neoR_patch3_fwd | GCGCCGTCTCGGCAATGTTCGGGGATTCCCA |
| PNK0373_loxP_coaAp_GGAG | GCGCCGTCTCACTCGGGAGATAACTTCGTATAGCATACATTATACGAAGTTATCAAAATTTTGTATTTTGTGATGTTAGTGTG |
| PNK0375_mhcAt_loxP_CGCT | GCGCCGTCTCACTCGAGCGATAACTTCGTATAATGTATGCTATACGAAGTTATCATTTTATTTAATATACTAAATAATAAAAAAGTTAAA |
| PNK0380_GST_AATG | GCGCCGTCTCACTCGAATGGATGGTACATCACCTATCCTCG |
| PNK0381_GST_AGTG | GCGCCGTCTCACTCGCACTTGAACCACGCGGAACCAG |
| PNK0382_GST_CTCG | GCGCCGTCTCACTCGCTCGCTGGTTCCGCGTGGTTCAGATGGTACATCACCTATCCTCG |
| PNK0383_GST_GCTT | GCGCCGTCTCACTCGAAGCTTAATCCGATTTTGGAGGATGGTC |
| PNK0390_DDB_G0278295_AATG | GCGCCGTCTCACTCGAATGAGATTATTATTAGCCCTCTTTTTCGTTCTTGC |
| PNK0391_DDB_G0278295_TCGG | GCGCCGTCTCACTCGCCGACAAGTTAATATCAACATCAATACAGGCGATTTCAG |
| PNK0392_DDB_G0289731_AATG | GCGCCGTCTCACTCGAATGGCAGAAGTTAGTTTTAAAGAAAGTGATATTTTAAAAAAATA |
| PNK0393_DDB_G0289731_TCGG | GCGCCGTCTCACTCGCCGATTCTAATCTATAGTGAACACAATTAAGTTGATTCATATATG |
| PNK0394_gghA_fwd_AATG | GCGCCGTCTCACTCGAATGAATAAATTAATTGTAGTAATAATTTCAATCATTTTAATGGT |
| PNK0395_gghA_rev_TCGG | GCGCCGTCTCACTCGCCGAATTAAAATAATAAATTTGTTCAAAATCTGGTACAGTTGATT |
| PNK0404_GGGSx4linker_AGTGGC* | GCGCCGTCTCACTCGAGTGGCGGTGGAGGTTCAGGAGGTGGAAGTGGTGGAGGTTCAGGAGGTGGTTCAGGTAGCCGAGTGAGACGCGCG |
| PNK0405_GGGSx4linker_TCGGGC* | GCGCCGTCTCACTCGTCGGGCGGTGGAGGTTCAGGAGGTGGAAGTGGTGGAGGTTCAGGAGGTGGTTCAGGCTCGCGAGTGAGACGCGCG |
| PNK0410_mNeonGreen_AGTG | GCGCCGTCTCACTCGCACTCTTGTACAGCTCATCCATGCC |
| PNK0411_mNeonGreen_CTCG | GCGCCGTCTCACTCGCTCGATGGTTAGTAAAGGTGAAGAAGATAATATG |
| PNK0414_gghA_patch1_rev | GCGCCGTCTCGGCGACCAAATCGATTGGATG |
| PNK0415_gghA_patch2_fwd | GCGCCGTCTCGTCGCAAGTAGTAGAGTTTAACAAC |
| PNK0431_HA_AATG_AGTG* | GCGCCGTCTCACTCGAATGTATCCATATGATGTTCCAGATTATGCTAGTGCGAGTGAGACGGCGC |
| PNK0432_HA_CTCG_GCTT* | GCGCCGTCTCACTCGCTCGTATCCATATGATGTTCCAGATTATGCTTAAGCTTCGAGTGAGACGGCGC |
| PNK0463_tagBFP_AATG | GCGCCGTCTCACTCGAATGAGTGAACTTATTAAAGAAAATATGCATATGAAACTTTACATGGAGGGCACCGTGG |
| PNK0464_tagBFP_AGTG | GCGCCGTCTCACTCGCACTATTAAGCTTGTGCCCCAGTTTGCTAG |
| PNK0465_mIFP_AATG | GCGCCGTCTCACTCGAATGTCAGTTCCATTAACAACATCAGCATTTGGTCATGCTTTCCTGGCCAACTG |
| PNK0466_mIFP_AGTG | GCGCCGTCTCACTCGCACTTTTGGACTGGCTCTGGGCGAAG |
| PNK0467_tagBFP_CTCG | GCGCCGTCTCACTCGCTCGAGTGAACTTATTAAAGAAAATATGCATATGAAACTTTAC |
| PNK0468_tagBFP_GCTT | GCGCCGTCTCACTCGAAGCTTAATTAAGCTTGTGCCCCAGTTTG |
| PNK0469_mIFP_CTCG | GCGCCGTCTCACTCGCTCGTCAGTTCCATTAACAACATCAGCATTTGG |
| PNK0470_mIFP_GCTT | GCGCCGTCTCACTCGAAGCTTATTTGGACTGGCTCTGGGCGAAG |
| PNK0471_sfGFP_AATG | GCGCCGTCTCACTCGAATGAGAAAAGGTGAAGAATTATTCACCGG |
| PNK0472_sfGFP_AGTG | GCGCCGTCTCACTCGCACTTTTGTACAGTTCATCCATGCCCAGGGTG |
| PNK0473_sfGFP_CTCG | GCGCCGTCTCACTCGCTCGAGAAAAGGTGAAGAATTATTCACCGG |
| PNK0474_sfGFP_TAAGCTT | GCGCCGTCTCACTCGAAGCTTATTTGTACAGTTCATCCATGCCCAGGGTG |
| PNK0475_Myc_AATG_AGTG_top* | GCGCCGTCTCACTCGAATGGAACAAAAACTTATTTCAGAAGAAGATCTTAGTGCGAGTGAGACGGCGC |
| PNK0476_Myc_AATG_AGTG_bot* | GCGCCGTCTCACTCGCACTAAGATCTTCTTCTGAAATAAGTTTTTGTTCCATTCGAGTGAGACGGCGC |
| PNK0477_Myc_CTCG_TAAGCTT_top* | GCGCCGTCTCACTCGCTCGGAACAAAAACTTATTTCAGAAGAAGATCTTTAAGCTTCGAGTGAGACGGCGC |
| PNK0478_Myc_CTCG_TAAGCTT_bot* | GCGCCGTCTCACTCGAAGCTTAAAGATCTTCTTCTGAAATAAGTTTTTGTTCCGAGCGAGTGAGACGGCGC |
| PNK0479_DdExChr_GGAG | GCGCCGTCTCACTCGGGAGATCGATTTCGAAAAAGTAAATAGCAATTATTAC |
| PNK0480_DdExChr_CGCT | GCGCCGTCTCACTCGAGCGCTCGACATTTGAAATTACAATGAATGTTTTTC |
| PNK0481_FfLuc_AATG | GCGCCGTCTCACTCGAATGATGGAAGATGCAAAAAATATTAAAAAAGGTCCGGCGCCATTCTATCCG |
| PNK0482_FfLuc_GCTT | GCGCCGTCTCACTCGAAGCTTACACGGCGATCTTTCCGCC |
| PNK0483_renilla_AATG | GCGCCGTCTCACTCGAATGATGACATCAAAAGTTTATGATCCAGAACAACGCAAGAGAATGATAACTGGGCC |
| PNK0484_renilla_GCTT | GCGCCGTCTCACTCGAAGCTTACTGTTCGTTCTTCAGGACCCTCTC |
| PNK0496_ralGDS_TAGC | GCGCCGTCTCACTCGTAGCATGAGCTCCGCGCTGCCG |
| PNK0498_ralGDS_GCTT | GCGCCGTCTCACTCGAAGCTTACCGCTTCTTGAGGACAAAGTCATAG |
| PNK0508_H2Bv3_TAGC | GCGCCGTCTCACTCGTAGCATGGTATTCGTTAAAGGTCAAAAGAAAGCAAC |
| PNK0509_H2Bv3_TCGG | GCGCCGTCTCACTCGCCGAGTTTTTGCTTTCAGTTGGATTGTACTTGTTG |
| PNK0511_iliE-1_TAGC | GCGCCGTCTCACTCGTAGCATGGTTAAAATTGCAACAAGAAGTTCAAC |
| PNK0513_iliE-1_GCTT | GCGCCGTCTCACTCGAAGCTTATTGATTTAAGGTTAATTTACAATTTCTAATTGACTGTT |
| PNK0514_Flexlink_AGTGGC_top* | GCGCCGTCTCACTCGAGTGGCGGTTCAGCAGGTTCAGCAGCAGGTTCAGGTGAATTTGGTAGCCGAGTGAGACGCGCG |
| PNK0515_Flexlink_AGTGGC_bot* | CGCGCGTCTCACTCGGCTACCAAATTCACCTGAACCTGCTGCTGAACCTGCTGAACCGCCACTCGAGTGAGACGGCGC |
| PNK0516_Flexlink_TCGGGC_top* | GCGCCGTCTCACTCGTCGGGCGGTTCAGCAGGTTCAGCAGCAGGTTCAGGTGAATTTGGCTCGCGAGTGAGACGCGCG |
| PNK0517_Flexlink_TCGGGC_bot* | CGCGCGTCTCACTCGCGAGCCAAATTCACCTGAACCTGCTGCTGAACCTGCTGAACCGCCCGACGAGTGAGACGGCGC |
| PNK0524_dendra2_AATG | GCGCCGTCTCACTCGAATGAATCTTATTAAAGAAGATATGCGTGTTAAAG |
| PNK0525_dendra2_AGTG | GCGCCGTCTCACTCGCACTCCAAACTTGACTTGGTAGTGG |
| PNK0526_dendra2_CTCG | GCGCCGTCTCACTCGCTCGATGAATCTTATTAAAGAAGATATGCGTGTTAAAG |
| PNK0527_dendra2_GCTT | GCGCCGTCTCACTCGAAGCTTACCAAACTTGACTTGGTAGTGG |
| PNK0528_mCherry_AATG | GCGCCGTCTCACTCGAATGGTTTCAAAAGGTGAAGAAGATAATATGG |
| PNK0529_mCherry_AGTG | GCGCCGTCTCACTCGCACTTTTATATAATTCATCCATACCACCTGTTGAATG |
| PNK0530_mCherry_CTCG | GCGCCGTCTCACTCGCTCGATGGTTTCAAAAGGTGAAGAAGATAATATGG |
| PNK0531_mCherry_TAAGCTT | GCGCCGTCTCACTCGAAGCTTATTTATATAATTCATCCATACCACCTGTTGAATG |
| PNK0532_7xHis_AATG_AGTG_top | GCGCCGTCTCACTCGAATGCATCATCATCATCATCATCACAGTGCGAGTGAGACGGCGC |
| PNK0533_7xHis_AATG_AGTG_bottom | GCGCCGTCTCACTCGCACTGTGATGATGATGATGATGATGCATTCGAGTGAGACGGCGC |
| PNK0534_7xHis_CTCG_TAAGCTT_top | CGCCGTCTCACTCGCTCGCATCATCATCATCATCATCACTAAGCTTCGAGTGAGACGGCG |
| PNK0535_7xHis_CTCG_TAAGCTT_bottom | CGCCGTCTCACTCGAAGCTTAGTGATGATGATGATGATGATGCGAGCGAGTGAGACGGCG |
| PNK0536_tRNA_sgRNA_GGAG | GCGCCGTCTCACTCGGGAGGCTCGATTAGCTCAGTCGGCAG |
| PNK0537_tRNA_sgRNA_GCTT | GCGCCGTCTCACTCGAAGCAAAAAAAGCACCGACTCGGTG |
| PNK0542_Cas9_rev_seq | TTGCCATTTCGTTGGAGAATATCTCTTG |
| PNK0543_tagBFP_AATG | GCGCCGTCTCACTCGAATGAGTGAACTTATTAAAGAAAATATGCATATGAAACTTTAC |
| PNK0544_tagBFP_GGTAGC | GCGCCGTCTCACTCGGCTACCATTAAGCTTGTGCCCCAGTTTG |
| PNK0546_mNeonGreen_GGTAGC | GCGCCGTCTCACTCGGCTACCCTTGTACAGCTCATCCATGCC |
| PNK0547_Cas9-SV40NLS-eGFP_AATG | GCGCCGTCTCACTCGAATGGATAAAAAATATTCAATTGGTTTAGATATTG |
| PNK0548_Cas9-SV40NLS-eGFP_GCTT | GCGCCGTCTCACTCGAAGCTTATTTGTATAGTTCATCCATGCCATGTG |
| PNK0549_Cas9_seq_649_fwd | TGTCAAAGAGCCGCAGACTTG |
| PNK0550_Cas9_seq_GFP_rev | CACCCTCTCCACTGACAGAAAATTTGTG |
| PNK0551_Cas9_seq_3660_rev | AGAGCCAGCTCATTACCCTTCTGAAG |
| PNK0552_Cas9_seq_1220_fwd | ATGGGAGCATTCCCCACCAG |
| PNK0553_Cas9_seq_1851_fwd | GGACATTGTCCTGACTCTCACTCTG |
| PNK0554_Cas9_seq_3025_rev | AGACCTTGTAGTCTCCGTACACGA |
| PNK0555_Cas9_seq_2370_rev | TGCTCTTTAAGGATCTGTGACCCGAG |
| PNK0556_Cas9_patch1_rev | GCGCCGTCTCGCTGTCTCCCCTGAATCAAAG |
| PNK0557_Cas9_patch2_fwd | GCGCCGTCTCGACAGCTGAAGCAACCCGCCT |
| PNK0558_Cas9_patch2_rev | GCGCCGTCTCGTGATCTCTCTTTTCGTTGGGA |
| PNK0559_Cas9_patch3_fwd | GCGCCGTCTCGATCACATGGTCCTTCTTGAGT |
| PNK0560_Cas9_seq_1128_rev | GTCCATCTTCTCAAGGATAGGTTTG |
| PNK0561_HypaCas9_patch1_fwd | GCGCCGTCTCACTCGAATGGATAAAAAGTACAGTATAGGTTTGGATATTG |
| PNK0562_HypaCas9_patch1_rev | GCGCCGTCTCACCTCTGAACTTAATCATGTGTG |
| PNK0563_HypaCas9_patch2_fwd | GCGCCGTCTCAGAGGTCATTTTTTGATTGAGGGAG |
| PNK0564_HypaCas9_patch2_rev | GCGCCGTCTCGGCCTTCTCAAAATAGCATGCA |
| PNK0565_HypaCas9_patch3_fwd | GCGCCGTCTCGAGGCAAGAAGATTTTTATCCATTTTT |
| PNK0566_HypaCas9_patch3_rev | GCGCCGTCTCGTATCTTCTTTAAATGTCAAACTATCATC |
| PNK0567_Cas9_patch4_fwd | GCGCCGTCTCGGATATTCAAAAAGCACAAGTGTCT |
| PNK0568_Cas9_patch4_rev | GCGCCGTCTCACTCGCCGATTAGTCACCTCCTAGCTGACTCAAATCAATG |
| PNK0569_HypaCas9_DCO_5’ fragment* | ATGGATAAAAAGTACAGTATAGGTTTGGATATTGGTACCAATAGTGTTGGTTGGGCAGTAATAACCGATGAATATAAAGTTCCTAGTAAAAAGTTCAAGGTACTTGGTAATACCGATAGACACAGTATTAAAAAGAATTTAATAGGTGCCTTATTGTTCGATAGTGGAGAGACTGCTGAAGCAACTCGTTTAAAGCGTACAGCAAGAAGAAGATATACAAGACGTAAAAATAGAATATGTTATTTACAAGAGATTTTTTCAAATGAAATGGCAAAAGTTGATGATTCATTTTTTCATAGATTAGAAGAATCATTTTTAGTTGAAGAAGATAAAAAACATGAAAGACATCCAATTTTTGGTAATATTGTTGATGAAGTTGCATATCATGAAAAATATCCAACAATTTATCATTTAAGAAAAAAATTAGTTGATTCAACAGATAAAGCAGATTTAAGATTAATTTATTTAGCATTAGCACACATGATTAAGTTCAGAGGT |
| PNK0570_sgRNA_seq_fwd | GGAGGCTCGATTAGCTCAGTCGGCAG |
| PNK0571_sgRNA_seq_rev | CAAGTTGATAACGGACTAGCCTT |
| PNK0577_thyAp_GGAG | GCGCCGTCTCACTCGGGAGTTATCTGGTAAAATGAAAGTATCAATAGTTTAACC |
| PNK0578_thyAp_AATG | GCGCCGTCTCACTCGCATTTTATCGATTTCTGTTTGAATATCAAGACCCAT |
| PNK0579_aurKp_GGAG | GCGCCGTCTCACTCGGGAGAAGAAAACAATTCTTAGAAACTATGAGTG |
| PNK0580_aurKp_AATG | GCGCCGTCTCACTCGCATTTCTTTATTATTTGGATAACTCATTTCTTTAATTT |
| PNK0603_DDB_G0288429p_fwd | GCGCCGTCTCACTCGGGAGCTGGACGTGACACTTAGC |
| PNK0604_DDB_G0288429p_rev | GCGCCGTCTCACTCGCATTCCATTCCAAAACCATTTTGACG |
| PNK0611_eYFP_mCer_AATG | GCGCCGTCTCACTCGAATGTCAAAAGGTGAAGAATTATTTACAGGTGTGGTGCCCATCCT |
| PNK0612_eYFP_mCer_GCTT | GCGCCGTCTCACTCGAAGCTTACTTGTACAGCTCGTCCATG |
| PNK0613_eYFP_mCer_CTCG | GCGCCGTCTCACTCGCTCGATGTCAAAAGGTGAAGAATTATTTACAGGTGTGGTGCCCAT |
| PNK0614_eYFP_mCer_AGTG | CGCCGTCTCACTCGCACTCTTGTACAGCTCGTCCATG |
| PNK0615_abpC_AATG | GCGCCGTCTCACTCGAATGGCTGCTGCTCCAAGTG |
| PNK0616_abpC_GCTT | GCGCCGTCTCACTCGAAGCTTATTAATTGGCAGTACGAGTAGTAGTGAAAG |
| PNK0617_abpC_TAGC | GCGCCGTCTCACTCGTAGCATGGCTGCTGCTCCAAGTG |
| PNK0619_HypaCas9_648_seq | CTAAAGCGATTCTTTCTGCACGATTG |
| PNK0620_HypaCas9_1400_seq | CAATAGTCGTTTTGCATGGATGACTCG |
| PNK0621_HypaCas9_2190 | ACATATTGCAAATTTAGCTGGTAGCCCTG |
| PNK0622_HypaCas9_3400_seq | ACCACTAGGACTGAATAAGCTACCG |
| PNK0623_HypaCas9_2660_seq | ACGTTGAGTGATTAACTTGGCGTTTAG |
| PNK0624_Barcode_CAGT | CGCGCGTCTCACTCGCAGTATGGCAAGCGCTCGACGAATTAAC |
| PNK0625_Barcode_CGCT | CGCGCGTCTCACTCGAGCGGTTCTCCTTGTTCTCCACGGTCTT |
| PNK0626_DdOri_SDM_fwd | GAGTGGAAGATAGCTTTGTTG |
| PNK0627_DdOri_SDM_rev | TAAACAAATAACCATCATCAG |
| PNK0628_PHdagA_AATG | GCGCCGTCTCACTCGAATGGGGAAAACAGAGAGAAAGAAAG |
| PNK0629_PhdagA_TCGG | GCGCCGTCTCACTCGCCGATTTTTCAAGTGATCTAATTCTTGCGATGAG |
| PNK0634_gtaC_AATG | GCGCCGTCTCACTCGAATGAATCATCAATATATACCATCTCCAATT |
| PNK0636_HypaCas9_seq_3900 | ATAGAGACAAACCAATACGTGAACAAG |
| PNK0637_Cas9_seq_1380_rev | CAAGCAAACCGGCTATTTCCTC |
| PNK0646_dscAp_GGAG | GCGCCGTCTCACTCGGGAGTACCACCTCCACCTATTCAAATTCC |
| PNK0647_dscAp_AATG | GCGCCGTCTCACTCGCATTTTTATTTGTGTTGAATTTAATATTTAAAAGATAATTTG |
| PNK0650_acaA_sgRNA_sense | AGAAGACGGAGCATCGATCACACAACAAAGGGGGTTTCCGTCTTCT |
| PNK0651_acaA_sgRNA_antisense | AGAAGACGGAAACCCCCTTTGTTGTGTGATCGATGCTCCGTCTTCT |
| PNK0652_rapgapB_sgRNA_2_sense | AGAAGACGGAGCATAACTGATGGAGAAACAAATGTTTCCGTCTTCT |
| PNK0653_rapgapB_sgRNA__2_antisense | AGAAGACGGAAACATTTGTTTCTCCATCAGTTATGCTCCGTCTTCT |
| PNK0658_abpC_TCGG | GCGCCGTCTCACTCGCCGAGGCAGTACGAGTAGTAGTGAAAG |
| PNK0659_gtaC_TCGG | GCGCCGTCTCACTCGCCGAATCGCTAATTAATTTTGAAACACTCATTGC |
| PNK0664_gtaC_seq_669_fwd | GCTTCTTCATCTCATAATAATGATCATCG |
| PNK0665_dscA_AATG | GCGCCGTCTCACTCGAATGTCTACCCAAGGTTTAGTTCAAC |
| PNK0666_dscA_TAGC | GCGCCGTCTCACTCGTAGCATGTCTACCCAAGGTTTAGTTCAAC |
| PNK0667_dscA_TCGG | GCGCCGTCTCACTCGCCGATTCCAAAGCGGTAGCAATGTAATCAG |
| PNK0668_dscA_GCTT | GCGCCGTCTCACTCGAAGCTTATTCCAAAGCGGTAGCAATGTAATCAG |
| PNK0669_coaA_GBEZ_fwd | CGCGGAAGACTCTGTTCAAAATTTTGTATTTTGTGATGTTAGTGTG |
| PNK0670_mhcA_GBEZ_rev | GCGCGAAGACTCGTTACATTTTATTTAATATACTAAATAATAAAAAAGTTAAAAAAT |
| PNK0671_hygR_seq_fwd_516 | TCACTGGCAAACTGTGATGGACG |
| PNK0672_hygR_seq_rev_684 | CAATGACCGCTGTTATGCGG |
| PNK0673_pcat-abxR-t_fwd | GCGCGAAGACTCGTGATGATCGGCACGTAAGAGGTTCC |
| PNK0674_pcat-abxR-t_rev | GCGCGAAGACTCAACATCATGTTTGACAGCTTATCATCGAATTTCTG |
| PNK0675_GBEZ_seq | TTTGCTCACATGTTCTTTCCTGCG |
| PNK0676_hcpB_TAGC | GCGCCGTCTCACTCGTAGCATGGGAAAAAGAGATAAAAAAATAATAATAGAAG |
| PNK0677_hcpB_GCTT | GCGCCGTCTCACTCGAAGCTTAACTTGGCTGACCACTATAACC |
| PNK0678_flamindo2_AATG | GCGCCGTCTCACTCGAATGGTTTCAAAAGGTGAAGAATTATTTACAGGGGTGGTGCCCAT |
| PNK0679_flamindo2_GCTT | GCGCCGTCTCACTCGAAGCTTACTTGTACAGCTCGTCCATGC |
| PNK0680_pink_flamindo_AATG | GCCGTCTCACTCGAATGTTAGTTTCAAAAGGTGAAGAAGATAATATGGCCATCATCAAGG |
| PNK0681_pink_flamindo_GCTT | GCGCCGTCTCACTCGAAGCTTACTTGTACAGCTCGTCCATG |
| PNK0682_act8t_CAGT | GCGCCGTCTCACTCGACTGTTTCGGGTCAGCTTTATCTTTTTG |
| PNK0683_mhcAt_CAGT | GCGCCGTCTCACTCGACTGCATTTTATTTAATATACTAAATAATAAAAAAGTTAAAAAAT |
| PNK0693_iliE-1p_GGAG | GCGCCGTCTCACTCGGGAGTCTATTTTTTGTCGTTAGGACCTAATTTCA |
| PNK0694_iliE-1p_AATG | GCGCCGTCTCACTCGCATTTTTGTTTTTTTTTTTATTTTTATTTTTTTATTAATATATT |
| PNK0695_iliE-1p+7aa_AATG | GCGCCGTCTCACTCGCATTCTTGTTGCAATTTTAACCATTTTG |
| PNK0696_neoR_seq_fwd | GGCTATGACTGGGCACAACAGACAAT |
| PNK0697_neoR_seq_rev | AGAAGAACTCGTCAAGAAGGCG |

* denotes oligos that were hybridized as parts or patches without PCR amplification

**Supplementary Table 4 | Clonal *Dictyostelium* strains generated in this study.**

| ***Dictyostelium* strain** | **Drug resistance** | **Clone #** | **Notes** |
| --- | --- | --- | --- |
| *act15/mIFP* | NeoR | 1 | Integrating vector |
| *act15/tagBFP* | NeoR | 2 | Integrating vector |
| *4-color* | NeoR | 1 | Integrating vector |
| *coaA/flamindo2* | NeoR | 1 | Integrating vector |
| *coaA/pinkflamindo* | NeoR | 1 | Integrating vector |
| *rapgapB^–^* | None | 1 | Generated with pDGB_A2_CRISPR1_rapgapBsgRNA2 |
| *rapgapB^–^* | None | 2 | Generated with pDGB_A2_CRISPR1_rapgapBsgRNA2 |
| *rapgapB^–^* | None | 3 | Generated with pDGB_A2_CRISPR1_rapgapBsgRNA2 |
| *thyA/sfGFP* | NeoR | 1 | Integrating vector |
| *aurK/sfGFP* | NeoR | 1 | Integrating vector |
| *DDB_G0288429/sfGFP* | NeoR | 1 | Integrating vector |
