# Supplementary material for: A GoldenBraid cloning system for synthetic biology in social amoebae
Source: Nucleic Acids Res. 2020 Mar 30;48(8):4139–46. doi: 10.1093/nar/gkaa185 (PMC7192589; doi:10.1093/nar/gkaa185)
Supplement: gkaa185_Supplemental_Files [file gkaa185_supplemental_files.zip › Kundert Supplementary protocols revised.docx]

Supplementary Protocols

Ordering GoldenBraid plasmids from the Dicty Stock Center (DSC)

| **I want to…** | **…so I should order from the DSC:** |
| --- | --- |
| Work with my own DNA parts of interest (e.g. my promoter or CDS of interest) in the GoldenBraid cloning system, i.e. domesticate one or more parts. | 1. pUPD2 backbone 2. The other parts and assembly level backbones necessary for your application, which are detailed below. |
| Build a single transcriptional unit out of domesticated parts, transform it into Dicty, and select for its random integration into the Dicty genome. | 1. pDGB_α1B, -H, and/or -N backbone(s) depending on which drug(s) you want to select with: B-BSR, H-Hygromycin, N-G418 (Neomycin). You can build your transcriptional unit in any α-level backbone with any built-in drug selection. However, if you do not have any pre-existing plans to combine your unit with other units into single backbones, the position in the grammar that leaves you the most options for further assembly reactions, should they arise, is α1. α1 is present in both the standard and expanded α-to-Ω assembly grammars, i.e. you could add 1-4 additional units to your unit in α1 in a single cloning reaction if that becomes necessary. Whereas if you use α2, you could only add one unit in a single cloning reaction because the α2 position is not present in the expanded α-to-Ω assembly grammar. And if you use αB, αC, αD, or αE, you must perform an expanded α-to-Ω assembly reaction to combine your unit with other units. 2. All of the necessary domesticated parts in the pUPD2 backbone. The most 5' grammar of the most 5' part should be GGAG, and the most 3' grammar of the most 3' part should be CGCT in order to ligate into any α-level assembly backbone using *Bsa*I. For parts located internally within the transcriptional unit, the 3' grammar of each upstream part must match the 5' grammar of the next downstream part. |
| Combine multiple transcriptional units into single backbones. | 1. pDGB_α-level backbones 2. At least one pDGB_Ω-level backbone 3. Stuffers as necessary The particular backbones depend on whether you want to combine 2 units in a standard α-to-Ω (α1 and α2) assembly reaction or 5 units in an expanded α-to-Ω assembly reaction (α1, αB, αC, αD, αE). You will need at least one Ω1- and one Ω2- level backbone if you want to perform an Ω-to-α assembly reaction. See the following protocols for more information about stuffers. |
| Build an extrachromosomal GoldenBraid vector. | 1. pDGB_αX_DdExChr, where "X" is an appropriate backbone to combine with your unit(s) of interest.  2. pDGB_Ω#Y, where "#" can be either 1 or 2, and "Y" is your desired selectable drug resistance (usually neoR or hygR for extrachromosomal vectors). For example, if you plan to make an integrating expression vector in the α1N backbone, then you want to put your transcriptional unit into an extrachromosomal backbone with G418 resistance, you should order pDGB_α2_DdExChr and pDGB_Ω1N or pDGB_Ω2N. You would then run a standard α-to-Ω assembly reaction to combine α1N-your unit and α2-DdExChr into the Ω-level backbone. For more complex and custom builds, the DdExChr unit is available in every α- and Ω-level position. |
| Do CRISPR gene editing in the GoldenBraid cloning system. | 1. pDGB_α2[CRISPR1]. This plasmid's editing activity is validated in Dicty. This plasmid is very similar to pTM1285, and uses the same *Bpi*I or *Bbs*I restriction sites as pTM1285 to ligate in the specific sgRNA targeting sequence in a Golden Gate cloning reaction. However, it has two advantages over pTM1285. The first is that it has a *lacZ* cassette that can be used for blue-white screening of bacterial clones that harbor parental plasmid (blue) versus desired sgRNA-target containing plasmid (white). The second is that it can be combined with other assemblies easily as more sophisticated variations of CRISPR technology are implemented in Dicty. |

Protocol 1: Domestication of parts for use in the GoldenBraid cloning system

The first step to make a new genetic part compatible for use in the GoldenBraid cloning system is called part domestication. This encompasses:

1. PCR amplification of the part with addition of type IIS enzyme sites and grammatical bases from 5’ and 3’ nonhomologous primer extensions,
2. Removal of interfering type IIS enzyme recognition sites within the part by PCR mutagenesis,
3. A combined reaction (restriction digestion and ligation) to insert the part fragment(s) into the domestication-level backbone called pUPD2,
4. Part validation by diagnostic restriction digestion and Sanger sequencing.
5. Design primers to amplify your genetic part(s) of interest with 4 base-pair, part-level grammatical overhangs. The grammar specifies the type of part, as shown here:


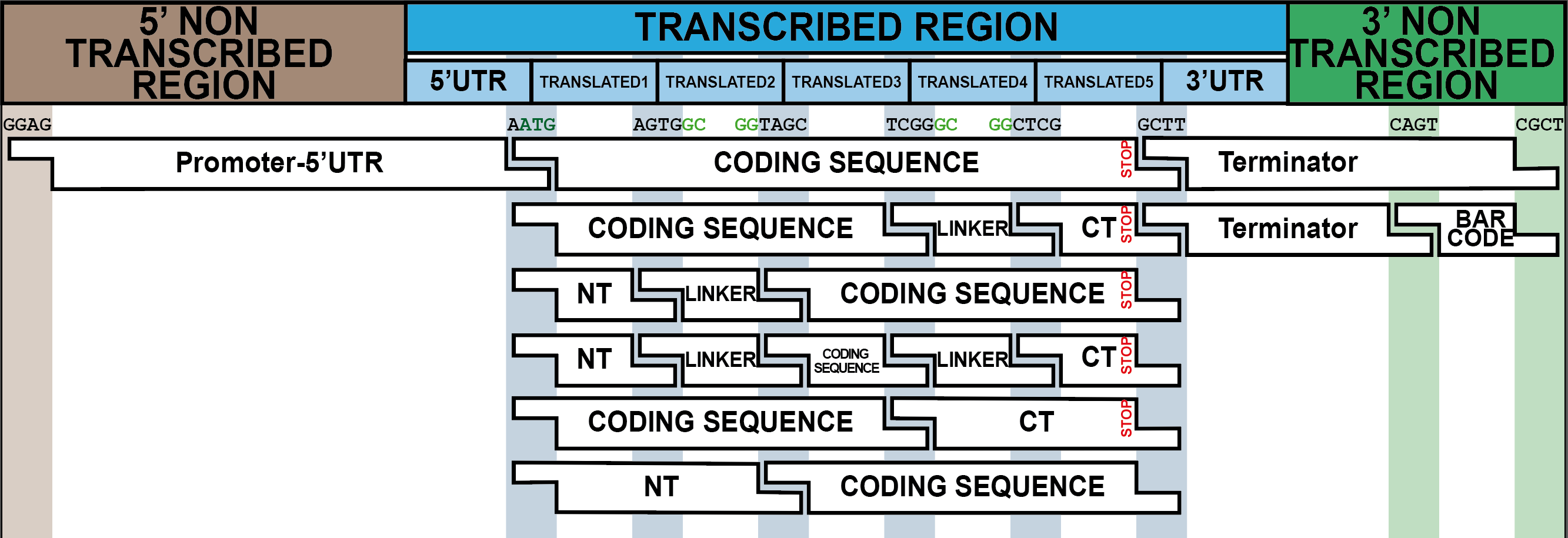


Note 1: The extra bases shown in green throughout the coding sequence grammar are to preserve the correct reading frame when inserting coding elements. Include these additional two bases only in linkers or linker equivalents (e.g. N-terminal or C-terminal tag without corresponding linker).

Note 2: The transcribed region always begins with the 4 base-pair grammar AATG. This is so the start codon methionine, encoded by the bases ATG, is already present in the grammar. It is thus not necessary to include an additional ATG start codon at the 5’ end of the gene-specific primer sequence at this position.

Here is the general design of a primer pair used to amplify a part for domestication:

Forward primer:

GCGCCGTCTCACTCGNNNNNNNNNNNNNNNNNNNNNNNNNNNNNNNNNN

Reverse primer:

GCGCCGTCTCACTCGNNNNNNNNNNNNNNNNNNNNNNNNNNNNNNNNNN

Extra bases to promote *Bsm*BI binding

*Bsm*BI restriction enzyme recognition site

Overhangs for ligation into the pUPD2 domestication vector

5’ overhang adapted to the grammar

3’ overhang adapted to the grammar

Gene-specific primer

Some examples of primer pairs with nonhomologous extensions that add grammatical sequences corresponding to common parts follow:

Promoter

Forward: GCGCCGTCTCACTCGGGAGNNNNNNNNNNNNNNNNNNNNNNNNNNNNNN

Reverse: GCGCCGTCTCACTCGCATTNNNNNNNNNNNNNNNNNNNNNNNNNNNNNN

N-terminal tag to be assembled with an N-terminal linker

Forward: GCGCCGTCTCACTCGAATGNNNNNNNNNNNNNNNNNNNNNNNNNNNNNN

Reverse: GCGCCGTCTCACTCGCACTNNNNNNNNNNNNNNNNNNNNNNNNNNNNNN

N-terminal tag to be assembled without an N-terminal linker

Forward: GCGCCGTCTCACTCGAATGNNNNNNNNNNNNNNNNNNNNNNNNNNNNNN

Reverse: GCGCCGTCTCACTCGGCTACCNNNNNNNNNNNNNNNNNNNNNNNNNNNNN

Coding sequence with no tags

Forward: GCGCCGTCTCACTCGAATGNNNNNNNNNNNNNNNNNNNNNNNNNNNNNN

Reverse: GCGCCGTCTCACTCGAAGCTTANNNNNNNNNNNNNNNNNNNNNNNNNNN

*ensure your primer includes the stop codon in this case

Coding sequence with N- and C-terminal tags and linkers

Forward: GCGCCGTCTCACTCGTAGCNNNNNNNNNNNNNNNNNNNNNNNNNNNNNN

Reverse: GCGCCGTCTCACTCGCCGANNNNNNNNNNNNNNNNNNNNNNNNNNNNNN

*ensure your primer does not include the stop codon in this case. The stop codon should only be included at the 3’ end of parts with the 3’ overhang GCTT. Otherwise, premature translation termination will occur.

Coding sequence with N-terminal tag and linker

Forward: GCGCCGTCTCACTCGTAGCNNNNNNNNNNNNNNNNNNNNNNNNNNNNNN

Reverse: GCGCCGTCTCACTCGAAGCTTANNNNNNNNNNNNNNNNNNNNNNNNNNN

*ensure your primer includes the stop codon in this case.

Coding sequence with C-terminal tag and linker

Forward: GCGCCGTCTCACTCGAATGNNNNNNNNNNNNNNNNNNNNNNNNNNNNNN

Reverse: GCGCCGTCTCACTCGCCGANNNNNNNNNNNNNNNNNNNNNNNNNNNNNN

*ensure your primer does not include the stop codon in this case. The stop codon should only be included at the 3’ end of parts with the 3’ overhang GCTT. Otherwise, premature translation termination will occur.

C-terminal tag to be assembled with a C-terminal linker

Forward: GCGCCGTCTCACTCGCTCGNNNNNNNNNNNNNNNNNNNNNNNNNNNNNN

Reverse: GCGCCGTCTCACTCGAAGCTTANNNNNNNNNNNNNNNNNNNNNNNNNNN

*ensure your primer includes the stop codon in this case

C-terminal tag to be assembled without a C-terminal linker

Forward: GCGCCGTCTCACTCGTCGGGCNNNNNNNNNNNNNNNNNNNNNNNNNNNNNN

Reverse: GCGCCGTCTCACTCGAAGCTTANNNNNNNNNNNNNNNNNNNNNNNNNNN

*ensure your primer includes the stop codon in this case

Terminator with no barcode

Forward: GCGCCGTCTCACTCGGCTTNNNNNNNNNNNNNNNNNNNNNNNNNNNNNN

Reverse: GCGCCGTCTCACTCGAGCGNNNNNNNNNNNNNNNNNNNNNNNNNNNNNN

1. Ensure that the part does not contain any *Bsm*BI or *Bsa*I recognition sites using sequence editing software. The presence of *Bsm*BI and *Bsa*I sites within parts decreases the efficiency of GoldenBraid cloning reactions because cuts at these sites must be re-ligated to yield correctly assembled vectors. If the part you wish to domesticate contains one or more recognition sites, you may choose to amplify it in multiple sub-parts called patches to abolish the recognition sites. At the following link: <https://gbcloning.upv.es/do/domestication/> you can check for *Bsm*BI and *Bsa*I recognition sites, and auto-generate primers that…
2. Abolish the *Bsm*BI and/or *Bsa*I restriction site(s) that are present in the part. The algorithm makes use of synonymous mutations in coding regions.
3. Have appropriate nonhomologous 5’ and 3’ overhangs that include *Bsm*BI restriction sites and 4 base-pair sticky ends that are compatible with the domestication reaction.

Notes:

1. The primer design for Dicty GoldenBraid requires one base change in the auto-generated reverse primer. The 15^th^ base in any reverse primer will be GCGCCGTCTCGCTCA. This base needs to be changed to GCGCCGTCTCGCTCG to be homologous with the 5’ and 3’ sticky overhangs present in pUPD2, which are both CTCG.
2. Often, it is not essential to abolish these extra sites as long as the 4-base-pair overhangs that they generate are not the same as others used in the GoldenBraid reaction.
3. PCR amplify your part(s) of interest using a standard protocol.

-We use a touchdown PCR protocol and a high-fidelity DNA polymerase like NEB Phusion for best results.

1. PCR or gel purify your amplicon(s) using a standard protocol.

-We use Zymo spin columns because of their small elution volumes. We elute into sterile water or TE.

1. Set up the domestication reaction.

This is a one-pot, combined reaction (restriction digestion and ligation) with the goal of inserting a given genetic part into a parental domestication vector backbone called the Universal Domesticator version 2, abbreviated as pUPD2. It is carried out in a thermocycler programmed to oscillate between 37°C and 16°C. The *Bsm*BI recognition sites present in pUPD2 and in the PCR amplicon(s) are oriented such that the enzyme can only cut the parental vector backbone, not the desired ligated product. In this way, each temperature cycle enriches for the desired product. This same enrichment process also occurs in all downstream GoldenBraid assembly reactions.


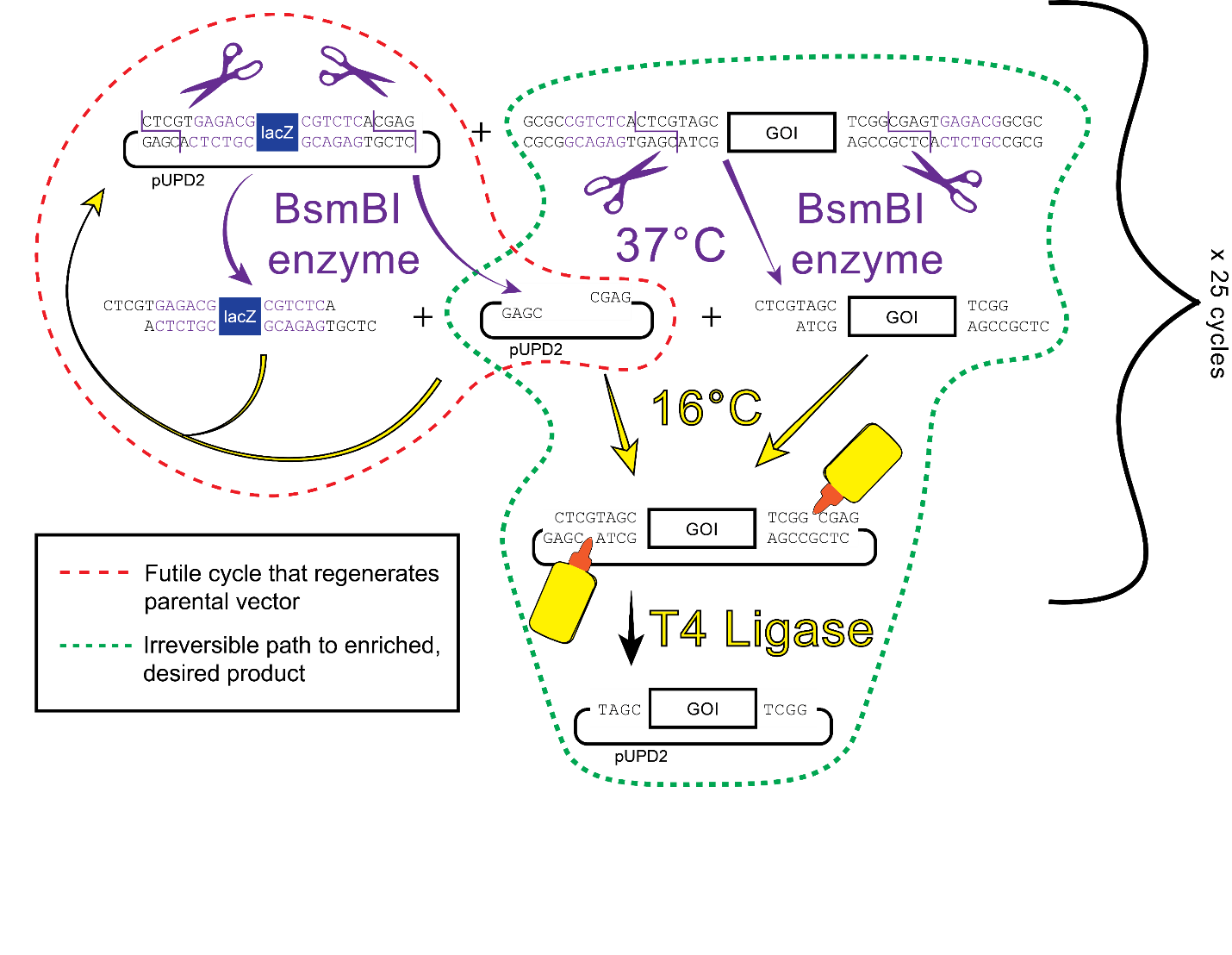


Into a PCR tube, add:

1 μl of pUPD2 parental vector backbone at 75 ng/μl concentration

X μl of PCR-amplified, purified part (or patch #1) to add a total of 40 ng

(Y μl of patch #2 to add a total of 40 ng, Z μl of patch #3 to add a total of 40 ng, etc.)

15 – X ( – Y – Z, etc.) μl of sterile water

2 μl of 10x T4 DNA ligase buffer

1 μl of T4 DNA ligase

1 μl *Bsm*BI restriction enzyme

…for a total 20 μl reaction.

(To save on reagents, you may halve all reagents above for a 10 μl reaction. We have found that this works equally well.)

Place the reaction in a thermocycler programmed as follows:

1. 2 min at 37°C
2. 5 min at 16°C
3. Repeat steps 1 and 2 for 24 additional cycles.
4. Optionally, you may incubate the reaction at 37°C for 5 minutes to overnight to cut remaining parental backbone and thereby reduce its rate of successful transformation. This is not essential because you can distinguish between clones harboring parental versus desired plasmids by blue/white screening using IPTG + X-gal. It only serves to increase the proportion of colonies transformed with the desired plasmid.

Note: If time is short, we have performed successful reactions with as few as 10 cycles.

1. Transform 1-5 μl of the combined reaction into chemically competent bacteria using a standard protocol.

-DH10β and DH5α host strains both maintain all tested vectors at high copy numbers without issue, but other host strains may be necessary for special applications.

-In general, only 1 µL of the combined reaction added to 10-15 µL of commercial competent cells is required to yield tens to several hundreds of transformants.

-Select for transformants on LB agar + 100 µg/mL **carbenicillin**- or **ampicillin**-containing 10-cm plates, each top-plated with 50 µL of 40 mg/mL X-gal in DMF or DMSO and 50 µL of 0.1 M IPTG.

-The parental vector pUPD2’s resident insert contains *lacZα* driven by the constitutive *em7* promoter, so colonies transformed with it appear blue. Colonies with the desired insert have lost the resident insert containing *em7/lacZα* and appear white.

1. After overnight growth on plates, pick 1-3 white colonies to grow overnight shaking in LB media containing 100 µg/mL carbenicillin or ampicillin, then miniprep using a standard protocol.

-Starting from a 4 mL overnight culture, our plasmid yields are usually 6-8 μg in total (~130 ng/μl eluted in 50 μl TE or sterile water) per Qiagen column prep. However, this depends on the vector’s identity and size, with larger vectors typically yielding more DNA.

1. Verify that the insert is the correct size by diagnostic digest with EcoRI, which cuts immediately on either side of the insert.

-In absence of additional EcoRI sites within the insert, this reaction should yield a part-sized fragment and a ~2.9 kb pUPD2 backbone fragment.

-You may wish to perform additional diagnostic digests using restriction enzyme(s) that perhaps cut within the insert.

1. Send the vector containing your size-confirmed part for Sanger sequencing using the M13F and/or M13R universal primers.

-SP6 and T7 may also be used, but their binding sites are located slightly closer to the insert, so using them poses an increased risk of missing important grammatical sequences early in the traces.

-Large parts (>~1 kb) may require additional custom interior sequencing primers.

-The grammatical positions that are ligated between the part and the pUPD2 backbone in the domestication reaction are both CTCG. This means that the part can ligate in two possible orientations into pUPD2. This does not affect downstream assembly reactions.

Protocol 2: Assemble transcriptional units in α-level assembly vectors

1. Determine the desired transcriptional unit to assemble from parts found in pUPD2 domestication level backbones. Consult dictyBase for an up-to-date list of pre-existing domesticated parts and their corresponding grammars. If one or more necessary parts have already been domesticated, order them to be sent to your laboratory.
2. Determine the desired backbone.

-All assembly vectors are modified versions of the pBluescript backbone. α-level assembly vectors are abbreviated starting with pDGB_α for “Dicty/destination GoldenBraid, α-level”. We have changed the “α” symbol to “A” in filenames to avoid using this special character.

-There are a total of six assembly-level grammatical positions in the α level of assembly vectors. These are designated α1, α2, αB, αC, αD, and αE. The choice of this grammatical position determines whether and in which order the assembled transcriptional unit can be combined with other units into an Ω-level backbone. The details of these reactions will be discussed comprehensively in Protocol 3. We recommend understanding Protocol 3 in addition to this protocol before starting any α-level assembly reactions because this greatly informs decision-making as to which α-level backbone to use in a given reaction.

-Each α-level assembly vector has a version that includes one of three *Dictyostelium* positive selectable markers (*neoR*, *hygR*, or *bsR*) driven by the *coaA* promoter built into the vector backbone. These are designated as N, H, or B, respectively, after the assembly level (α or Ω) and specific grammatical position (1,2, B, C, D, or Z) of the vector.

For example, pDGB_α1N designates α as the assembly vector level, 1 as the assembly-level grammatical position, and *coaA/neoR* as the *Dictyostelium* positive selectable marker built into the vector backbone. To strip away unnecessary detail, we have not included the designation of the *Dictyostelium* positive selectable marker in all following figures.

An example of an α-level assembly reaction follows:


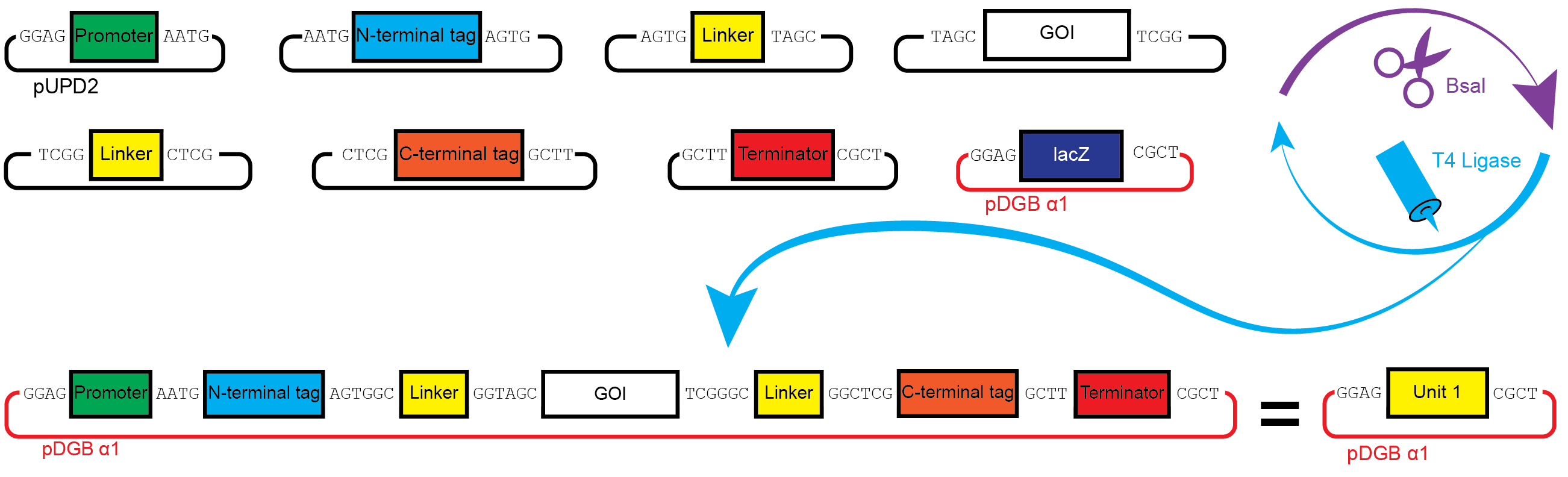


Your reaction may include additional or fewer parts in pUPD2 depending on your desired transcriptional unit.

1. Set up the combined restriction digestion and ligation reaction.

Into a PCR tube, add:

1 μl of pDGB α1 or α2 or αB or αC or αD or αE at 75 ng/μl concentration

X μl of part 1 in pUPD2 to add a total of 40 ng,

Y μl of part 2 in pUPD2 to add a total of 40 ng,

Z μl of part 3 in pUPD2 to add a total of 40 ng, etc.

15 μl - X - Y - Z etc. μl of sterile water

2 μl of 10x T4 DNA ligase buffer

1 μl of T4 DNA ligase

1 μl *Bsa*I restriction enzyme

…for a total 20 μl reaction.

(To save on reagents, you may halve all reagents above for a 10 μl reaction. We have found this works equally well.)

Place the reaction in a thermocycler programmed as follows:

1. 2 min at 37°C
2. 5 min at 16°C
3. Repeat steps 1 and 2 for 24 additional cycles.
4. Optionally, you may incubate the reaction at 37°C for 5 minutes to overnight to cut remaining parental backbone and thereby reduce its rate of successful transformation. This is not essential because you can distinguish between parental and desired plasmids by blue/white screening using IPTG + X-gal. It only serves to increase the proportion of colonies transformed with the desired plasmid.

Note: If time is short, we have performed successful reactions with as few as 10 cycles.

4. Transform 1-5 μl of the combined reaction into chemically competent cells using a standard protocol.

-DH10β and DH5α host strains both maintain all tested vectors at high copy numbers without issue, but other host strains may be necessary for special applications.

-In general, only 1 µL of the combined reaction added to 10-15 µL of commercial competent cells is required to yield tens to several hundreds of transformants.

-Select for transformants on LB agar + 30 µg/mL **kanamycin**-containing 10-cm plates,

each top-plated with 50 µL of 40 mg/mL X-gal in DMF or DMSO and 50 µL of 0.1 M IPTG.

-The parental vector pDGB_α#X’s resident insert contains *lacZα* driven by the constitutive *em7* promoter, so colonies transformed with it appear blue. Colonies with the desired insert have lost the resident insert containing *em7/lacZα* and appear white.

-Any cells transformed with intact parental pUPD2 plasmids will not survive because they are only resistant to ampicillin/carbencillin, not kanamycin.

5. After overnight growth on plates, pick 1-3 white colonies to grow overnight shaking in LB media containing 30 µg/mL kanamycin, then miniprep using a standard protocol.

-Starting from a 4 mL overnight culture, our plasmid yields are usually 6-8 μg in total (~130 ng/μl eluted in 50 μl sterile water) per Qiagen column prep, but this depends on the vector’s identity and size, with larger vectors typically yielding more DNA.

6. Verify your transcriptional unit is the correct size by diagnostic digest with the appropriate insert-flanking enzyme, or other enzyme(s) of your choice:

α1: EcoRI

α2, αB, αC, αD, αE do not have dedicated unique insert-flanking enzymes, so you must fingerprint them on a case-by-case basis.

-You may also wish to digest with another enzyme that perhaps cuts within your insert.

-Sanger sequencing is not necessary unless a functional issue arises downstream because each of the individual parts are already sequenced in the pUPD2 backbone. To date, we have not experienced any such functional issues.

Protocol 3: Combine transcriptional units by shuttling between α- and Ω-level backbones

Initial assembly reactions into α-level backbones usually yield single transcriptional units as inserts. These can be combined into a single Ω-level backbone either two-at-a-time in a traditional α-to-Ω assembly reaction, or five-at-a-time in an expanded α-to-Ω assembly reaction. Both assembly reactions use *Bsm*BI restriction enzyme.

Ω1 and Ω2 inserts, which initially comprise multiple transcriptional units that have been combined from α-level vectors, can only be assembled in a traditional, two-at-a-time, Ω-to-α assembly using *Bsa*I restriction enzyme.

Ω-level assembly vectors are abbreviated starting with pDGB_Ω for “Dicty/destination GoldenBraid, Ω-level”. We have changed the “Ω” symbol to “O” in filenames to avoid using this special character.

Repeated shuttling between α- and Ω-level backbones allows individual transcriptional units and groups of transcriptional units to be combined into single backbones theoretically *ad infinitum*. In reality, vector capacity constrains the amount of transcriptional units that can be assembled into a single backbone. Our collaborators have successfully maintained pDGB assembly vectors of up to ~25 kb in DH10β cells, but 12-15 kb may be a safer average size based on general experience with AT-rich *D. discoideum* DNA.

The traditional α-to-Ω assembly

In this assembly reaction, transcriptional units are combined according to the following rules:

1. α1 and α2 units can only be combined into Ω1 or Ω2 backbones.
2. They will always ligate in the order:

5’ – Unit from α1 – Unit from α2 – 3’

In this way, two α-level units will be combined into a single Ω-level backbone.

An example of a traditional α-to-Ω assembly reaction follows:


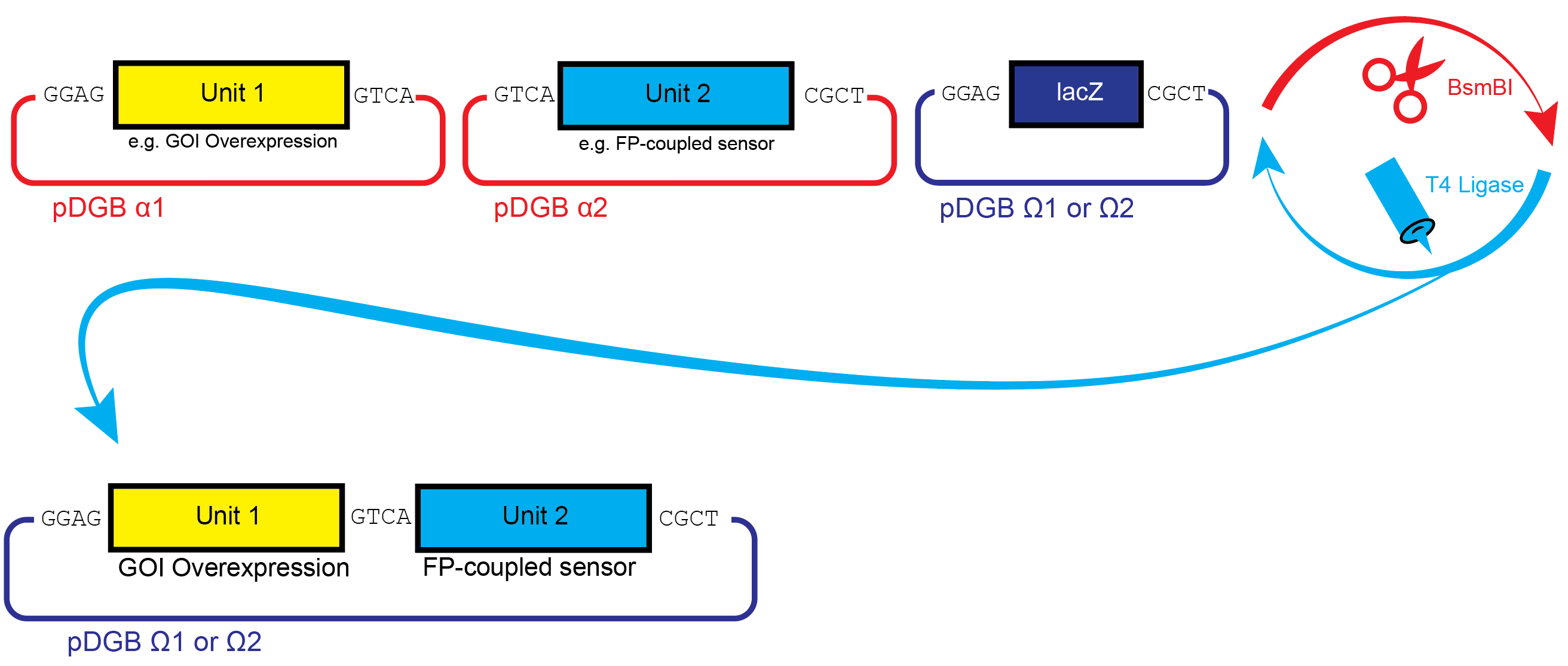


The expanded α-to-Ω assembly

In this assembly reaction, transcriptional units are combined according to the following rules:

1. α1, αB, αC, αD, and αE units can only be combined into Ω1 or Ω2 backbones.
2. They will ligate in the order:

5’ – Unit from α1– Unit from αB – Unit from αC – Unit from αD – Unit from αE – 3’

In this way, five α-level units will be combined into a single Ω-level backbone.

An example of an expanded α-to-Ω assembly reaction follows:


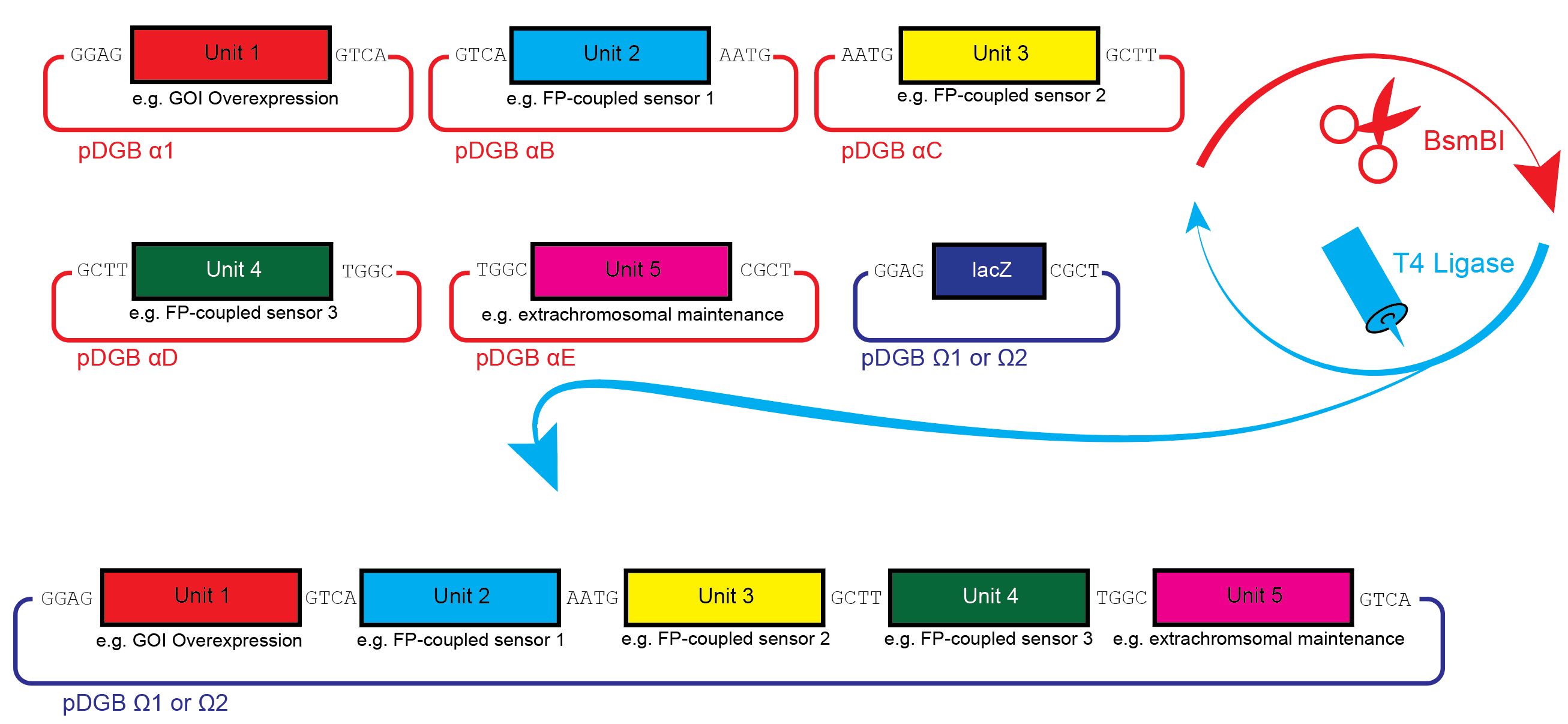


The expanded α-to-Ω assembly allows more transcriptional units to be combined in a single reaction. However, it possesses the minor drawback that, regardless of how many transcriptional units are desired in the final vector (2, 3, 4, or 5), all five α-level units (units from α1, αB, αC, αD, and αE) must be combined to generate the desired plasmid. This is the case because of the specific assembly-level grammar necessary to ligate the units into the backbone. For this reason, we have developed α-level vectors with insert units that comprise short, non-coding multiple cloning sites called stuffers.

For example, imagine that you want to combine 3 inserts that are currently in backbones α1, αB, and αD. In this case, because of the specific assembly grammar for the expanded α-level vectors, you cannot simply ligate α1-unit1, αB-unit2, and αD-unit3 together into the Ω1 or Ω2 backbone. Instead, you would need to add to the combined reaction both αC and αE backbones, each containing a stuffer insert that would appear in the final Ω-level plasmid as:

5’ –Unit 1 from α1 –Unit 2 from αB –Stuffer from αC –Unit3 from αD – Stuffer from αE – 3’

This also applies to the traditional α-to-Ω assembly if you want only to shuttle a transcriptional unit from an α-level to an Ω-level backbone without adding another unit. In the combined reaction, you must include the second α-level vector that contains a stuffer insert.

The Ω-to-α assembly

Ω1 and Ω2 inserts, which usually comprise multiple transcriptional units that have been combined from α-level vectors, can only be assembled in a traditional, two-at-a-time, Ω-to-α assembly using *Bsa*I. To date, there is no expanded set of Ω-level vectors. This assembly reaction operates according to the following rules:

1. Ω1 and Ω2 inserts can only be combined into α-level (α1, α2, αB, αC, αD, or αE) backbones.
2. They will ligate in the order:

5’ – Insert from Ω1 – Insert from Ω2 – 3’

In this way, two Ω-level inserts will be combined into a single α-level backbone.

An example of an Ω-to-α assembly reaction follows:


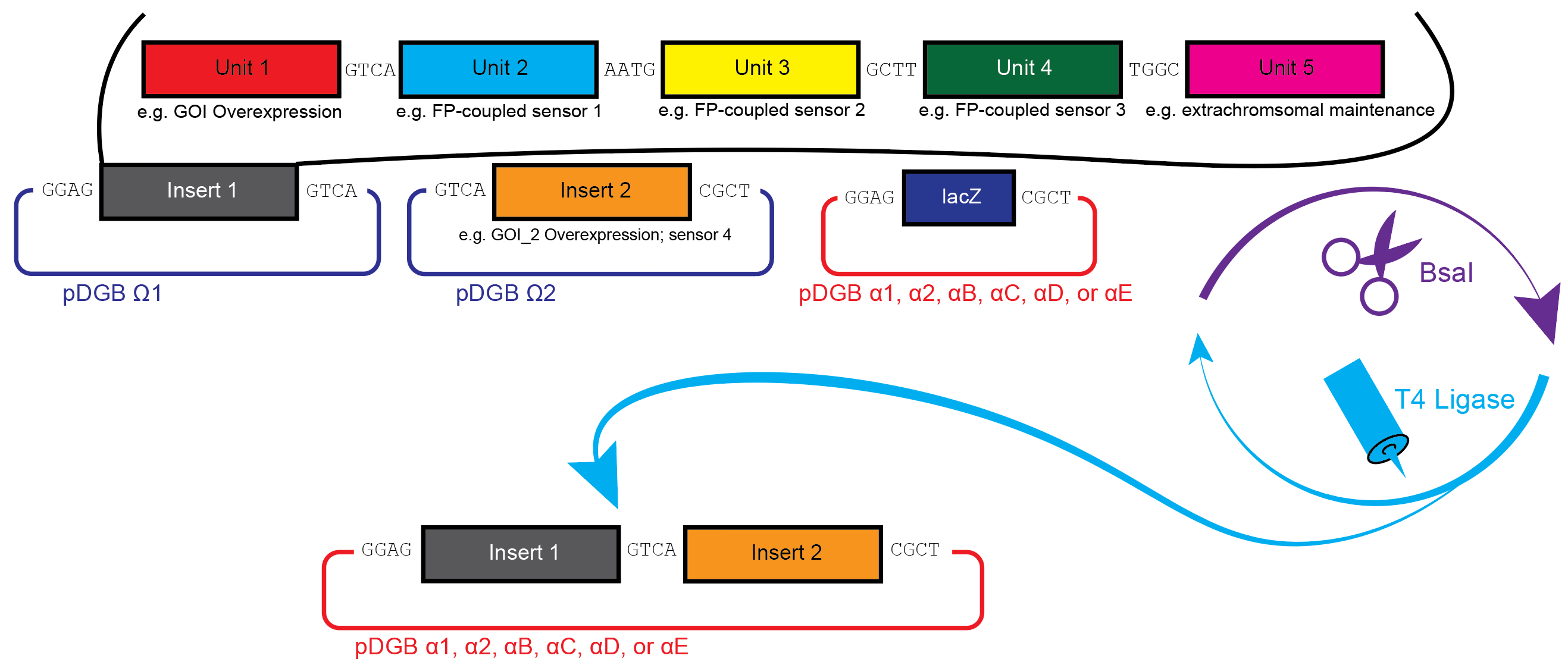


Like with α-level assemblies, because of the specific assembly grammar, an insert from Ω1 must be combined with an insert from Ω2 to successfully ligate into an α-level backbone. If you want only to shuttle an insert from an Ω-level backbone to an α-level backbone without adding a second insert, you must include in the combined reaction the second Ω-level backbone with a stuffer insert. These Ω-level stuffers are available from dictyBase.

1. Set up the combined restriction digestion and ligation reaction.

Into a PCR tube, add:

1 μl of the desired backbone: α1, α2, αB, αC, αD, αE, Ω1, or Ω2 at 75 ng/μl concentration

V μl of the plasmid containing your desired unit1 or insert1 (either α1 or Ω1) to add a total of 40 ng

W μl of the plasmid containing unit2 or insert2 (either α2, αB, or Ω2, same level backbone as unit1 or insert1) to add a total of 40 ng

(If applicable, for the expanded α-to-Ω assembly, X μl of the plasmid containing unit3 to add a total of 40 ng, Y μl of the plasmid containing unit4 to add a total of 40 ng, and Z μl of the plasmid containing unit5 to add a total of 40 ng)

15 – V – W – X – Y – Z μl of sterile water to 20 μl total

2 μl of 10x T4 DNA ligase buffer

1 μl of T4 DNA ligase

1 μl *Bsa*I restriction enzyme if the destination backbone is pDGB α-level;

or…

1 μl *Bsm*BI restriction enzyme if the destination backbone is pDGB Ω-level

(To save on reagents, you may halve all reagents above for a 10 μl reaction. We have found this works equally well.)

Place the reaction in a thermocycler programmed for:

1. 2 min at 37°C
2. 5 min at 16°C
3. Repeat steps 1 and 2 for 24 additional cycles.
4. Optionally, you may incubate the reaction at 37°C for 5 minutes to overnight to cut remaining parental backbone and thereby reduce its rate of successful transformation. This is not essential because you can distinguish between parental and desired plasmids by blue/white screening using IPTG + X-gal. It only serves to increase the proportion of colonies transformed with the desired plasmid.

Note: If time is short, we have performed successful reactions with as few as 10 cycles.

1. Transform 1-5 μl of the combined reaction into chemically competent cells using a standard protocol.

-DH10β and DH5α host strains both maintain all tested vectors at high copy numbers without issue, but other host strains may be necessary for special applications. In general, only 1 µL of the combined reaction added to 10-15 µL of commercial competent cells is required to yield tens to several hundreds of transformants.

-Select for transformants with **α-level** destination backbones on LB agar + 30 µg/mL **kanamycin**-containing 10-cm plates, each top-plated with 50 µL of 40 mg/mL X-gal in DMF or DMSO.

-Select for transformants with **Ω-level** destination backbones on LB agar + 12.5 µg/mL **chloramphenicol**-containing 10-cm plates, each top-plated with 50 µL of 40 mg/mL X-gal in DMF or DMSO and 50 µL of 0.1 M IPTG.

-The parental vector’s resident insert contains *lacZα* driven by the constitutive *em7* promoter, so colonies transformed with it appear blue. Colonies with the desired insert have lost the resident insert containing *em7/lacZα* and appear white.

-Any cells transformed with intact parental backbone will not survive because they are only resistant to the antibiotic that selects for backbones of the opposite assembly level.

3. After overnight growth on plates, pick 1-3 white colonies to grow overnight in LB media containing 30 µg/mL kanamycin for α-level backbones or 12.5 µg/mL chloramphenicol for Ω-level backbones then miniprep using a standard protocol.

-Starting from a 4 mL overnight culture, our plasmid yields are usually 6-8 μg in total (~130 ng/μl eluted in 50 μl sterile water) per Qiagen column prep, but this depends on the vector’s identity and size, with larger vectors typically yielding more DNA.

4. Verify your insert is the correct size by diagnostic digest with the appropriate insert-flanking enzyme, or other enzyme(s) of your choice:

α1: EcoRI

α2, αB, αC, αD, αE do not have dedicated insert-flanking enzymes, so you must fingerprint them on a case-by-case basis.

Ω1: BamHI

Ω2: PstI

-You may also wish to digest with another enzyme that cuts internally within your insert.

-Sequencing is not necessary unless a functional issue arises downstream because each of the individual parts are already sequenced. To date, we have not experienced any such functional issues.

Additional Notes

1. We suggest using Promega T4 DNA ligase in GoldenBraid cloning reactions. Our collaborator has found that the Promega formulation of the 10x T4 DNA ligase buffer produces slightly better results than competing buffers.
2. Standard *Bsa*I and *Bsm*BI enzymes ordered from NEB have worked well in our lab for GoldenBraid reactions. NEB also offers *Bsa*I-HF and *Bsa*I-HFv2. The latter is specifically optimized for Golden Gate cloning reactions. We have not had need of the potentially increased cutting efficiencies of these versions of *Bsa*I.
3. *Bsm*BI’s ideal cutting temperature is 55°C, but it does retain some cutting acitivity at 37°C. Unfortunately, exposure to 55°C temperatures progressively denatures T4 DNA ligase. To date, we have not experienced issues with insufficient cutting efficiency of *Bsm*BI at 37°C. If you do experience an issue, you can add an extra 0.5-1 μl of *Bsm*BI to the offending reaction to increase the rate of cutting. Alternatively, NEB offers the *Bsm*BI isoschizomer Esp3I, which is most active at 37°C, and at the time of writing is cheaper than *Bsm*BI. We have not yet tested this enzyme for efficacy in 1x T4 DNA ligase buffer.

Protocol 4: *Dictyostelium* transformation

Reagents

Parental strain of *Dictyostelium* growing in HL5 + pen, strep, vitamins (PSV) shaking culture

HL5 media + PSV

0.4 cm gap cuvettes

50 mL Falcon tubes

10 cm culture dishes

Centrifuge (optionally refrigerated)

Optional: Ice bucket + ice

EP buffer

10 mM NaPO_4_

50 mM sucrose

pH to 6.1 using NaOH or phosphoric acid, depending on form of NaPO_4_ used

Autoclave or filter-sterilize

Stock solution of selective drug

- Blasticidin S hydrochloride: 10 mg /mL pre-prepared solution (1:1000 dilution for final concentration in HL5; we routinely use 4 ug/mL final concentration and still select successfully)
- Hygromycin B: 25 mg /mL in 10 mM HEPES12 pre-prepared solution (1:1000 dilution for final concentration in HL5)
- G418 sulfate: 10 mg /mL pre-prepared solution (1:1000 dilution for final concentration in HL5)

Procedure

1. Using sterile technique, collect *Dictyostelium* cells at 2-4x10^6^ cells/mL density in HL5. 5x10^6^ to 1x10^7^ cells total are needed per transformation.
2. Place the necessary volume of the *Dictyostelium* culture on ice for 5-20 min. Optionally, pre-chill a refrigerated centrifuge to 4^o^C and one 0.4 cm gap cuvette per transformation on ice. I see good results both with and without pre-chilling.
3. Aliquot 25 mL of HL5 in a Falcon tube per transformation and optionally chill at 4^o^C.
4. Label two 10 cm culture dishes per transformation with the final genotype of the strain to be transformed and the date.
5. Spin at low speed (~1250-1500 rpm for 2-3 min) to pellet *Dictyostelium* cells. Decant all HL5 media.
6. Add 0.5-1x of the original culture volume of EP buffer, resuspend cells, then spin again at low speed to pellet. Decant the EP buffer. Optionally, repeat this wash step one more time.
7. Resuspend cells in 720-800 μL EP buffer per transformation. Pipette each 720-800 μL cell suspension (each containing 1x10^7^ cells) into separate Eppendorf tubes. To each tube add up to 80 uL of plasmid DNA to total 800 uL. The amount of plasmid DNA added depends on the type of vector:

-For extrachromosomal vectors, add ~3-10 ug DNA per transformation

-For integrating and knock-out vectors, whether circular or linearized, add 7-15 ug DNA per transformation

1. After adding DNA, mix well by pipetting.
2. Transfer the mixed 800 uL total volume to a 0.4 cm gap cuvette. Keep on ice until electroporation. Wipe off condensation on the metal sides of the cuvette before electroporating with a KimWipe.
3. Electroporate with settings: exponential decay, 1000 V, 25 ohm resistance, 50 uF capacitance, two pulses with 5 second interval. Place the cuvettes back on ice after electroporation.
4. Add the transformed *Dictyostelium* cells to the optionally pre-chilled 25 mL of HL5. Divide the cells and media evenly between the two 10 cm culture dishes at 12.5 mL per dish.
5. Leave the plates without drug treatment for 16-24 hours so cells can recover and start to express the antibiotic resistance gene.
6. At 16-24 hours, view the cells under a light microscope. By this time, the majority of cells should have settled to the bottom of the dish and become adherent and amoeboid in shape. If nearly all cells are floating and rounded up, they are likely dead or dying and you should repeat the transformation.
7. Add appropriate drug selection.
8. Leave the plates at rest so the drug selection can occur. For blasticidin, resistant cells should begin to form small colonies by 4-5 days of selection. For hygromycin and neomycin, small colonies may only appear after 7-10 days of selection.
9. Change the media containing the selective drug every 3 days.
10. If necessary, once the colonies have grown sufficiently, isolate individual clones on bacterial plates or in HL5 media.

Notes:

1. The transformation efficiencies and (where applicable) integration efficiencies of individual plasmids are highly variable. With integrating plasmids, sometimes I’ll see tens to hundreds of colonies after only ~4 days of selection with G418. Sometimes only several colonies will appear by day 10. Sometimes a high proportion of the initially tens to hundreds of colonies will persist and expand. Sometimes by day 10-12 only a handful of the initially hundreds of colonies will still be present and have expanded a little before their growth rate finally takes off. My suspicions as to why this variability exists are:
   1. Differences in DNA prep quality before transformation. I like column preps as opposed to other plasmid isolation and purification methods.
   2. Differences in transformation efficiencies due to slight differences in the final buffer composition (after adding plasmid in water or TE), electroporation parameters (which vary by each shock even when the same settings are applied), plasmid size, plasmid linearization, temperature(?), etc.

The above two classes of parameters influence how many colonies appear initially.

- 1. Differences in integration efficiencies, possibly due to plasmid size, linearization, microhomology, idiosyncrasies of interaction with the *Dictyostelium* DNA repair machinery, etc.

This class of parameters influences how many colonies ultimately integrate the plasmid and stably resist the selective drug. Of course, the ultimate number of stable transformants also depends on the transformation efficiency. High transformation efficiencies can lead to high rates of false positives—cells that are transformed, but have not integrated the plasmid. I find it can take up to 2-3 weeks in the most prolonged of cases to dilute out the transformed plasmid over rounds of cell divisions. It has no DdOri, so hopefully it isn’t being replicated.

1. I don’t linearize any plasmid (even integrating ones) before transformation. Linearization probably reduces the numbers of false positives, but I don’t like having to digest large amounts of DNA and often losing a significant amount of it during purification.
2. Many protocols now use square wave electroporation. It is probably better than exponential decay but I do not have access to an electroporator with this setting.
3. *Dictyostelium* densities of 2-4E6 cells/mL are probably ideal, but I often transform cells successfully between densities of 7E5 to 1E7. At least with this protocol, the initial *Dictyostelium* cell density doesn’t seem to matter much.

Recommendations for *Dictyostelium* transformation:

1. If the majority of cells are phase-bright, circular, and floating (versus phase-darker, amoeboid, and adhered) 16-24 hours after transformation, they are dying or dead and a problem likely occurred with the electroporation. Dispose of them and try again. One reason I use EP instead of H50 or H40 is that in my hands it consistently kills less cells during the initial electroporation. H50 and H40 probably result in higher rates of transformation, but at least with the electroporation settings I use, cell death by electroporation usually more than outweighs these gains. Also, higher rates of false positives are annoying.
2. Do not trust that any colonies you see on your plates are stable transformants until at least 10-12 days of drug selection. Often you can see adherent, amoeboid cells even after 2-3 days of drug selection. These are a good sign, but they can be false positives.
3. If you see no colonies by day ~8 of selection, dispose of the plates and try again.
