# Supplementary material for: A GoldenBraid cloning system for synthetic biology in social amoebae
Source: Nucleic Acids Res. 2020 Mar 30;48(8):4139–46. doi: 10.1093/nar/gkaa185 (PMC7192589; doi:10.1093/nar/gkaa185)
Supplement: gkaa185_Supplemental_Files [file gkaa185_supplemental_files.zip › Kundert_Supplementary Table 5.pdf]

Functional category of parts	Individual parts	Count	Grammar class
Overexpression promoter	coaA, act15, act6	3	Promoter class
Putative light-sensitive promoter	aurK, thyA, 429	3	Promoter class
Developmental promoter	tgrBC, dscA	2	Promoter class
Cell-type-specific promoter	ecmA	1	Promoter class
Cre-loxP	loxP-coaAp, mhcA-loxP	1p, 1t	Promoter class, Terminator class
Fluorescent protein	tagBFP, mCerulean, sfGFP, mNeonGreen, eYFP, mCherry, mIFP	7	CDS (3-part) class
Fluorescent tag	tagBFP, mCerulean, sfGFP, mNeonGreen, eYFP, mCherry, mIFP	7	Tag class
Photoswitchable FP	dendra2	1	CDS (3-part) class
Luciferase	FfLuc, renilla	2	CDS (3-part) class
Affinity tag	7xHis, Myc, HA, GST	4	Tag class
Flexible linker	GGGSx4, FlexLink	2	Linker class
Terminator	mhcA, act8	2	Terminator class
Barcode	BC1-20	20	Terminator class
Fusion proteins	ctsZ, 295, 731, gghA, dscA (5- and 7-part), gtaC	6	CDS (5-part) class
NLS	SV40	1	Tag class
Nuclear marker	H2Bv3 (7-part), hcpB (5-part)	2	CDS class
Leading edge marker	PH	1	CDS (5-part) class
Trailing edge marker	abpC	1	CDS (5-part) class
cAMP sensor	flamindo2, pink flamindo	2	CDS (3-part) class
Rap-activation sensor	ralGDS	1	CDS (5-part) class
Antibiotic selection	bsR, hygR, neoR	3	CDS (3-part) class

α1 functionally distinct assemblies enumeration

Promoter classes	Tag1 classes	Link1	CDS classes	Link2	Tag2 classes	Terminator/ barcode	Assumptions
N/A	N/A	N/A	N/A	N/A	N/A	N/A	N- and C-terminal fusiosn are functionally equivalent
N/A	N/A	N/A	N/A	N/A	N/A	N/A	Cre-loxP is w/ promoter classes
5	N/A	N/A	5	N/A	N/A	2	NLS linker function excluded from class
5	4	1	6	N/A	N/A	2	CRISPR functions excluded
5	4	1	2	1	3	2	

α2 functionally distinct assemblies enumeration

Promoter classes	Tag1 classes	Link1	CDS classes	Link2	Tag2 classes	Terminator/ barcode	Assumptions
N/A	N/A	N/A	N/A	N/A	N/A	N/A	Non-redundant functions as approximated by at least one different tag and CDS
N/A	N/A	N/A	N/A	N/A	N/A	N/A	Choice of backbone does not impact function
5	N/A	N/A	4	N/A	N/A	2	Barcodes count as a single class
5	3	1	5	N/A	N/A	2	CRISPR functions excluded
5	3	1	1	1	3	2	

α1 functionally distinct assemblies calculation

1-part	1
2-parts	0
3-parts	50
4- and 5-parts	240
6- and 7-parts	240
**Total**	531

α2 functionally distinct assemblies calculation

1-part	1
2-parts	0
3-parts	40
4- and 5-parts	150
6- and 7-parts	90
**Total**	281

Ω1 functionally distinct assemblies calculation

2-part, total	149211
